# Genome-wide identification of glutathione S-transferase gene family in pepper, its classification, and expression profiling under different anatomical and environmental conditions

**DOI:** 10.1038/s41598-019-45320-x

**Published:** 2019-06-24

**Authors:** Shiful Islam, Saikat Das Sajib, Zakya Sultana Jui, Shatil Arabia, Tahmina Islam, Ajit Ghosh

**Affiliations:** 1 Department of Biochemistry and Molecular Biology, Shahjalal University of Science and Technology, Sylhet, 3114 Bangladesh; 2 Plant Breeding and Biotechnology Laboratory, Department of Botany, University of Dhaka, Dhaka, 1000 Bangladesh; 3 Max-Planck Institute for Plant Breeding Research, Carl-von-Linne-Weg 10, D-50829 Cologne, Germany

**Keywords:** Plant evolution, Abiotic

## Abstract

Glutathione S-transferases (GSTs) compose a family of multifunctional enzymes involved in the numerous aspects of regulating plant growth, development, and stress response. An *in silico* genome-wide analysis of pepper (*Capsicum annuum* L.) was performed to identify eighty-five *GST* genes that were annotated according to their chromosomal location. Segmental duplication contributed more than tandem duplication for the expansion of *GST* gene family in pepper. All the identified members belong to ten different classes which are highly conserved among *Arabidopsis*, rice, tomato and potato counterparts indicating the pre-dicot-monocot split diversification of GST classes. Gene structure, protein domain, and motif organization were found to be notably conserved over the distinct phylogenetic groups, which demonstrated the evolutionary significant role of each class. Expression of most of the *CaGST* transcripts as well as the total pepper GST activity was found to be significantly up-regulated in response to cold, heat, drought, salinity and osmotic stress conditions. Presence of various hormone and stress-responsive cis-elements on most of the putative *CaGST* promoter regions could be directly correlated with the alteration of their transcripts. All these findings might provide opportunities for future functional validation of this important gene family in pepper.

## Introduction

Cellular detoxification is an elementary biological process in all living organisms including animals, plants, and microorganisms. It provides protection against different environmental noxious agents as well as reactive oxygen species (ROS) formed from different metabolic pathways, to ensure the optimum cellular condition for the growth and survival^1^. After the conversion of toxic components into reactive electrophiles by cytochrome P450s, these reactive molecules are subsequently transformed by the exertion of phase II enzymes, namely Glutathione S-transferase (GST) for the final degradation process^2^. GSTs are metabolic isozymes that form a complex family with versatile functions in plants^3^. Among its myriad functions, this family mainly works as a detoxification route in plants by conjugating glutathione (γ-Glu-Cys-Gly) to a diverse range of hydrophobic, electrophilic, xenobiotic compounds and redox buffering to form a soluble S-glutathionylated (R-SG) products^4^. This gene family possesses a high transcript abundance in most of the tissues and thus, act as a biomarker for the detection and monitoring of organ/tissue damage both in plants and animals^1,5^.

*GST* genes are abundantly found in animals, plants and even in some prokaryotes^6^. The family consists of cytosolic, mitochondrial and microsomal GSTs. Among them, the mitochondrial and microsomal members showed significant differences in their sequence and biosynthesis process as compared with the cytosolic GSTs^7^. Classification of the plant soluble GST members is evaluated based on the sequence conservance, genomic organization, kinetic and physiochemical properties, and immunological cross relativeness^8^. There are fourteen classes of GSTs found so far in plants, including tau (U), phi (F), lambda (L), dehydroascorbate reductase (DHAR), theta (T), zeta (Z), eukaryotic translation elongation factor 1B- γ subunit (EF1Bγ), tetra-chloro hydroquinone dehalogenase (TCHQD), microsomal prostaglandin E-synthase type 2 (mPGES-2), glutathionyl hydroquinone reductase (GHR), metaxin, Ure2p, hemerythrin (H) and iota (I)^8^. The first four of them (tau, phi, lambda, DHAR) and recently identified two new classes (hemerythrin and iota) are highly plant-specific^8^.

Tau and phi classes have drawn much interest as they are the most abundant classes in plants and perform a major role in the xenobiotics metabolism^9^. Theta-class of GST is mainly involved in the oxidative metabolism and zeta GSTs convert maleylacetoacetate to fumarylacetoacetate in a glutathione-dependent reaction^10^. The lambda and DHAR function as thiol transferases by replacing its serine residue to cysteine^11,12^. The five other classes of GST, such as mPGES-2, GHR, metaxin, hemerythrin, and iota possess cysteine in their active site. Catalytic mechanism of Ure2p is mediated by the asparagine residue which plays a key role in the glutathione stabilization^13^. However, the catalytic nature of the EF1Bγ class is not clear to date^8^.

Previous studies have emphasized the involvement of GSTs in various abiotic stress response^14,15^. The tau class GSTs perform a potential role against the oxidative damages, chemical toxicity, and physical stress agents^16,17^. Over-expression of *TaGSTU*1B and *TaGSTF*6 genes in wheat enhanced tolerance against drought^18^. Ectopic expression of a rice tau glutathione s-transferase, *OsGSTU*4 improves tolerance to salinity and oxidative stresses in *Arabidopsis* by up-regulating several stress responsive and cellular detoxifying genes^19^. Similarly, ectopic expression of stress-inducible *GmGSTU*4, *CsGSTU*1 and *CsGSTU*2 transcripts in transgenic tobacco plants enhanced tolerance against diphenyl ether herbicide, salinity, and drought stresses^20,21^.

Genome-wide analysis of *GST* gene family had been carried out in various plant species, and identified 55 members in *Arabidopsis*^15^, 84 in barley^22^, 65 in *Brassica oleracea*^23^, 49 in *G. arboretum* and 59 in *G. raimondii*^24^, 27 in Japanese larch^25^, 42 in maize^26^, 62 in China-pear^27^, 90 in potato^28^, 37 in *Physcomitrella patens*^29^, 81 in populus^30^, 79 in rice^14^, 101 in soybean^31^, 14 in sunflower^32^, 23 in sweet orange^33^, 73 in *Medicago*^34^, 49 in *Capsella*^35^ and 90 in tomato^36^. Although detailed genome sequence information of *Capsicum annuum* is publicly available, genome-wide analysis of *GST* gene family was not performed yet. Pepper has a relatively large genome size of 3.48 Gb with a life cycle of around 95 days^37^. In this study, a comprehensive genome-wide analysis of GSTs has been accomplished in *Capsicum annuum* and identified a total of 85 members. Analyses of chromosomal position, physiochemical characteristics, conserved motifs, and subcellular localization of these identified members showed a great variation among themselves. An evolutionary trajectory is drawn between pepper and one of its closest relative tomato, based on their class wise sequence analysis. Furthermore, expression patterns of all the identified genes and total GST activity were analyzed under different developmental and environmental conditions. This study will facilitate a door for the researchers to identify the specific gene/member of the family for crop improvement and stress management.

## Results

### Identification and nomenclature of *GST* genes in *Capsicum annuum*

Eighty-five full length genes encoding putative GST proteins were identified in *C. annuum* and classified into ten classes: tau, phi, theta, zeta, lambda, EF1Bγ, DHAR, TCHQD, MGST, and GHR. The tau and phi classes are found to be the most abundant with 59 and 6 members, respectively (Table 1). The length of the *CaGST* transcripts ranged from 306 bp (*CaGSTU*29) to 14430 bp (*CaGHR*1), whereas the deduced proteins are 101 to 361 amino acids long. The molecular weight (MW) of the CaGST proteins vary from the lowest 11.62 kDa (CaGSTU29) to the highest of 74.94 kDa (CaGSTF5). However, the predicted pI values ranged from 5 to 9. The average length, MW, and pI of the CaGST proteins were found to be 230 aa, 26.3 kDa and 6.5, respectively (Table 1). Most of the CaGST proteins were predicted to be localized in the cytoplasm, followed by chloroplast, mitochondria, nucleus, extracellular space, and plasma membrane. Secondary structure analysis showed the presence of a higher percentage of α-helix in CaGST proteins as compared with β-sheets (Table S1). The percentage of the extended strand was fluctuating widely among the CaGST proteins, in a range of 10 to 28%. Glycosylation analysis showed that 40 CaGST proteins have a potential N-glycosylation site, whereas CaGSTF5 and CaGHR1 possess the highest number of predicted glycosylation site with seven and five, respectively (Table S2).

**Table 1:** List of identified *GST* genes in pepper along with their detailed genomic and proteomic information.

Sl no	Gene Name	Locus ID	CDS coordinate(5′-3′)	Strand	Gene (bp)	Protein (aa)	MW (kDa)	pI	Localization
1	*CaGSTU1*	Capana00g001895	438859152–438859976	+	825	229	26.25	6.14	Cy^a,b^
2	*CaGSTU2*	Capana00g002164	455860084–455861271	+	1188	225	26.00	5.53	Cy^a,b^
3	*CaGSTU3*	Capana00g003105	524690584–524692284	−	1701	203	23.67	8.46	Cy^a,b^
4	*CaGSTU4*	Capana00g003106	524702849–524704044	−	1196	219	25.56	6.60	Cy^a,b^
5	*CaGSTU5*	Capana00g004478	647297751–647300086	−	2336	221	25.12	5.21	Cy^a^, Cp^b^
6	*CaGSTU6*	Capana00g004596	657125404–657128170	+	2767	222	25.73	5.59	Cy^a^, Cy^b^
7	*CaGSTU7*	Capana00g004598	657374139–657375389	−	1251	220	25.34	5.75	Cy^a,b^
8	*CaGSTU8*	Capana00g004621	659428697–659429026	+	330	109	12.84	6.97	Cy^a,b^, Mt^a^, Cp^a^
9	*CaGSTU9*	Capana00g004665	665401358–665402749	−	1392	224	25.87	5.57	Cy^a^, Cp^b^
10	*CaGSTU10*	Capana01g002551	166700570–166702635	+	2066	221	25.08	5.08	Cy^a^, Nu^b^
11	*CaGSTU11*	Capana01g003292	222778803–222779584	−	782	225	25.63	6.33	Cy^a,b^
12	*CaGSTU12*	Capana01g003296	222806252–222809195	−	2944	222	25.91	6.00	Cy^a,b^
13	*CaGSTU13*	Capana02g000947	103858121–103858901	−	781	153	17.40	5.57	Cy^a,b^
14	*CaGSTU14*	Capana02g000948	103899028–103899345	−	318	105	11.98	9.13	Cy^a^, Cp^a^, Nu^b^
15	*CaGSTU15*	Capana02g000950	104101699–104102578	+	880	225	26.05	5.36	Cy^a^, Cp^b^
16	*CaGSTU16*	Capana02g000952	104250329–104251764	+	1436	224	25.67	5.40	Cy^a,b^
17	*CaGSTU17*	Capana03g000768	11322594–11323373	−	780	212	24.43	5.44	Cy^a^, Cp^b^
18	*CaGSTU18*	Capana03g000769	11362575–11363976	−	1402	233	27.12	5.29	Cy^a,b^
19	*CaGSTU19*	Capana03g004402	253298052–253300358	+	2307	222	25.91	5.00	Cy^a^
20	*CaGSTU20*	Capana03g004562	257827721–257828950	−	1230	225	25.75	6.10	Cy^a,b^
21	*CaGSTU21*	Capana03g004565	258031244–258032469	+	1226	225	26.05	6.25	Cy^a,b^
22	*CaGSTU22*	Capana03g004566	258056794–258057997	+	1204	225	25.93	7.02	Cy^a,b^
23	*CaGSTU23*	Capana06g002861	210286274–210287439	−	1166	220	25.36	5.53	Cy^a,b^
24	*CaGSTU24*	Capana07g002003	210859245–210865929	+	6685	220	25.49	5.89	Cy^a,b^
25	*CaGSTU25*	Capana07g002004	210867066–210867991	+	926	222	25.60	5.79	Cy^a,b^
26	*CaGSTU26*	Capana07g002005	210902595–210903564	+	970	175	20.42	6.84	Cy^a,b^, Cp^a^
27	*CaGSTU27*	Capana07g002006	210941269–210948401	+	7133	282	32.96	8.35	Cy^a,b^
28	*CaGSTU28*	Capana07g002007	210981175–210982949	+	630	209	24.34	8.08	Cy^a,b^
29	*CaGSTU29*	Capana07g002008	211057582–211057887	+	306	101	11.62	5.14	Cy^a,b^
30	*CaGSTU30*	Capana07g002009	211066612–211068955	+	2344	238	27.61	6.25	Cy^a,b^
31	*CaGSTU31*	Capana07g002010	211152285–211153567	+	1223	220	25.54	5.78	Cy^a,b^
32	*CaGSTU32*	Capana07g002011	211155192–211156505	−	1314	219	25.32	6.84	Cy^a,b^
33	*CaGSTU33*	Capana07g002012	211160558–211161672	−	1115	203	23.75	7.00	Cy^a,b^
34	*CaGSTU34*	Capana08g001515	132057487–132058584	+	1098	226	25.76	5.38	Cy^a,b^
35	*CaGSTU35*	Capana08g001518	132082843–132083882	+	1040	202	22.88	5.36	Cy^a^, Cp^a^
36	*CaGSTU36*	Capana08g001520	132086808–132087750	+	943	181	20.61	5.11	Cy^a,b^, Cp^a^
37	*CaGSTU37*	Capana09g001740	199536703–199537891	+	1189	223	25.30	5.30	Cy^a^, Cp^b^
38	*CaGSTU38*	Capana09g001741	200130618–200131846	+	1229	220	25.04	5.48	Cy^a^, Cp^b^
39	*CaGSTU39*	Capana09g001742	200167723–200169649	+	1927	224	26.36	6.77	Cy^a^, Nu^b^
40	*CaGSTU40*	Capana09g001760	202017261–202018643	+	1383	217	25.05	5.28	Cy^a,b^
41	*CaGSTU41*	Capana09g001761	202086834–202088344	+	1511	220	24.85	5.41	Cy^a^, Cp^b^
42	*CaGSTU42*	Capana09g001762	202236152–202237088	+	937	220	25.45	5.12	Cy^a^, Cp^b^
43	*CaGSTU43*	Capana09g001763	202238222–202239079	+	858	154	17.67	5.12	Cy^a^, Mt^a^
44	*CaGSTU44*	Capana09g001764	202383049–202384214	+	1166	220	25.38	5.53	Cy^a,b^
45	*CaGSTU45*	Capana09g001858	213200912–213201949	+	1038	217	25.28	5.38	Cy^a^, Cp^b^
46	*CaGSTU46*	Capana09g001859	213228499–213229454	−	956	219	24.86	5.87	Cy^a,b^
47	*CaGSTU47*	Capana09g001860	213238117–213239430	−	1314	221	25.19	5.23	Cy^a^, Nu^b^
48	*CaGSTU48*	Capana09g001861	213240773–213241709	+	937	224	25.76	5.58	Cy^a^, Cp^b^
49	*CaGSTU49*	Capana09g001862	213303661–213304284	+	624	143	16.62	4.84	Cy^a^, Mt^a^
50	*CaGSTU50*	Capana09g002045	226590245–226590574	+	330	109	13.06	8.78	Cy^a,b^, Mt^a^
51	*CaGSTU51*	Capana11g001524	178109400–178111728	+	2329	231	25.76	6.18	Cy^a^, Cp^a,b^
52	*CaGSTU52*	Capana11g001525	178112748–178114176	+	1429	215	23.85	8.88	Cy^a^, Cp^a,b^
53	*CaGSTU53*	Capana11g001528	178187645–178188768	+	1124	220	24.84	6.75	Cy^a^, Cp^a,b^
54	*CaGSTU54*	Capana11g001532	178455711–178456697	+	987	229	26.20	6.76	Cy^a^, Cp^b^
55	*CaGSTU55*	Capana11g001533	178457971–178458582	+	612	203	23.42	6.31	Pm^a^, Ec^b^
56	*CaGSTU56*	Capana11g001535	178483866–178484900	+	1035	229	26.01	5.57	Cy^a^, Cp^b^
57	*CaGSTU57*	Capana11g001536	178558594–178560496	−	1903	229	26.25	5.45	Cy^a^, Cp^b^
58	*CaGSTU58*	Capana11g001537	178588610–178590596	+	1987	229	26.13	5.62	Cy^a^, Cp^b^
59	*CaGSTU59*	Capana12g000354	7186608–7188114	+	1507	219	25.57	5.47	Cy^a^, Nu^b^
60	*CaGSTF1*	Capana02g002285	142882377–142883807	+	1431	228	26.02	6.09	Cy^a^, Cp^b^
61	*CaGSTF2*	Capana03g003600	230428836–230430376	+	1541	213	24.19	5.69	Cy^a^, Cp^b^
62	*CaGSTF3*	Capana06g003058	218203021–218205366	+	2346	213	23.76	6.39	Cy^a^, Cp^b^
63	*CaGSTF4*	Capana06g001819	54683346–54684364	−	1019	251	29.29	8.68	Cy^a,b^, Mt^a^, Nu^a^
64	*CaGSTF5*	Capana12g000609	14792240–14802489	+	10250	654	74.94	7.13	Pm^a^, Cy^b^
65	*CaGSTF6*	Capana12g000612	14873894–14878274	+	4381	252	28.64	5.26	Cy^a^, Ec^a^, Cp^b^
66	*CaGSTT1*	Capana01g000218	3161948–3164992	+	3045	186	21.15	9.74	Mt^a^, Nu^a^, Cy^b^
67	*CaGSTT2*	Capana01g000219	3169497–3173899	+	4403	250	28.34	9.25	Cy^a^, Mt^a^, Nu^a^
68	*CaGSTT3*	Capana01g000221	3200545–3205818	+	5274	241	27.33	9.26	Cy^a,b^, Mt^a^
69	*CaGSTT4*	Capana12g001178	52439179–52442473	−	3295	235	26.46	6.45	Cy^a^, Cp^b^
70	*CaGSTZ1*	Capana01g001395	45340308–45347371	−	7064	283	32.48	8.34	Mt^a^, Cy^a^, Cp^b,c^
71	*CaGSTZ2*	Capana12g000896	32868383–32869334	+	952	107	12.47	5.01	Pm^a^, Cy^b^
72	*CaGSTL1*	Capana05g000544	14609977–14614464	+	4488	314	35.45	8.71	Cp^a,b,c^
73	*CaGSTL2*	Capana09g001864	213395287–213400333	+	5047	239	27.75	5.57	Cy^a,b^
74	*CaGSTL3*	Capana10g001792	183887466–183889739	−	2274	222	25.80	5.24	Cy^a,b^
75	*CaGSTL4*	Capana10g001806	186021678–186025506	−	3829	253	29.10	5.75	Cy^a,b^, Mt^a^
76	*CaEF1Bɣ1*	Capana11g000124	3480968–3485480	−	4513	414	46.83	5.65	Cy^a^, Cp^b^
77	*CaEF1Bɣ2*	Capana08g000330	43923121–43924032	−	912	204	23.47	8.67	Cy^a^, Nu^a^
78	*CaDHAR1*	Capana05g000799	31400365–31405449	+	5085	295	32.75	8.33	Mt^a^, Cp^a,b,c^
79	*CaDHAR2*	Capana05g002401	214455819–214460536	−	4718	212	23.57	5.88	Cy^a,b^
80	*CaTCHQD*	Capana00g004998	686182991–686184172	+	1182	268	31.64	9.06	Mt^a^, Cy^a^
81	*CaMGST1*	Capana02g002296	143033395–143035039	+	1645	144	16.38	9.15	Pm^a^
82	*CaMGST2*	Capana04g000166	2049728–2054459	−	4732	323	36.21	9.02	Cp^a,c^
83	*CaGHR1*	Capana02g001236	116162258–116176687	+	14430	361	41.43	6.80	Mt^a^, Nu^a^, Cp^c^
84	*CaGHR2*	Capana02g000926	102853919–102854627	−	709	123	13.73	4.37	Nu^a^, Cy^a^
85	*CaGHR3*	Capana06g000146	1927452–1929409	+	1958	373	41.82	6.61	Cp^a,c^

Abbreviations- CDS, coding DNA Sequence; MW, Molecular Weight; pI, Isoelectric point; bp, base pair; aa, amino acid; kDa, kilodalton; Cp, Chloroplast; Ec, Extracellular; Cy, Cytoplasm; Mt, Mitochondria; Nu, Nucleus; Pm, Plasma-membrane.

^a^Localization prediction by CELLO v.2.5 (http://cello.life.nctu.edu.tw/).

^b^Localization prediction by pSORT (https://wolfpsort.hgc.jp/).

^c^Chloroplast localization signal confirmed by ChloroP (http://www.cbs.dtu.dk/services/ChloroP/).

### Genomic organization of the pepper *GST* gene family

**Figure 1:**
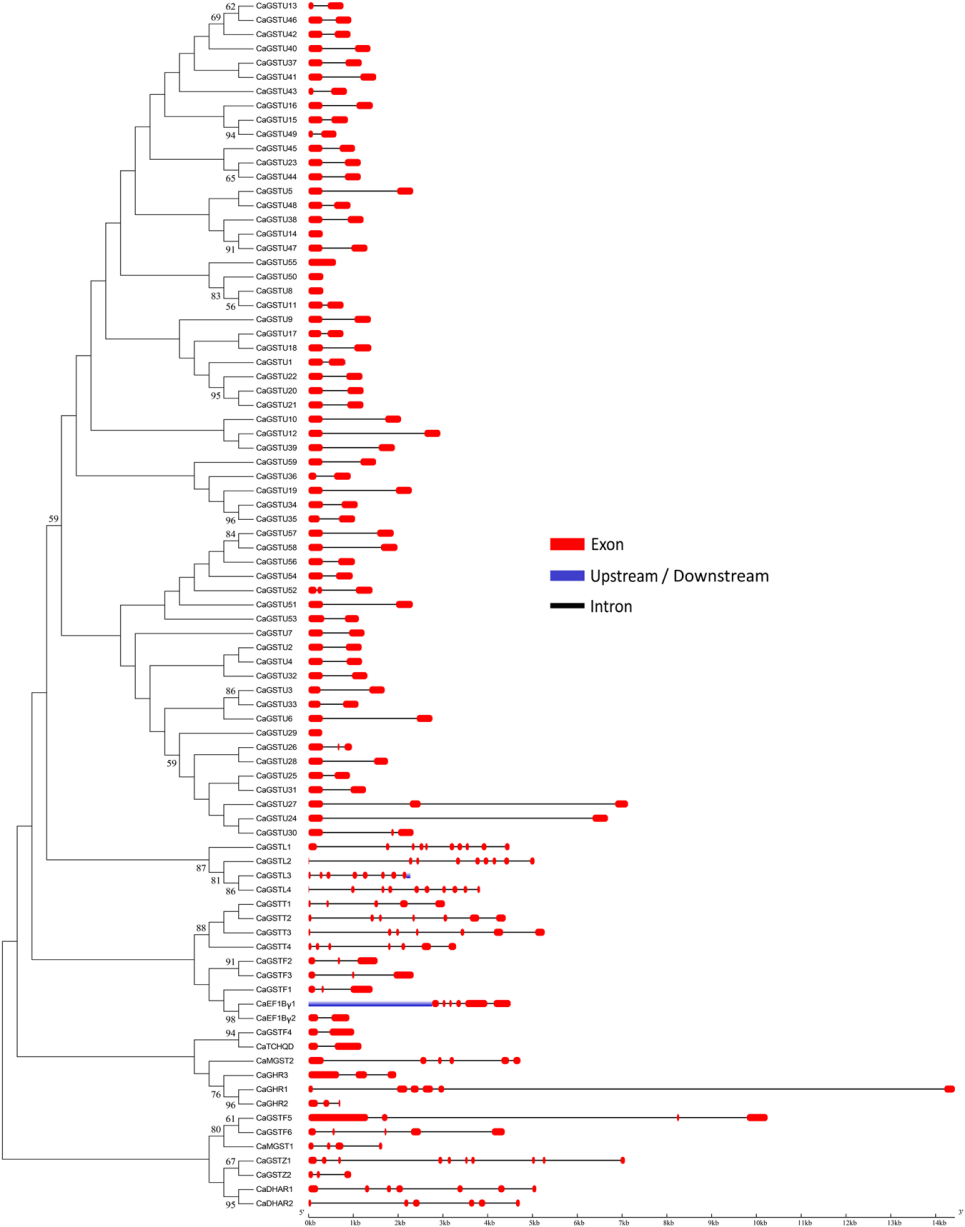
Phylogenetic and Structural analysis of *CaGST* genes. (**A**) Number at each node in the phylogenetic tree represents the bootstrap value higher than 50. The different class of *GST* genes forms separate clades. (**B**) The schematic diagram represents the gene structure of all 85 *CaGST* genes identified in this study. Exons are shown as red boxes; introns are shown as black lines, and the upstream/downstream regions are shown as blue boxes. The relative size of the full transcript, intron, exon, and upstream region could be inferred from the supplied scale in kilobase pair (kb).

All the *CaGST* genes are distributed randomly and unevenly in all the 13 chromosomes of pepper (Fig. S1). Chromosome 9 is the most densely populated with fifteen genes (17.6%), followed by ten genes each in chromosome 7 and 0, then nine genes each in chromosome 2 and 11 (Fig. S1). A total of sixteen gene clusters were observed on 12 different chromosomes. Among these clusters, eleven were formed among tau members, one each was for the theta and GHR cluster. Twenty-four sets of CaGST proteins appeared to be ≥80% similar, that indicates the possible gene duplication events among these genes (Table S3). A maximum of nine duplicated *GST* genes were located in chromosome 7, followed by six in chromosome 9 and five in chromosome 11. Out of the twenty-four gene pairs, twelve pairs were found as tandemly duplicated, whereas rest twelve pairs appeared as segmentally duplicated. Furthermore, the substitution rate of non-synonymous (d_N_) and synonymous (d_S_) ratios were calculated to investigate the selective constraints on the duplicated *CaGST* gene pairs, where values >1, <1, and equal to 1 implies positive selection, purifying selection, and neutral selection, respectively. All the identified duplicated *CaGST* gene pairs showed the d_N_/d_S_ value less than 0.7, signifying the influence of purifying selection behind the evolution of these gene pairs. Moreover, the estimated divergence time of the duplicated gene pairs varies from 1.18 Mya to 17.84 Mya (Table S3). Identification of the exon-intron organization of 85 *CaGST* genes revealed a group-specific exon/intron patterns within each *GST* class. A similar type of exon/intron number and length were notable in the phylogenetically related members (Fig. 1). The tau-class *CaGST*s typically contained one/two exons, while phi *GST* genes contained two to five exons in its gene structure (Fig. 1). *CaGSTZ*1, *CaGSTL*1, and *CaGSTL*4 contained the maximum number of nine introns and ten exons, followed by 9 exons in *CaGSTL*2, 8 exons in *CaGSTL*3, 7 exons in *CaGSTT*2, *CaGSTT*3, *CaGSTT*4 and *CaDHAR1* (Fig. 1). An interesting pattern of intron distribution was observed among the putative paralogous members. Most of the paralogs showed same number and similar sized intron such as gene cluster of *CaGSTU*13, 46, 42 and *CaGSTU*23, 44, 45; while some of them showed intron gain/loss phenomenon as compared with their paralogs (*CaGSTU*26 and 30, *CaGSTL*3 and 4, *CaEF1Bγ*1 and 2, *CaGHR*1 and 2, and *CaDHAR*1 and 2). Few of them showed partial deletion of exon such as *CaGSTU*54 and 56 as compared with their two paralogs- *CaGSTU*57 and 58 (Fig. S2A), while some others showed exon duplication such as *CaGSTU*14 and *CaGSTU*47 (Fig. S2B).

Domain architecture analysis showed that 60 out of 85 CaGST proteins contained two conserved GST domains, namely, N-terminal and C-terminal (Fig. S3). Only one N-terminal domain present in 22 CaGSTs, whereas only C-terminal domain present in 3 CaGST proteins (CaGSTU13, CaGSTU43, and CaGHR2). Additional distinct EF1Bγ domain (PF00736) was found to be present in the CaEF1Bγ1 and CaEF1Bγ2 proteins, while the MAPEG domain (PF01124) was present only in CaMGST1 protein. Analysis of conserved motifs of CaGST proteins identified ten distinct motifs (Table S4, and Fig. S4). Among them, four motifs are located in the N-terminal GST domain, while the rest six reside in the C-terminal GST domain of the proteins. Motif3 is found to be present in the N-terminal of 65 proteins; followed by motif 1, motif 4, motif 2, motif 7, and motif 5 with 58, 57, 55, 53 and 51 sites, respectively. Interestingly, lambda class specific pattern was shown by motif 9, while motif 10 was present only in four tau class members (CaGSTU1, CaGSTU20, CaGSTU21, and CaGSTU22). Among others, motif 6 and 8 were present in 44 and 16 CaGSTs, respectively. The lowest number of one motif (either motif 4 or 8) is present in CaGSTZ2, CaGSTL2, CaEF1Bγ2, CaMGST1, CaGHR2, and CaGHR3.

### Phylogenetic analysis of GST proteins

**Figure 2:**
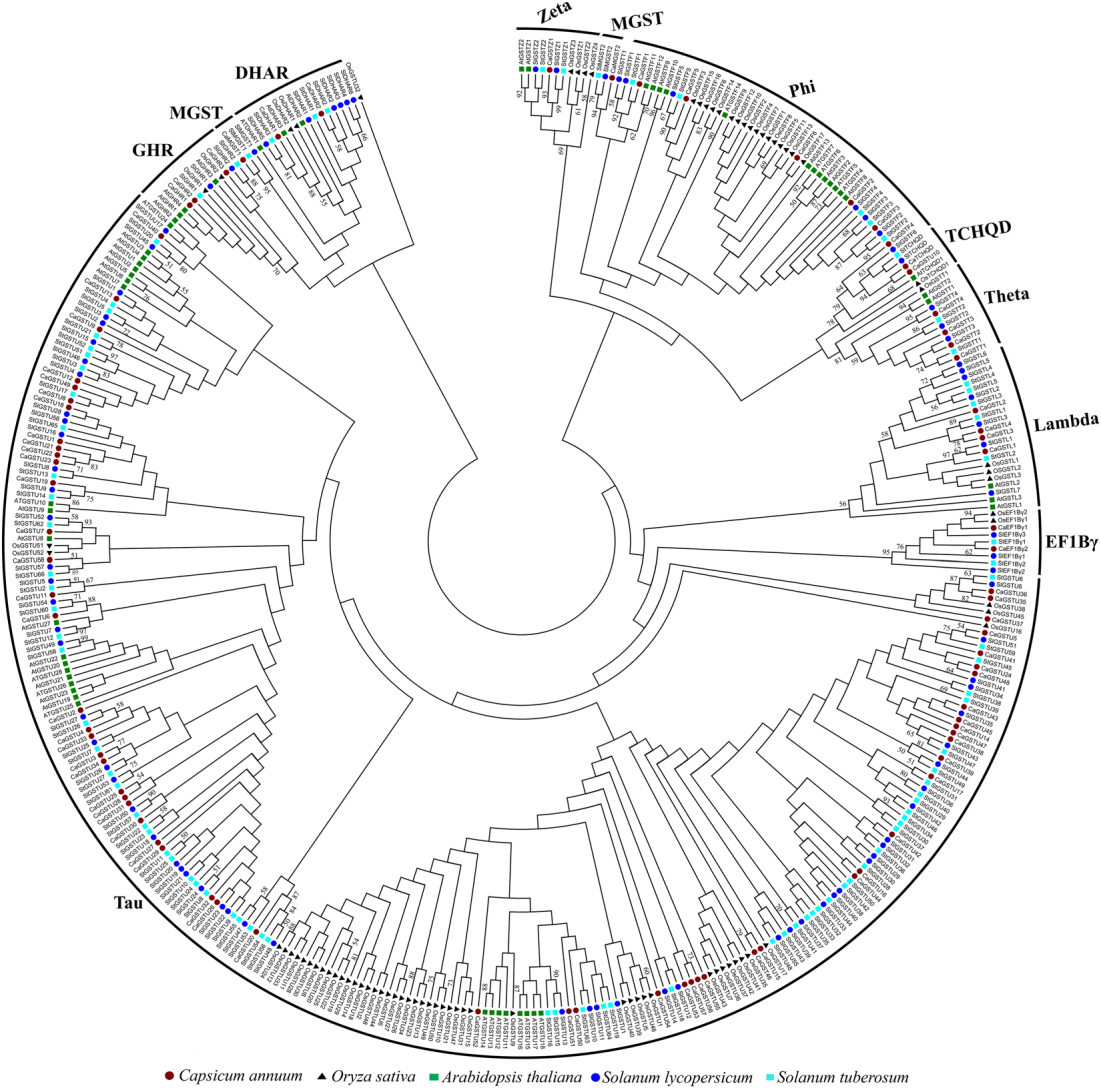
Phylogenetic analysis of GST proteins. GST proteins from five species- pepper, tomato, potato, *Arabidopsis*, and rice were used to construct an unrooted phylogenetic tree using MEGA Maximum-likelihood method with 1000 bootstraps. GST members from tau, lambda, zeta, DHAR, theta, GHR, TCHQD, phi, MGST, EF1Bγ classes were marked with red, green, cyan, blue, violet, gray, coral, yellow, pink, royal blue; respectively. Members of each class formed different clades with few exceptions.

The evolutionary relationship of GST family members was predicted by comparing them to different plant species. A total number of 401 full length GST protein sequences from five different plant species- pepper, *Arabidopsis*, rice, tomato, and potato were aligned to create an unrooted maximum likelihood phylogenetic tree (Fig. 2). All these CaGST proteins were found to be closely associated with an individual class of GSTs. Most classes formed a monophyletic group with very few exceptions of OsGSTU32, CaGSTU10, and SlGSTT1 (Fig. 2). The tree clearly suggested that the largest and most abundant class of GST is tau (brown circle) with 59, 52, 28, 57 and 66 members in pepper, rice, *Arabidopsis*, tomato, and potato; respectively. All these tau members are distributed in 6 different small clades under a large superclade, which indicates the presence of internal variation among these tau members. Similarly, the second largest clade is formed by the phi members with a green rectangle. The clustering of same classes of GST from five plant species demonstrates that the presence of all these GST individual classes during the divergence of plants followed a species-specific gene duplication. The major divergences that divided the family into 10 individual classes might occur in the common ancestor of all the investigated species.

### Molecular evolution of *Capsicum* and tomato GST family

**Figure 3:**
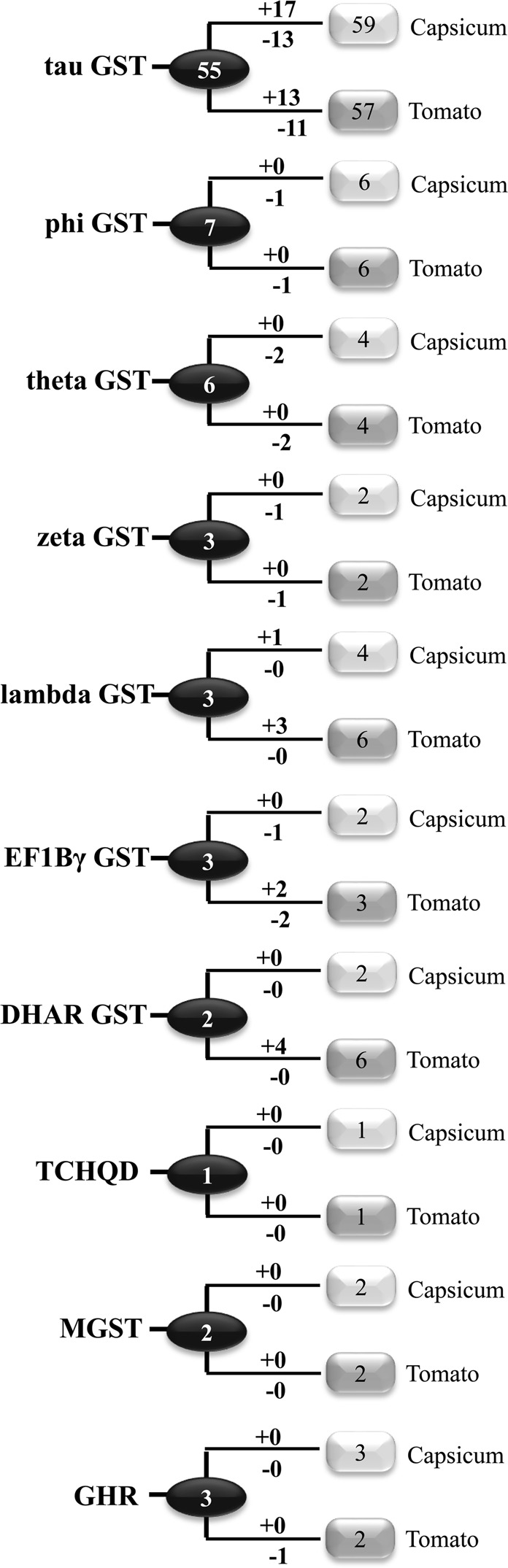
Copy number variation between *Capsicum* and tomato *GST* genes. The numbers presented inside the circles and rectangles represent the numbers of *GST* genes as common ancestral and species-specific, respectively. Numbers on the branches with plus and minus symbols represent the numbers of gene gains and losses, respectively throughout the evolution between these two species. The light gray boxes represent *Capsicum*, while dark gray boxes symbolize tomato.

To investigate the lineage-specific expansion of *GST* genes in *Capsicum* and tomato genome, a phylogenetic analysis was performed using the GST protein sequences from *Capsicum* and tomato. A total of 85 *Capsicum* and 90 tomato GSTs fell into ten distinct classes. The divergence point between *Capsicum* and tomato were labeled with circles on certain specific nodes of the phylogenetic tree that represents the most recent common ancestral (MRCA) genes before the split (Figs S5–S10). Most of the MRCA have a member from both the species, while some of them have representation from either *Capsicum* or tomato, indicating a subsequent loss in tomato and *Capsicum* genome, respectively. There were at least 55 MRCA for tau *GST* genes between *Capsicum* and tomato out of a total of 59 and 57 members, respectively (Fig. 3). After the split, *Capsicum* gained 17 genes and lost 13 genes, resulting in the 59 tau *GST* genes; while tomato gained 13 genes and lost 11 genes, resulting in 57 tau genes. For phi GST, there are at least 7 MRCA between *Capsicum* and tomato (Fig. 3). After the split, both of them lost one member without any gain, resulting 6 phi members in each species (Fig. 3). Similarly, two genes have been lost from 6 MRCA of theta GST to result in 4 existing members in each species.

For zeta, lambda, EF1Bγ, and GHR GSTs, there are at least three ancestral genes in the MRCA analysis of *Capsicum* and tomato (Fig. 3). After the split, both *Capsicum* and tomato lost one zeta member without any gain; no loss with one gain for *Capsicum* and three gain for tomato lambda GST; one loss without gain for *Capsicum* and two loss with two gain for tomato EF1Bγ GST; and no loss/gain for *Capsicum* and one loss without any gain for tomato GHR *GST* genes. For DHAR GST, the MRCA of *Capsicum* and tomato had at least two ancestral members (Fig. 3). After the split, there was no gain/loss event in *Capsicum*, while four genes have been gained by tomato. For TCHQD GST and MGST classes, there were no identifiable gene gain or loss events after the split of these two species (Fig. 3).

### Expression analysis of *CaGST* transcripts in different tissues

**Figure 4:**
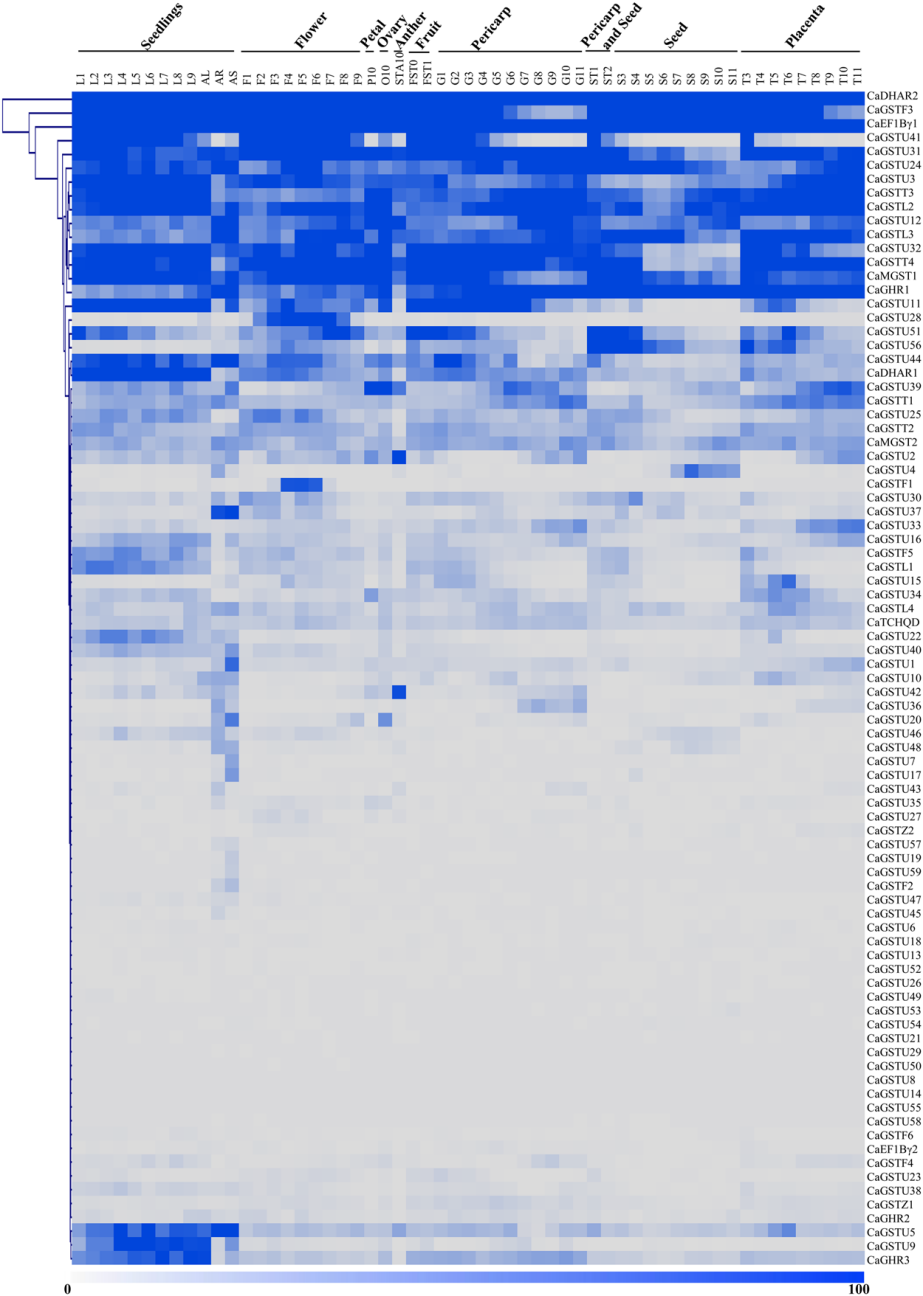
Expression profiling of *CaGST* genes at different anatomical tissues. Expression of all the identified *CaGST* transcripts was analyzed in 57 developmentally diverse tissues using the RNA-seq data. All these tissues could be categorized into nine major stages, such as seedlings, flower, petal, ovary, anther, fruit, pericarp, seed, and placenta. Heatmap with hierarchical clustering was performed using the expression values in MeV software package with Manhattan correlation. The highest level of expression is represented by dark blue (100%), while the low level is presented as white (0%). Thus, the intensity of the color in the heatmap is directly proportioned to the transcript abundance of each member.

To investigate the putative roles of *CaGST* genes in *C. annuum* growth and development, expression of all the identified *CaGST* genes were analyzed in the 57 different tissues and organs based on the RNA-seq data. All these tissues could be broadly represented into nine major organs/stages: seedlings, flower, petal, ovary, anther, fruit, pericarp, seed, and placenta. The analysis revealed a differential pattern of expression for different *CaGST* transcripts depending on the type of tissues and organs (Fig. 4). Based on the differential expression patterns, all these genes could be classified into three groups: a) Some *CaGST*s showed extremely low levels of expression in almost all tissues and organs, b) Some *CaGST*s exhibited low to medium levels of expression among different organs/tissues, and c) Some were highly expressive across all the tissues of the entire life cycle. Among all, *CaDHAR*2 and *CaEF1B*γ1 showed the maximum level of expression in all the tissues, while other members of this clade such as *CaGSTF*3, *CaGSTU*31, *CaGSTU*24, *CaGSTU*3, *CaGSTT*3, *CaGSTL*2, *CaGSTU*12, *CaGSTL*3, *CaGSTU*32, *CaGSTT*4, *CaMGST*1, and *CaGHR*1 exhibited a high level of expression in most of the tissues (Fig. 4). Notably, two clusters of *GST* genes from *CaGSTU*51 to *CaDHAR*1 and from *CaGSTU*5 to *CaGHR*3 (Fig. 4) maintained a medium to high levels of expression in all the analyzed tissues. Interestingly, some of the members showed a highly tissue-specific expression, such as *CaGSTU*41 showed a low level of expression in pericarp, seed and placental otherwise the level is high in other tissues; *CaGSTU*28 and *CaGSTF*1 possessed only flower-specific expression; expression of *CaGSTU*56 raised significantly in seed and placenta; and the cluster of *CaGSTU*5 to *CaGHR*3 showed a high level of seedling specific expression (Fig. 4). Thus, different *GST* transcripts might have different developmental and tissue-specific regulation to maintain their specific localized or ubiquitous function.

### Transcript analysis of *CaGSTs* in response to various abiotic stresses

**Figure 5:**
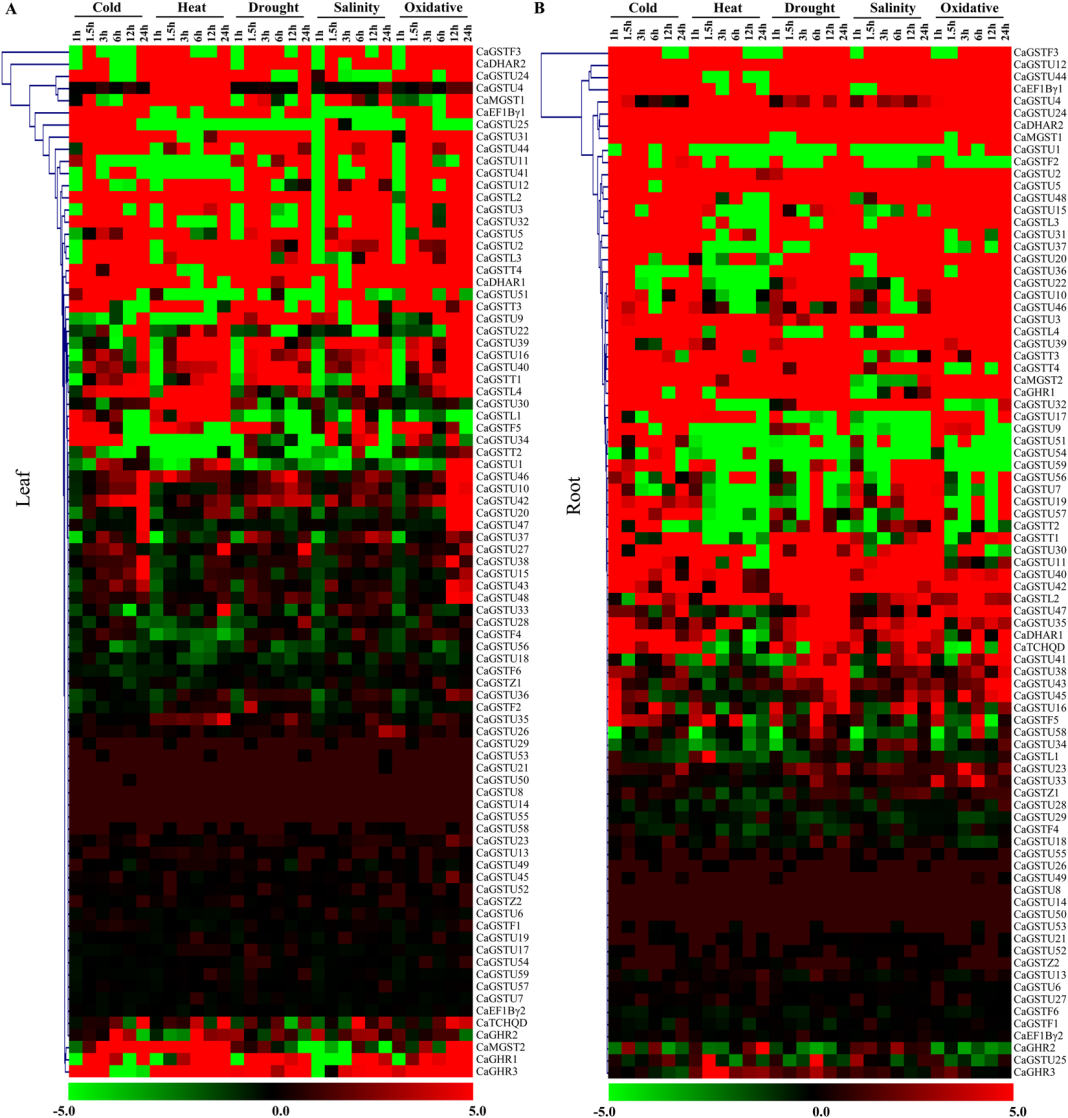
Alteration of *CaGST* transcripts in various abiotic stresses. Expression of all 85 *CaGST* transcripts was analyzed from the leaf (**A**) and root (**B**) samples treated with five major abiotic stresses, such as cold, heat, drought, salinity and oxidative. Relative fold change of transcript abundance in seven different time points of each stress (1 h, 1.5 h, 3 h, 6 h, 12 h, 24 h, and 48 h) was plotted with MeV software package. The color scale, depicted at the down of each heatmap, represents the intensity of alterations where green color indicates downregulation and red indicates upregulation.

To identify the abiotic stress-responsiveness, expression profiling of all *CaGST* transcripts were further analyzed in response to five different abiotic stresses viz. cold, heat, drought, salinity and osmotic using the Illumina RNA-Seq data (Fig. 5). Transcriptome profiling was performed in the leaf and root tissue of hot pepper at six different time points such as 1 h, 1.5 h, 3 h, 6 h, 12 h, and 24 h. Most of the genes showed a different level of upregulation with few downregulation events. As compared with the leaf samples (Fig. 5A), the genes showed more upregulation in the root samples (Fig. 5B) as root is one of the first organs to perceive the adverse conditions. A large cluster of genes (*CaGSTU*55 to *CaEF1B*γ2) showed a minimum alteration in response to all these stresses in both the leaf and root tissues. Two clusters of genes; one from *CaGSTF*3 to *CaMGST*1, and another from *CaGSTU*2 to *CaGSTU*48, showed the maximum upregulation in the root against all these stresses (Fig. 5B), while there is no such unique pattern of upregulation in the leaf (Fig. 5A). Moreover, there are a few stress-specific transcript alteration events in the root (Fig. 5B) such as *CaGSTU*1 showed cold and oxidative stress-specific upregulation; a cluster of *CaGSTU*15 to *CaGSTU*46 showed heat-specific downregulation; and *CaGSTF*2, *CaGSTU*51, and *CaGSTU*54 exhibited cold-specific upregulation. Likewise, a cold specific upregulation for *CaGSTU*25 and *CaGSTU*34; and drought and salinity specific upregulation for *CaGSTU*11 and *CaGSTU*44 was observed in the leaf samples (Fig. 5A).

### Stress-responsive alteration of total GST activity

**Figure 6:**
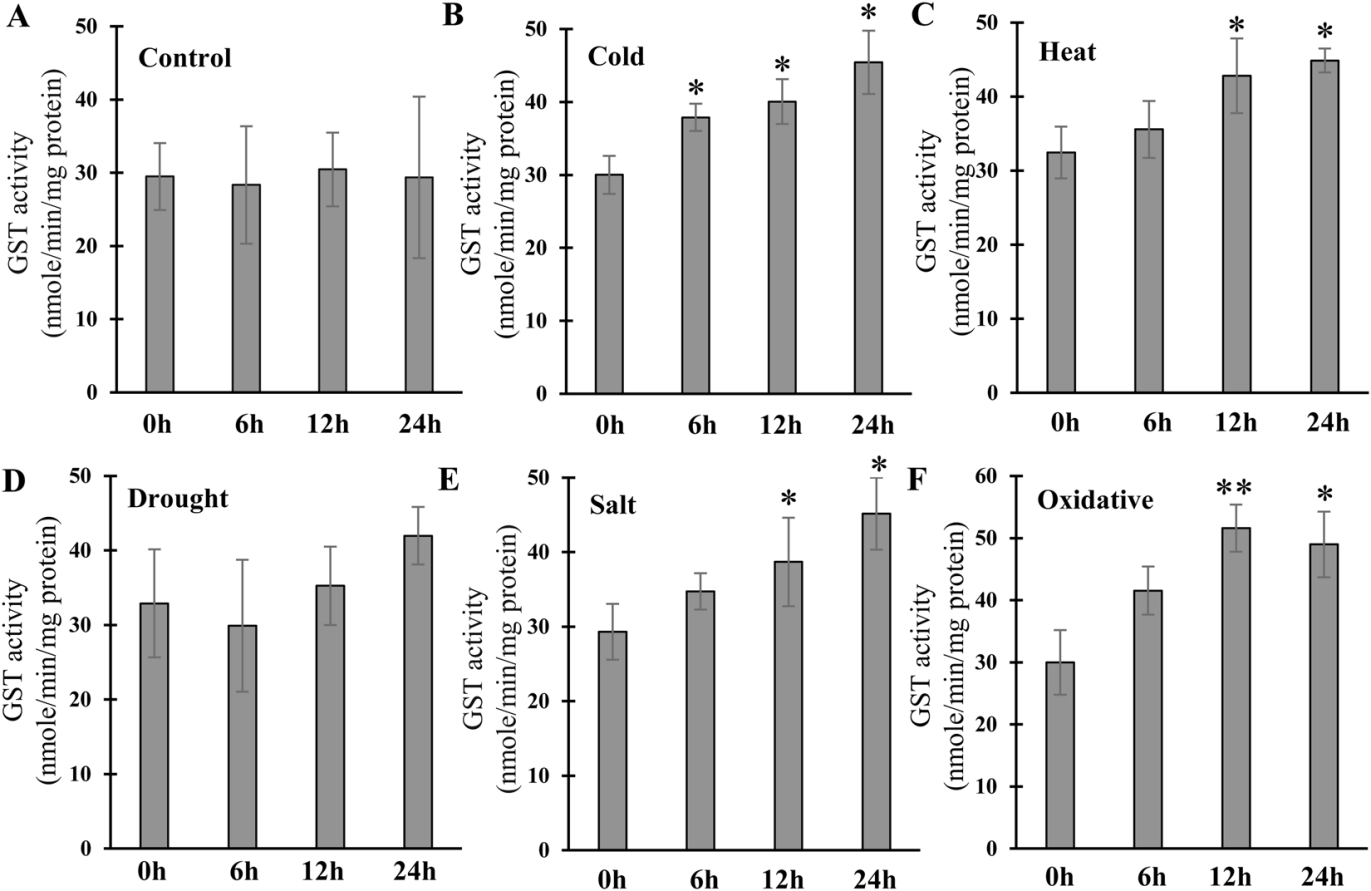
Measurement of total GST activity in response to various abiotic stresses. Total GST enzyme activity was measured in response to various abiotic stresses such as cold, heat, drought, salinity, and oxidative at four different time points of stress exposure. The activity was represented as nmoles/min/mg protein. All the experiments were repeated thrice and represent as the average ± standard deviation (n = 3). The significance level of the paired student’s two-tailed t-test is represented as * and ** with a p-value less than 0.05 and 0.01; respectively.

In order to validate the abiotic stress specific up-regulation of the majority of *CaGST* transcripts, the total GST activity was measured in response to the same abiotic stress conditions- cold, heat, drought, salinity, and oxidative; and compared with the respective untreated control conditions (Fig. 6). As shown in Fig. 6, a strong positive induction of GST activity was observed in response to all these stress treatments. GST enzyme activity enhanced significantly in response to heat, cold, salt, and oxidative stresses as compared with their untreated (0 h) sample (Fig. 6). A gradual enhancement of GST activity with time was observed for heat, cold and salt stresses, while total GST activity under oxidative stress reached a maximum level within 12 h of stress imposement, and maintained the level until 24 h observation period (Fig. 6F). Among all these stresses, drought showed minimum induction with a slow rate over the time of 24 h (Fig. 6D). However, the level of total GST activity in the untreated control sample remains almost similar within the 24 h experimental period (Fig. 6A).

### Presence of Cis-elements in the *CaGST* promoters

**Figure 7:**
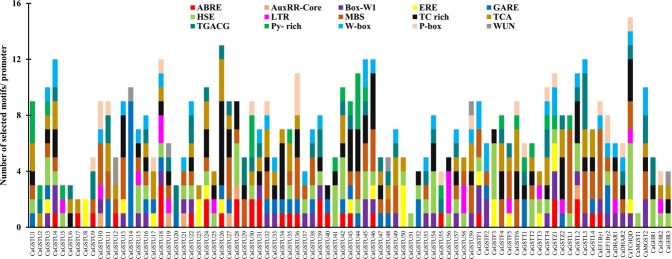
Analysis of the putative promoter of *CaGST* genes. One kb 5′ upstream sequences of all the identified *CaGST* genes were retrieved from the genome database and analyzed through PlantCARE to identify the presence and number of various cis-acting regulatory elements. Different hormone-responsive and stress-related elements were identified and plotted against a bar diagram. The abundance of different regulatory elements on each of the promoter was represented with different colors.

To understand the tissue-specificity or stress-responsive transcriptional regulation of *CaGST* genes, the 1000 bp 5′ upstream region from the transcriptional start site (ATG) was retrieved and scanned through PlantCARE database for the identification of important cis-regulatory elements. The promoters possessed several cis-elements that confer hormone and stress response. We have identified the presence of seven hormone-related such as abscisic acid-responsive (ABRE), auxin-responsive (AUXRR-core), ethylene-responsive (ERE), gibberellin-responsive (GARE and P-box), salicylic acid-responsive elements (TCA-element) and MeJA-responsive element (TGACG-motif); seven defense and stress-responsive elements such as fungal elicitor-responsive (Box-W1, W-Box), heat stress-responsive (HSE), low-temperature-responsive (LTR) elements, MYB binding site involved in drought-inducibility (MBS), stress responsiveness (TC-rich repeats), and wound-responsive element (WUN-motif); and one transcriptional enhancer (pyrimidine-rich motif) (Fig. 7). The upstream region of most *CaGST* members contained at least one hormone-related and one stress-related element. The top four abundant elements involved in the hormone and stress responses are - MBS element with 85 instances, 83 instances of the defense and stress-responsive element (TC-rich repeats), 64 instances of HSE element and 63 instances of TCA element (Table S5). The promoter of *CaTCHQD* contains the highest number of 15 cis-element, followed by the promoter of *CaGSTU*26 with 13 members. Whereas the minimum number of one cis-regulatory element was positioned in the promoter of *CaGSTU*51 and *CaMGST*1, followed by the promoter of *CaGSTU*7 and *CaGSTU*8 with 2 motifs (Fig. 7). Presence of such diverse type of hormone and stress-inducible cis-elements could be directly correlated with the positive alteration of the *CaGST* transcripts (Fig. 5) and total GST activity (Fig. 6) under various abiotic stress conditions.

## Discussion

Hot Pepper (*C. annuum*) is one of the most economically important, nutritious and world-wide grown spicy vegetable^37^. Various environmental stresses including salt, wind, cold, temperature, drought, humidity and osmotic stress cause a serious threat and thus, constrain the total production of pepper^38^. Water deficit notably diminishes the final fruit production in *Capsicum chinense* by interfering with the process of flowering and fruit development^39^. Much focus has been given in recent years to understand the stress responses and adaptation mechanisms of pepper by identifying many of the master regulatory genes^40–42^. GSTs are a promiscuous enzyme family that plays a vital role in the growth and development as well as stress management. A comprehensive genome-wide exploration identified a total of eighty-five *CaGST* gene members (Table 1) with at least one conserved GST domain (Fig. S3). The number of identified *GST* genes are higher in pepper as compared to most other species; but fewer than barley, tomato, potato and soybean^14,15,22–25,28–33,36,43,44^. Among the ten identified classes of CaGST, tau is the most abundant class, followed by phi (Table 1). The almost similar pattern of tau and phi dominance was observed in other plant species; and thus, termed as plant-specific GSTs^15,45^. In spite of the ubiquitous abundance of the tau class in tracheophytes (25–62 copies), they are completely missing in *Physcomitrella patens* and green algae^35^. The possible reason behind the tau and phi class-specific expansion might be their substantial functional influence on the xenobiotics metabolism and stress tolerance against both pathogens and environmental factors^35,46–48^.

The expansion of a gene family occurs mainly through three evolutionary mechanisms such as tandem duplication, segmental duplication, and transposition^41^. Due to the polyploidy nature of most plants, segmental duplication occurs frequently as compared with the other two means. The members of *CaGST* gene family arises largely due to several rounds of tandem and segmental gene duplications. Among the 59 tau GSTs in *Capsicum*, sixteen (27%) were created by segmental duplication and fourteen (23%) by the tandem duplication event, indicating that segmental duplication has the major contribution for the rapid expansion of the tau GSTs in *Capsicum* (Table S3). Apart from that, gene clustering also played an important role in the family expansion where 52 out of 59 *CaGSTU*s (88%) were presented in the eleven clusters on eight chromosomes (0, 1, 2, 3, 7, 8, 9 and 11). Similarly, 3 out of 4 theta GST (75%), 3 out of 3 DHAR GST (100%), 2 out of 4 lambda GST (50%), 2 out of 6 phi GST (33%) formed one cluster each (Fig. S1). This indicates a major contribution of tandem clustering towards the expansion of gene members in each class. Interestingly, different GST proteins from the same genomic cluster showed distinct variation in their enzymatic activity, catalytic efficiency, substrate affinity, and specificities^34,35^. The reasons behind these extensive tandem duplication event in *GST* gene family with the diverse kinetic property are still unknown.

Most *CaGST* genes shared a similar exon-intron structure within the same phylogenetic group (Fig. 1), indicating that the evolution of GST domains may be closely related to the diversification of gene structure. Gene structure analysis showed variation in the presence of exon number in various *CaGST* genes. The number of exons varied from one to a maximum of ten in *CaGST* genes, where the majority of them were single exonic (Fig. 1). Likewise *CaGST*s, the presence of nine introns was reported previously for tomato and potato *GST* genes^28,36^, while a maximum number of 16 and 14 were reported in *Vigna radiate* and *Chinese cabbage*, respectively^49,50^. Previous reports suggested that introns influence and enhance the expression of a gene in a eukaryotic organism, which has been experimentally validated with heterologous gene expression in *Arabidopsis thaliana*^51,52^. The clustering of most intronless or intron-containing genes into the same group (Fig. 1), suggested that this may be a unique feature of the evolution of pepper *GST* gene family. Due to the less selection pressure, intronic sequences possess a higher rate of gain and loss as compared with exonic sequences^53^. In two of the five segmentally duplicated rice gene pairs showed intron gain event^53^. An interesting study of 612 pairs of paralogs from seven representative gene families and 300 pairs of orthologs from different species, concluded that orthologs are more conserved with significantly fewer structural changes as compared with paralogs of similar evolutionary time^54^.

Phylogenetic analysis revealed that *CaGSTs* were closely allied to the same class of GSTs of four other plant species- tomato, potato, *Arabidopsis*, and rice (Fig. 2). This suggested that the evolution and divergence of each GST classes have happened before the split of monocot and dicot. However, CaGST members are more closely related to the tomato and potato as compared with *Arabidopsis* and rice counterparts, reflecting the fact that pepper, tomato, and potato belong to the same superfamily of eudicots and diverged more recently from a common ancestor^37^. Orthologous gene-based phylogenetic analysis of grape, papaya, pepper, tomato, potato, and *Arabidopsis* genome concluded that pepper has been separated from tomato and potato ∼36 Mya ago^55^. Thus, pepper GSTs were compared with that of tomato to elucidate the lineage-specific expansion and genome diversity among these two species (Fig. 3). Thirteen clades contained only CaGST and seven clades contained only tomato GST (Figs S3–S10), indicating that gene loss might have occurred in these clades. The number of clades indicated that there were at least 85 ancestral *GST* genes before the *Capsicum*-tomato split (Fig. 3).

Extensive expression analysis revealed the developmental stage and tissue specific transcript alteration of *CaGST* genes (Fig. 4). Similarly, nine genes were expressed ubiquitously, ten showed root specificity and two expressed in leaves out of 37 tau *GST* of *Sorghum bicolor*^56^. Six sunflower *GST* genes were mainly expressed in leaves, four in seeds and two each in flowers and roots out of a total of 14 identified members^32^. In addition to the developmental alteration, expression of *GST* genes also showed significant variation in response to adverse environmental conditions (Fig. 5). Plants exposure with various abiotic stresses such as cold, heat, drought and salt resulted in the enhancement ROS level and thus, caused oxidative stress^57^. Tau GSTs could protect the cell by enhancing the detoxification of herbicides such as atrazine, metolachlor, flurodifen, and thiocarbamates^58^ and maintaining higher GST activity under salinity and oxidative stress^19^. *JrGSTT*1 enhanced chilling stress tolerance of *Juglans regia* by protecting oxidative enzymes, scavenging ROS, and elevating the expression of several stress-related genes^59^. Upregulation of most of the *SlGST* transcripts in response to multiple abiotic stresses could be directly harmonized with the enhancement of total GST activity under similar conditions^36^. Similarly, upregulation of 6 *HaGST*s (*HaGSTU*1, *U*2, *U*5, *U*6, *F*2, and *Z*1) expression showed a significant positive correlation with the changes in their respective GST activity^32^. The present study also found the positive relationship between the *GST* transcript upregulation and enzyme activity in pepper against four abiotic stresses and oxidative injury (Fig. 6). Heterologous expression of one of the sweet orange tau *GST* or *JrGSTT*1 in tobacco enhanced tolerance against herbicide, salinity, chilling and drought stresses^56,59^. Several stress-responsive motifs were identified in the putative promoter region of *CaGST* genes (Fig. 7). Cis-acting elements played important to control/regulate the expression of genes and thus, modulating plant response against stress and developmental changes^60^. Two commonly present abiotic stress‐inducible cis‐acting elements, dehydration‐responsive element (DRE) and ABRE are found to be interdependent in the ABA‐responsive expression of *Atrd29*A gene^60,61^. Similarly, the presence of two putative low-temperature responsive cis-elements in the 5′-proximal region of *BN115* gene was found to be indispensable for its cold-induced expression in *Brassica napus*^61^. The highly abiotic stress responsive genes of the present study such as *CaGSTF*3, *CaGSTL*2, *CaGSTL*3, *CaEF1Bγ*1, and *CaGSTU*44, showed the presence of a variable amount of HSE, MBS, TC-rich repeat, and LTR motifs (Figs 6, 7). All these common motifs might work synergistically depending on the type of stress to induce the expression of a maximum number of stress-responsive genes.

Taken together, our results provide a comprehensive analysis of the *GST* gene family in pepper. Sequence and phylogenetic analysis of GST from five different plant species revealed the evolutionary conservation of each class of GST proteins. A close relationship between the expression and activity of GST with plant stress tolerance established GST as a major stress biomarker for the plant.

## Materials and Methods

### Sequence retrieval, analysis, and annotation

To retrieve all GST members in pepper, previously reported rice and *Arabidopsis* GST protein sequences from each class was taken as a query in the blastp search with the default parameters (e-value10^−10^) against the Pepper Genome Protein Database (release 2.0) (http://peppersequence.genomics.cn; http://public.genomics.org.cn/BGI/pepper/) and Sol Genomics Network (SGN) (https://www.solgenomics.net/). Sequences were then analyzed through the NCBI conserved domain database (https://www.ncbi.nlm.nih.gov/Structure/cdd/wrpsb.cgi) to identify the individual class of each identified members. Detailed information about the locus name, CDS coordinate (5′-3′), length of the transcript and peptide were collected from the Pepper Genome Database (http://pepperhub.hzau.edu.cn/pegnm/) and Sol Genomics Network (SGN). The ProtParam tool (http://www.expasy.org/tools/protparam.html) was used to calculate various physiochemical properties like molecular weight and theoretical isoelectric point (pI) of the identified proteins. The secondary structure of GST proteins was predicted using the SOPMA (Self-Optimized Prediction Method with Alignment, https://npsa-prabi.ibcp.fr/cgi-bin/npsa_automat.pl?page=/NPSA/npsa_sopma.html). Moreover, N-glycosylation sites were identified using NetNGlyc 1.0 server (http://www.cbs.dtu.dk/services/NetNGlyc/). Furthermore, subcellular localization was predicted using the CELLO version 2.5 (http://cello.life.nctu.edu.tw/), pSORT (http://www.genscript.com/wolf-psort.html) and ChloroP server (http://www.cbs.dtu.dk/services/ChloroP/). Pfam (http://pfam.xfam.org/) was used to assess the conserved GST_N and GST_C domains in all the identified members. Domains were graphically depicted using the software ‘Illustrator for Biological Sequences’ (version 1.0.2). The conserved motifs of CaGST proteins were identified using the online MEME server (http://meme-suite.org/tools/meme).

### Chromosome localization, gene structure and duplications

The physical location of *CaGST* genes was collected from the Pepper Genome Database and the positions of these *CaGST* genes were plotted to thirteen *C. annuum* chromosomes. Exon-intron structure of *CaGST* genes was obtained by gene structure display server (http://gsds.cbi.pku.edu.cn/) using the corresponding genomic and CDS sequence. Duplication events were predicted by blastp search (e-value10^−10^) with ≥80% sequence identity in the Pepper Genome Database^62^. Two or more homologous genes within 100 Kb region on the same chromosome were considered as tandemly duplicated (TD)^62^, while those located beyond 100 kb region were designated as segmental duplication (SD). Synonymous rate (d_S_), non-synonymous rate (d_N_), and evolutionary constraint (d_N_/d_S_) between the duplicated *CaGST* gene pairs were analyzed using the PAL2NAL online tool (http://www.bork.embl.de/pal2nal/). Divergence time (T) of each duplicated gene pairs was calculated using the formula: (T = d_S_/2λ), where λ is considered as a fixed rate of 1.5 × 10^−8^ substitutions per site per year for dicotyledonous plants^63^.

### Phylogenetic analysis

To analyze the evolutionary relationship, GST protein sequences from five different species- *Capsicum annuum*, *Arabidopsis thaliana*, *Oryza sativa*, *Solanum lycopersicum* and *Solanum tuberosum* were obtained from respective genome database and class information was gathered from the previously published literatures^14,15,28,36^. The phylogenetic tree was constructed using the default parameters of the maximum likelihood method in MEGA 7 software with 1000 bootstrap replicates. The James Taylor Thornton (JTT) substitution model was set with the site coverage cutoff of 95%. To investigate the lineage-specific expansion of *GST* genes in *Capsicum annuum* and *Solanum lycopersicum*, ten class-specific phylogenetic trees were constructed using MEGA 7 software according to the above mentioned procedure.

### Pepper RNA-seq data analysis

Illumina RNA sequenced gene expression profiling data of each *CaGST* genes at different tissues and developmental stages and in response to five abiotic stresses was obtained from the pepper hub transcriptome database (http://pepperhub.hzau.edu.cn/petdb/). For abiotic stress treatments, datasets were obtained from the database for 0, 1, 1.5, 3, 6, 12 and 24 h of cold, heat, drought, salinity and osmotic stress treatments for leaf and root tissue, and the relative fold change of expression was calculated based on their control value (0 h). Heat maps with hierarchical clustering were performed using the default parameters of TIGR Multiple Experiment Viewer (MEV) 4.9 software package with the Manhattan correlation^64^.

### Plant materials, stress treatment, and total GST activity

*C. annuum* (BARI Morich-3) were germinated and grown as described previously by Guo *et al*.^40^. Fifteen days old plants were soaked with normal water (for experimental control), or 200 mM NaCl (for salinity), or 5% mannitol (for drought stress), or 5 mM H_2_O_2_ (for oxidative stress), or normal water at 40^0^C (for heat stress) or 4^0^C (for cold stress). Shoot samples were collected at 0, 6, 12, and 24 hours post-stress induction. Total protein was extracted using the ice-cold extraction buffer containing 100 mM potassium phosphate buffer, pH 7.0, 50% glycerol, 16 mM MgSO_4_ and 1 mM PMSF^57^. After quantification of the protein by Bradford method^65^, GST activity was measured by its ability to conjugate the reduced glutathione and 1-chloro-2,4-dinitrobenzene at 344 nm^36^. Activity was calculated using the extinction coefficient of the product formed (9.6 mM^−1^ cm^−1^) and was expressed as nmoles of CDNB conjugated/minute/mg of total protein.

### Analysis of putative promoter sequence

The 1000 bp 5′ upstream sequences from the transcription start site of all the *CaGST* genes were acquired from the pepper hub (http://pepperhub.hzau.edu.cn/pegnm/) and then analyzed individually on the PlantCARE program (http://bioinformatics.psb.ugent.be/webtools/plantcare/html/) with default parameters for the identification of the important stress and hormone responsive cis-regulatory elements^66^.

## Supplementary information

Supplementary Information

## Data Availability

The authors declare that all the data and plant materials will be available without restrictions.
