# Supplementary material for: Genome-wide identification of glutathione S-transferase gene family in pepper, its classification, and expression profiling under different anatomical and environmental conditions
Source: Sci Rep. 2019 Jun 24;9:9101. doi: 10.1038/s41598-019-45320-x (PMC6591324; doi:10.1038/s41598-019-45320-x)
Supplement: Supplementary file 1 — Supplementary Information [file 41598_2019_45320_MOESM1_ESM.pdf]

Genome-wide identification of glutathione S-transferase gene family in pepper, its classification, and expression profiling under different anatomical and environmental factors

Short title: Analysis of pepper *GST* gene family

Shiful Islam[1,#], Saikat Das Sajib[1,#], Zakya Sultana Jui[2], Shatil Arabia[2], Tahmina Islam[2,*], Ajit Ghosh[1,3,*]

[1]Department of Biochemistry and Molecular Biology, Shahjalal University of Science and Technology, Sylhet-3114, Bangladesh, [2]Plant Breeding and Biotechnology Laboratory, Department of Botany, University of Dhaka, Dhaka-1000, Bangladesh, and [3]Max-Planck Institute for Plant Breeding Research, Carl-von-Linne-Weg 10, D-50829 Cologne, Germany.

# Authors contributed equally

*To whom correspondence should be addressed:

ajitghoshbd@gmail.com, subarna.islam@gmail.com

Supplementary Table S1. Prediction of secondary structure of all the identified CaGST proteins

No	Protein	Alpha helix (Hh) %	Extended Strand (Ee) %	Beta Turn (Tt) %	Random Coil (Cc) %
1	CaGSTU1	34.50%	23.58%	9.17%	32.75%
2	CaGSTU2	41.33%	18.22%	9.78%	30.67%
3	CaGSTU3	41.38%	17.73%	9.85%	31.03%
4	CaGSTU4	32.42%	24.20%	9.13%	34.25%
5	CaGSTU5	45.70%	21.27%	8.14%	24.89%
6	CaGSTU6	30.63%	26.13%	12.16%	31.08%
7	CaGSTU7	47.27%	19.09%	11.36%	22.27%
8	CaGSTU8	33.03%	21.10%	13.76%	32.11%
9	CaGSTU9	57.14%	11.61%	7.14%	24.11%
10	CaGSTU10	53.39%	16.74%	7.24%	22.62%
11	CaGSTU11	44.44%	20.89%	7.11%	27.56%
12	CaGSTU12	42.79%	17.12%	9.91%	30.18%
13	CaGSTU13	47.71%	15.69%	9.15%	27.45%
14	CaGSTU14	31.43%	26.67%	8.57%	33.33%
15	CaGSTU15	45.78%	20.44%	10.22%	23.56%
16	CaGSTU16	47.32%	16.52%	7.59%	28.57%
17	CaGSTU17	49.06%	14.62%	8.02%	28.30%
18	CaGSTU18	46.35%	20.60%	11.59%	21.46%
19	CaGSTU19	36.94%	27.03%	12.61%	23.42%
20	CaGSTU20	44.44%	15.56%	8.44%	31.56%
21	CaGSTU21	48.00%	15.56%	10.22%	26.22%
22	CaGSTU22	45.33%	17.78%	7.56%	29.33%
23	CaGSTU23	48.64%	15.00%	10.45%	25.91%
24	CaGSTU24	49.09%	16.36%	9.09%	25.45%
25	CaGSTU25	54.50%	12.61%	6.76%	26.13%
26	CaGSTU26	47.43%	16.00%	12.00%	24.57%
27	CaGSTU27	36.52%	24.82%	8.51%	30.14%
28	CaGSTU28	43.06%	22.49%	11.00%	23.44%
29	CaGSTU29	31.68%	19.80%	13.86%	34.65%
30	CaGSTU30	42.02%	19.33%	10.08%	28.57%
31	CaGSTU31	45.00%	16.36%	7.27%	31.36%
32	CaGSTU32	42.47%	18.72%	12.33%	26.48%
33	CaGSTU33	36.45%	23.65%	10.34%	29.56%
34	CaGSTU34	49.56%	17.26%	6.64%	26.55%
35	CaGSTU35	49.50%	12.87%	7.92%	29.70%
36	CaGSTU36	39.23%	17.68%	9.94%	33.15%
37	CaGSTU37	46.19%	17.49%	11.21%	25.11%
38	CaGSTU38	45.45%	17.73%	10.00%	26.82%
39	CaGSTU39	56.25%	10.27%	6.25%	27.23%
40	CaGSTU40	48.39%	17.05%	8.29%	26.27%
41	CaGSTU41	46.36%	17.27%	10.00%	26.36%
42	CaGSTU42	44.09%	18.18%	12.27%	25.45%
43	CaGSTU43	59.74%	7.79%	3.25%	29.22%
44	CaGSTU44	51.36%	14.55%	9.09%	25.00%
45	CaGSTU45	53.46%	17.51%	11.06%	17.97%
46	CaGSTU46	42.01%	17.35%	10.50%	30.14%

47	CaGSTU47	48.42%	20.36%	9.50%	21.72%
48	CaGSTU48	42.86%	18.30%	8.04%	30.80%
49	CaGSTU49	65.03%	7.69%	4.20%	23.08%
50	CaGSTU50	26.61%	25.69%	16.51%	31.19%
51	CaGSTU51	48.48%	17.75%	7.36%	26.41%
52	CaGSTU52	43.26%	16.28%	10.23%	30.23%
53	CaGSTU53	51.82%	14.09%	6.82%	27.27%
54	CaGSTU54	57.21%	11.79%	8.30%	22.71%
55	CaGSTU55	47.29%	17.73%	5.42%	29.56%
56	CaGSTU56	45.85%	16.16%	8.30%	29.69%
57	CaGSTU57	48.47%	14.41%	5.68%	31.44%
58	CaGSTU58	46.29%	14.41%	6.55%	32.75%
59	CaGSTU59	42.92%	25.11%	7.76%	24.20%
60	CaGSTF1	41.67%	20.61%	9.21%	28.51%
61	CaGSTF2	43.19%	19.25%	12.68%	24.88%
62	CaGSTF3	39.44%	16.43%	11.27%	32.86%
63	CaGSTF4	49.40%	18.33%	10.36%	21.91%
64	CaGSTF5	44.04%	16.06%	9.48%	30.43%
65	CaGSTF6	31.75%	22.22%	10.32%	35.71%
66	CaGSTT1	56.45%	4.84%	4.84%	33.87%
67	CaGSTT2	56.00%	13.60%	8.00%	22.40%
68	CaGSTT3	52.70%	13.69%	7.47%	26.14%
69	CaGSTT4	47.23%	12.34%	9.36%	31.06%
70	CaGSTZ1	47.35%	15.55%	9.19%	27.92%
71	CaGSTZ2	42.99%	19.63%	8.41%	28.97%
72	CaGSTL1	24.84%	23.25%	8.92%	42.99%
73	CaGSTL2	37.24%	15.06%	10.04%	37.66%
74	CaGSTL3	43.24%	16.67%	6.76%	33.33%
75	CaGSTL4	40.32%	17.39%	6.32%	35.97%
76	CaEF1Bɣ1	36.71%	23.19%	7.49%	32.61%
77	CaEF1Bɣ2	34.31%	16.67%	5.88%	43.14%
78	CaDHAR1	34.92%	19.66%	7.12%	38.31%
79	CaDHAR2	36.32%	18.40%	8.96%	36.32%
80	CaTCHQD	56.72%	13.06%	10.82%	19.40%
81	CaMGST1	31.94%	30.56%	11.81%	25.69%
82	CaMGST2	52.94%	13.31%	6.81%	26.93%
83	CaGHR1	42.38%	17.73%	8.59%	31.30%
84	CaGHR2	35.77%	14.63%	8.13%	41.46%
85	CaGHR3	29.85%	17.91%	7.46%	44.78%

Supplementary Table S2. Prediction of glycation of all the identified CaGST proteins

Name	No of site	Position	Region	Score
CaGSTU2	1	176	NFSI	0.6209
CaGSTU3	1	23	NKST	0.5614
CaGSTU4	3	36	NLSN	0.7626
		39	NKSP	0.1363
		172	NFSM	0.5490
CaGSTU5	2	10	NGSP	0.1893
		38	NKSQ	0.5869
CaGSTU9	1	38	NKSE	0.6150
CaGSTU10	1	38	NKSA	0.6329
CaGSTU13	2	89	NASA	0.4108
		124	NYSE	0.5454
CaGSTU15	2	38	NKSS	0.6822
		191	NCSQ	0.5816
CaGSTU17	2	30	NKSP	0.1359
		56	NESS	0.6142
CaGSTU18	1	42	NKSS	0.6111
CaGSTU19	1	183	NLSN	0.6634
CaGSTU23	2	38	NKSP	0.1348
		190	NCTQ	0.5456
CaGSTU24	2	40	NKSP	0.1363
		173	NFSI	0.6032
CaGSTU27	1	236	NFSI	0.5124
CaGSTU28	2	40	NKSP	0.1522
		168	NFSI	0.5378
CaGSTU29	1	43	NKSS	0.5653
CaGSTU30	1	40	NKSL	0.5996
CaGSTU33	1	23	NKST	0.5616
CaGSTU36	1	125	NYTA	0.7327
CaGSTU39	2	39	NKSP	0.1733
		75	NETW	0.5160
CaGSTU44	2	38	NKSP	0.1349
		190	NCTQ	0.5458
CaGSTU48	2	38	NKSS	0. 6051
		190	NCSQ	0.5041
CaGSTU49	1	110	NCSQ	0.5886
CaGSTU51	4	78	NWTN	0.6588
		144	NSSK	0.3416
		179	NVTL	0.6576
		184	NESK	0.5117
CaGSTU55	2	40	NKSE	0.5899
		141	NKSE	0.5211
CaGSTU56	1	40	NKSE	0.5930
CaGSTU57	1	40	NKSE	0.5713
CaGSTU58	2	40	NKSE	0.5714
		177	NETK	0.5556
CaGSTF4	3	47	NPSG	0.6092
		57	NGSH	0.6827
		84	NSTT	0.4386
CaGSTF5	7	28	NQTE	0.6330
		34	NLSM	0.6389

		57	NDSN	0.6320
		60	NIST	0.4849
		95	NRTK	0.7214
		382	NISA	0.4080
		614	NGTD	0.5418
CaGSTF6	2	128	NGTD	0.6176
		193	NMTE	0.6732
CaGSTT3	1	25	NLSK	0.7566
CaGSTZ1	1	51	NASS	0.6736
CaGSTZ2	1	91	NYSS	0.6114
CaGSTL1	1	220	NATF	0.5199
CaDHAR1	3	25	NTTI	0.7488
		32	NSTK	0.7172
		252	NWSI	0.3783
CaDHAR2	2	43	NVSD	0.7169
		86	NPSL	0.7227
CaTCHQD	3	47	NPSA	0.6847
		57	NGSH	0.6511
		87	NPSS	0.7076
CaMGST2	4	38	NKSY	0.5411
		155	NSSD	0.6768
		217	NFSF	0.5964
		246	NITD	0.7551
CaGHR1	5	12	NRSI	0.6308
		158	NYSG	0.5413
		179	NESS	0.5497
		213	NETN	0.4870
		305	NYTK	0.6772
CaGHR2	2	7	NGSS	0.7101
		41	NETN	0.5730

Supplementary Table S3. List of putative duplicated *CaGST* members

No	Member 1	Member 2	Identity (%)	d_N	d_S	d_N/d_S	Duplication time (Mya)	Selection type	Duplicate type
1	CaGSTU3	CaGSTU33	97	0.0248	0.0355	0.7000	1.18	Purifying	Segmental
2	CaGSTU8	CaGSTU50	92	0.0755	0.1069	0.7060	3.56	Purifying	Segmental
		CaGSTU11	89	0.1049	0.1668	0.6288	5.56	Purifying	Segmental
3	CaGSTU13	CaGSTU42	83	0.1403	0.4191	0.3349	13.97	Purifying	Segmental
		CaGSTU46	94	0.0267	0.0587	0.4558	1.95	Purifying	Segmental
4	CaGSTU14	CaGSTU47	93	0.0413	0.0975	0.4235	3.25	Purifying	Segmental
5	CaGSTU20	CaGSTU21	96	0.0318	0.0658	0.4830	2.19	Purifying	Segmental
		CaGSTU22	96	0.0336	0.0616	0.5454	2.05	Purifying	Segmental
6	CaGSTU23	CaGSTU44	98	0.0076	0.0426	0.1789	1.42	Purifying	Segmental
		CaGSTU45	84	0.1271	0.5363	0.2371	17.84	Purifying	Segmental
7	CaGSTU24	CaGSTU25	89	0.0809	0.3491	0.2317	11.64	Purifying	Tandem
		CaGSTU26	86	0.1176	0.4878	0.2411	16.26	Purifying	Tandem
		CaGSTU27	82	0.1231	0.5136	0.2397	17.12	Purifying	Tandem
		CaGSTU28	82	0.0994	0.5114	0.1943	17.04	Purifying	Tandem
		CaGSTU29	85	0.0829	0.2926	0.2834	9.75	Purifying	Tandem
		CaGSTU30	90	0.0834	0.2192	0.3805	7.31	Purifying	Tandem
		CaGSTU31	88	0.0823	0.3667	0.2243	12.22	Purifying	Tandem
8	CaGSTU34	CaGSTU35	96	0.0464	0.0580	0.7988	1.93	Purifying	Tandem
9	CaGSTU56	CaGSTU54	85	0.1526	0.1863	0.8191	6.21	Purifying	Tandem
		CaGSTU57	89	0.0830	0.1887	0.4402	6.29	Purifying	Tandem
		CaGSTU58	89	0.0807	0.1890	0.4269	6.3	Purifying	Tandem
10	CaGSTL3	CaGSTL4	93	0.0615	0.0799	0.7694	2.66	Purifying	Segmental
11	CaEF1Bɣ1	CaEF1Bɣ2	93	0.0324	0.0716	0.4522	2.38	Purifying	Segmental
12	CaGHR1	CaGHR2	85	0.1361	0.2811	0.4841	9.37	Purifying	Tandem

Supplementary Table S4. Detailed information of putative conserved motifs in the CaGST proteins

No.	Motif sequence	Sites	Length (aa)	E-value	Corresponding GST domain
1	LESNPIHKKIPVLIHNGKPICESMIIVEYIDETFEG	58	36	4.0e-1421	N-Terminal
2	PSILPSDPYDRAIARFWASYI	55	21	3.8e-745	C-terminal
3	HRVEWALKIKGVEYEFIEENLQNKSP	65	26	1.1e-692	N-terminal
4	LGDKPYFGGDNFGFVDIAL	57	19	2.5e-442	C-terminal
5	FSTEAECPKFVAWAKRCMQRDSVAKSLPDQHKVLEF	51	36	3.4e-324	C-terminal
6	SIFFKGEEQEKAKEEVYEMLKVLDNE	44	26	6.0e-347	C-terminal
7	MAQVKLLGFWYSPF	53	14	8.4e-249	N-Terminal
8	PFITLNPFGQVPAFEDGDLKLFESRAITQYIAHTYA	16	36	3.2e-123	N-Terminal
9	YVFSTVLAPSFGLTLNPQAAAEAEKVLLTSLAKIESVWLQKKGQFLLGS GQPSIADLSLVCELMQLEVLDEKDRERIIGPYKRVLKWIDDTKNAMQP	4	97	2.4e-098	C-terminal
10	KTTNHENDNLGLLQILLVTLFGSYKVYEEVLGMKILVPENTPLLYSCVTS LNKLPLVKEVCPPHDKMVA	4	69	6.8e-087	C-terminal

Supplementary Table S5. Presence of cis regulatory elements of the promoter of all the identified *CaGST* genes

Motif / Gene	ABRE	AuxR R-core	Box-W1	ERE	GARE	HSE	LTR	MBS	TC rich repeat	TCA element	TGACG motif	5’-UTR py-rich stretch	W-box	p-box	WUN	Total
	TACGTG	GGTCCAT	TTGACC	ATTTCAAA	AAACAGA	AAAAAATTTC	CCGAAA	TAACTG	ATTCTCTAAC	GAGAAGAATA	TGACG	TTTCTTCTCT	TTGACC	CCTTTTG	TCATTACGAA	
CaGSTU1					1	1		1	1	2		3				9
CaGSTU2										1	1	1				4
CaGSTU3			1	1	1	2		1	1	1		1	1			10
CaGSTU4	1		2			1		1	2	2	1		2			12
CaGSTU5						1	1					1				3
CaGSTU6									1	1	1					3
CaGSTU7	1									1						2
CaGSTU8				2												2
CaGSTU9	1							1			2			1		5
CaGSTU10		1	1				1	3					1	2		9
CaGSTU11					2			2		2	2			1		9
CaGSTU12	1							1		1					2	5
CaGSTU13			1	1				3	3				1			9
CaGSTU14					9										1	10
CaGSTU15			1			2	1	1	1				1			7
CaGSTU16			1		1	1		1	1	1	1		1			8
CaGSTU17				1	1					2				1		5
CaGSTU18	3		1		1	1	2		1	1			1	1		12
CaGSTU19		1					1	1	1		1				1	6
CaGSTU20					1						2					3
CaGSTU21		1	1			1		1					1			5
CaGSTU22	1		1			1		1	1	1	2		1			9
CaGSTU23				2						1						3
CaGSTU24	2			1		1		1	2	2	1					10
CaGSTU25						1	1		1	1						4
CaGSTU26	1			1	1				6	3	1					13
CaGSTU27		1			1			3	2	1	1					9
CaGSTU28	2	1				3		1	2							10
CaGSTU29								2	1		2					5
CaGSTU30	2					1				1	1	3		1		9
CaGSTU31	2		1						1	1	1		1			7
CaGSTU32			1		1			2	1	2			1	1		9
CaGSTU33			1		1				1	1			1			5
CaGSTU34	1				1				4	1						7
CaGSTU35	1				1	1		1		2		1				7
CaGSTU36	1				1			3	2	1				3		10
CaGSTU37			1			1		1					1			4
CaGSTU38			1		1	1		2			1		1			7
CaGSTU39	2		1			1			1		2		1			8
CaGSTU40	1				1				1							3
CaGSTU41						3			1			1				5
CaGSTU42	1				1	3			1	2	1	1				10
CaGSTU43	1			1	1			1	3		1	1				8
CaGSTU44					1	1		2	3			4				11
CaGSTU45			2			2		2	2		1	1	2			12
CaGSTU46	1		1	1		1		3	4				1			12
CaGSTU47					1			1	1	1	1					5
CaGSTU48					1			1		1	1				1	5
CaGSTU49			1					2		2	1		1			7
CaGSTU50				3		1				1						5
CaGSTU51						1										1
CaGSTU52					2	1			1							4
CaGSTU53			1			1		1			1		1			5
CaGSTU54			1			3			2		1		1			8
CaGSTU55	1										1	1		1		4
CaGSTU56						1	3		1							5
CaGSTU57			1			1		1	1	1	1		1			7
CaGSTU58						1	2	1		1						5
CaGSTU59			1					2	1	2			1	1	1	9
CaGSTF1	1		2	1		1		2					2			9
CaGSTF2			2		1			1					2			6
CaGSTF3				2		4			1							7
CaGSTF4								2	2	2	1	1				8
CaGSTF5				1			1	2		1	1					6
CaGSTF6								2	2	3		1		1		9
CaGSTT1					2				1		1	1		1		6
CaGSTT2						2		1	1	1		1				6
CaGSTT3				1		1	1			1						4
CaGSTT4			1			1			1	2	2	1	1	1		10
CaGSTZ1	2		2	2		1						1	2	1		11
CaGSTZ2					1		1		3	2	1					8
CaGSTL1						1		6				1				8
CaGSTL2	2	1	1			2			3		1		1			11
CaGSTL3			1					3	1	2	4		1			12
CaGSTL4	1				1			2	1	2						7
CaEF1Bg1	1		1					5					1	1		9
CaEF1Bg2			1		2	1		1					1	2		8
CaDHAR1			1				1	2	1				1			6
CaDHAR2			1					1	1		1		1	1		6
CaTCHQD			1	1		4	1	2	3		1		1	1		15
CaMGST1						1										1
CaMGST2			2					2	1	1	2		2			10
CaGHR1						2			1		2					5
CaGHR2						1	1			1				1		4
CaGHR3						1			1	1					1	4
**Total**	34	6	40	22	40	**64**	18	**85**	**83**	**63**	50	26	40	23	7	601

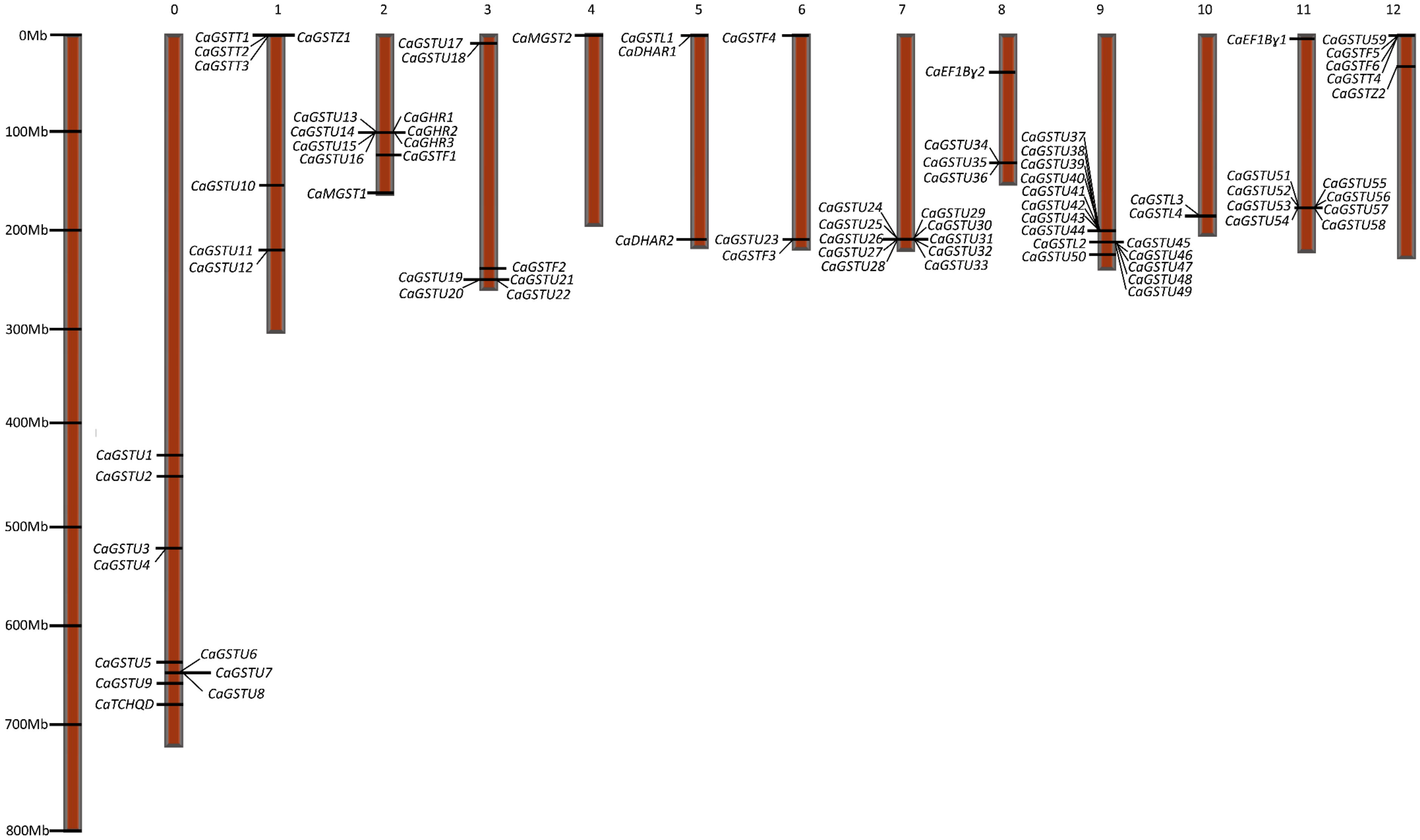

Figure S1. Chromosomal localization of *CaGST* genes. All the identified genes were positioned in their corresponding chromosome of pepper. The size of the chromosome and relative position of the gene could be inferred using the provided vertical scale (Mb). The respective chromosome numbers are designated on the top of each chromosome.

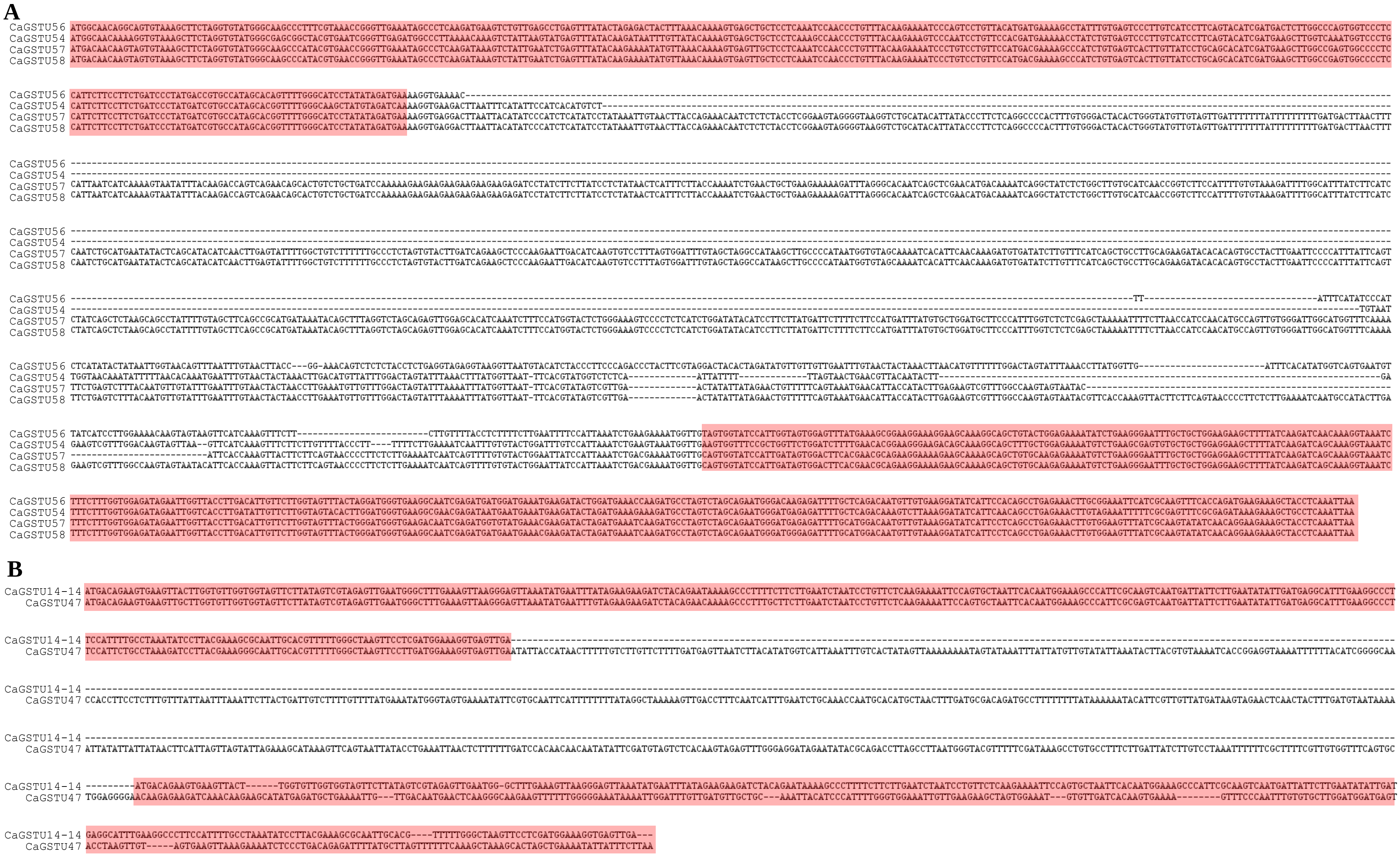

Figure S2. Alignment of CaGST genomic DNA. Genomic DNA of CaGSTU54, 56-58 (A) and CaGSTU14-CaGSTU14, CaGSTU47 (B) were aligned to identify the exon and intron position. Exon sequences were marked with red shade. This indicates the extension of intron in figure A, and exon duplication in figure B.

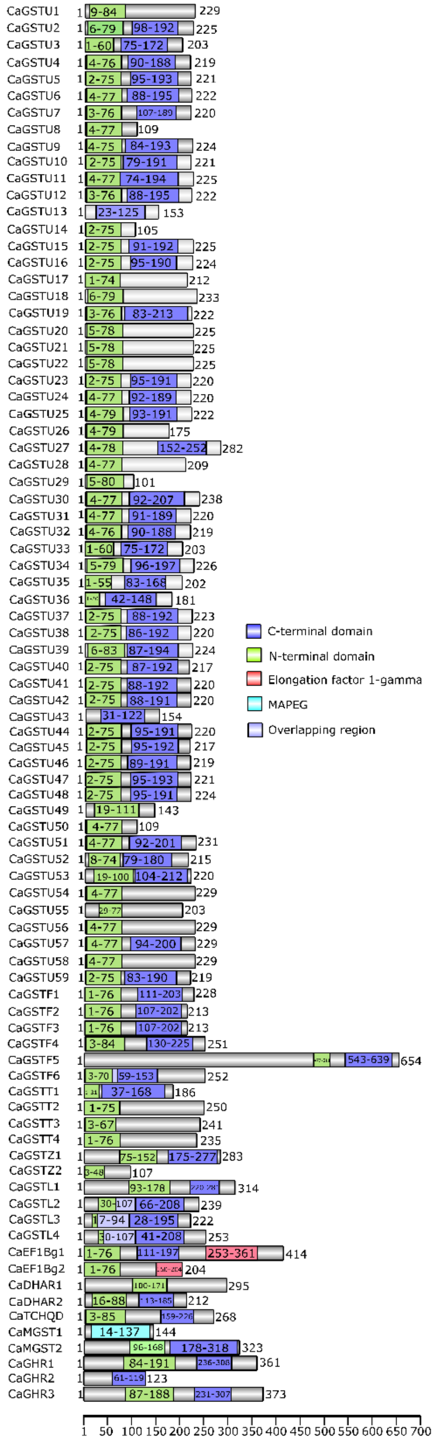

Figure S3. Conserved domain arrangement of CaGST proteins. All the identified CaGST proteins were analyzed to identify the presence of functionally conserved domain(s) using Pfam. The domain of GST_N terminal (PF02798.18), GST_C terminal (PF00043.23), EF1Bγ (PF00736), microsomal (PF01124.16), and overlapping regions are highlighted by green, blue, red, cyan and light blue boxes, respectively. The position of the domain and total protein length is indicated by the exact amino acid number inside the box and ending, respectively. The relative position/size could be interpreted by the provided scale in amino acid number.

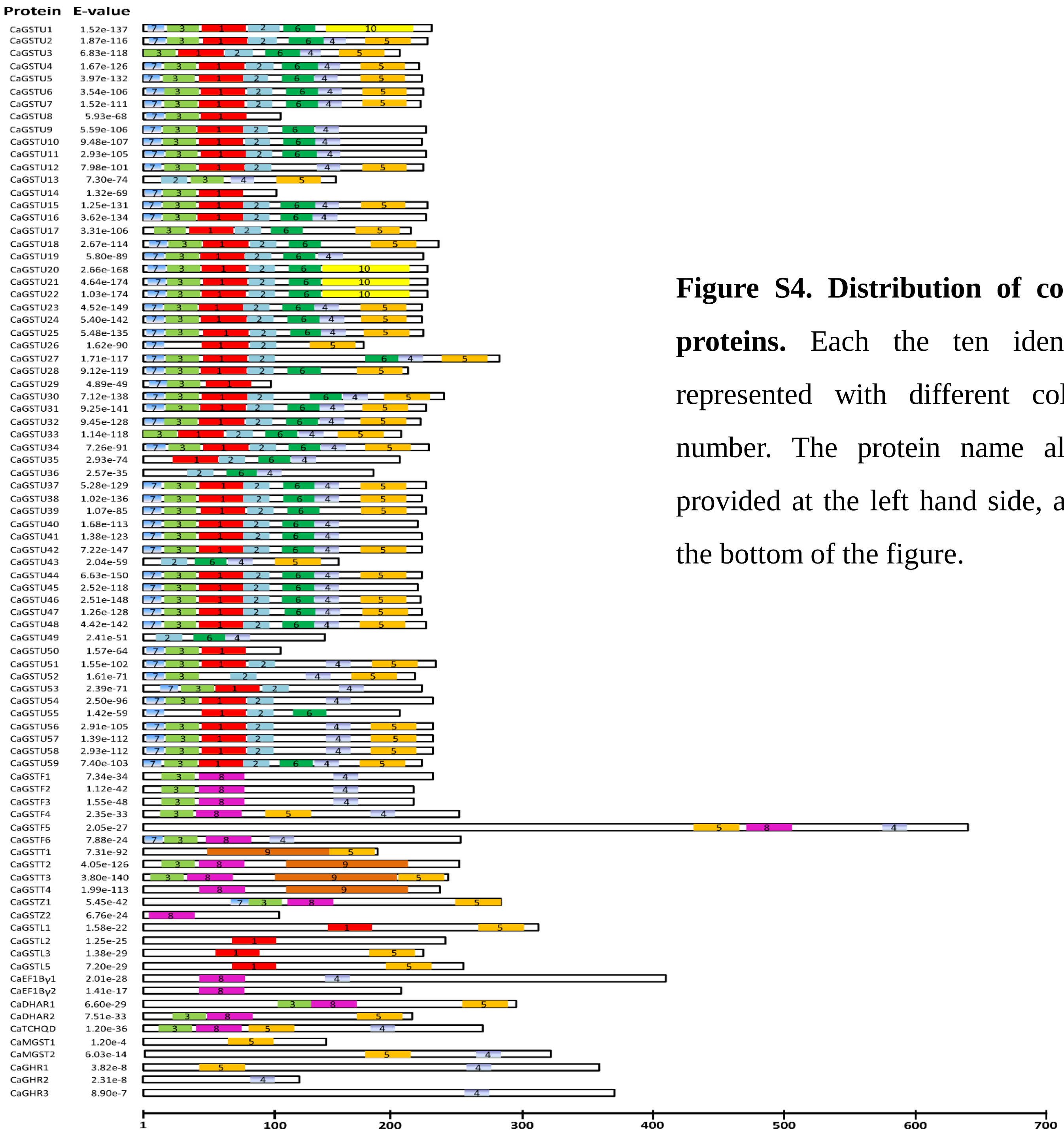

Figure S4. Distribution of conserved motifs in CaGST proteins. Each the ten identified putative motifs are represented with different colored box with embedded number. The protein name along with the E-value are provided at the left hand side, and the motif size scale is at the bottom of the figure.

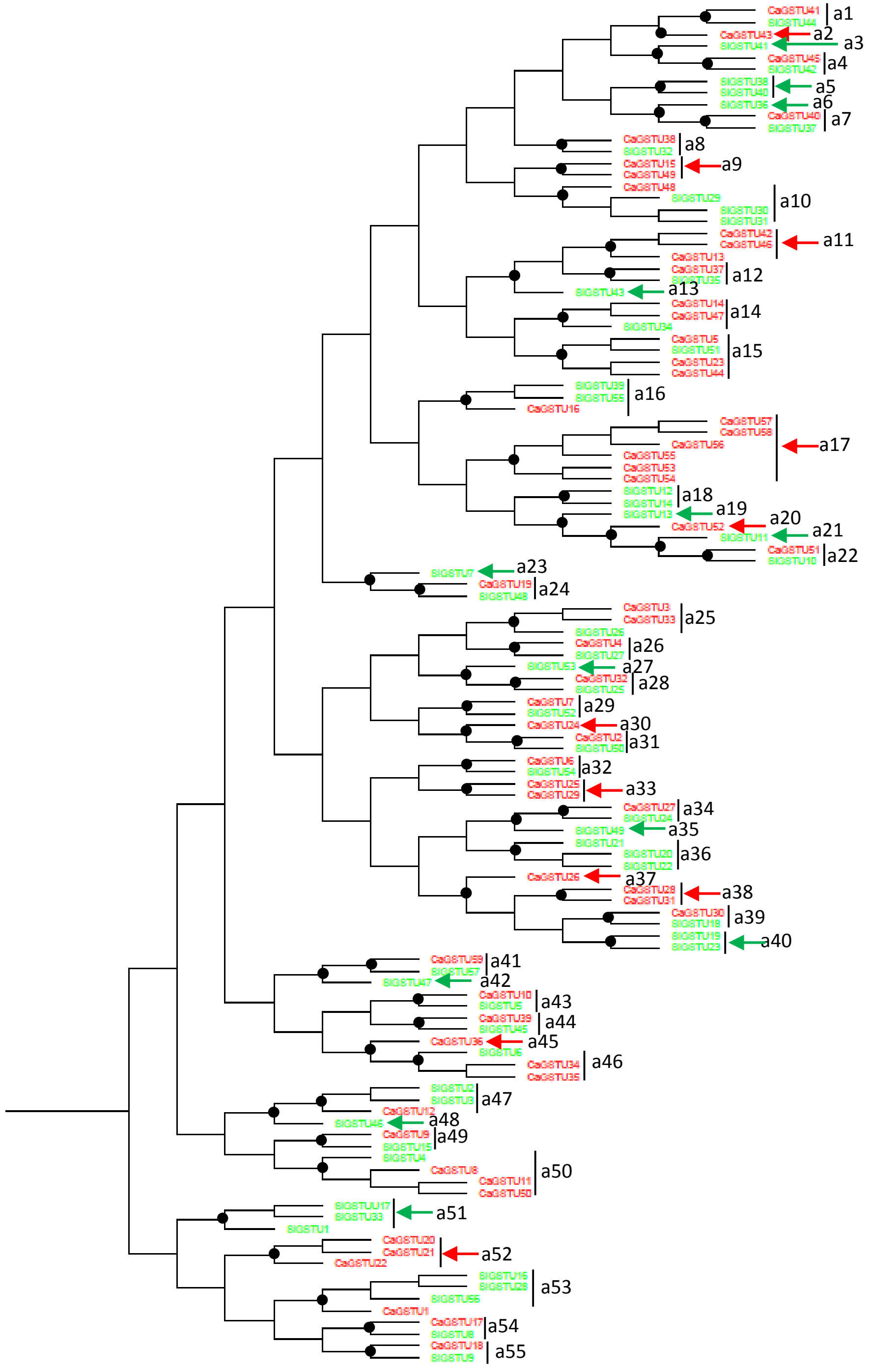

2.0

Figure S5. Phylogenetic relationships of the *Capsicum* and tomato tau GSTs.

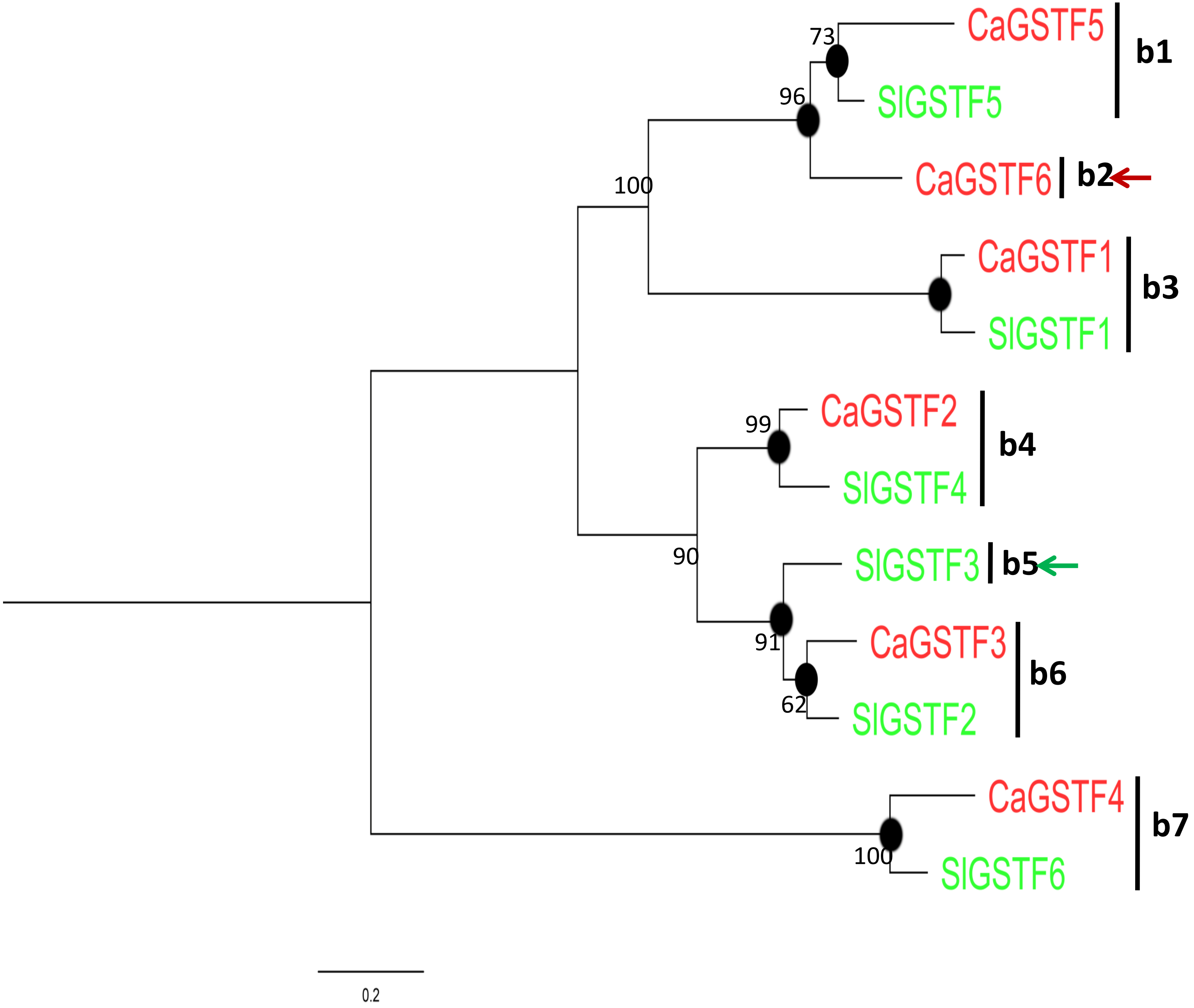

Figure S6. Phylogenetic relationships of the *Capsicum* and tomato phi GSTs.

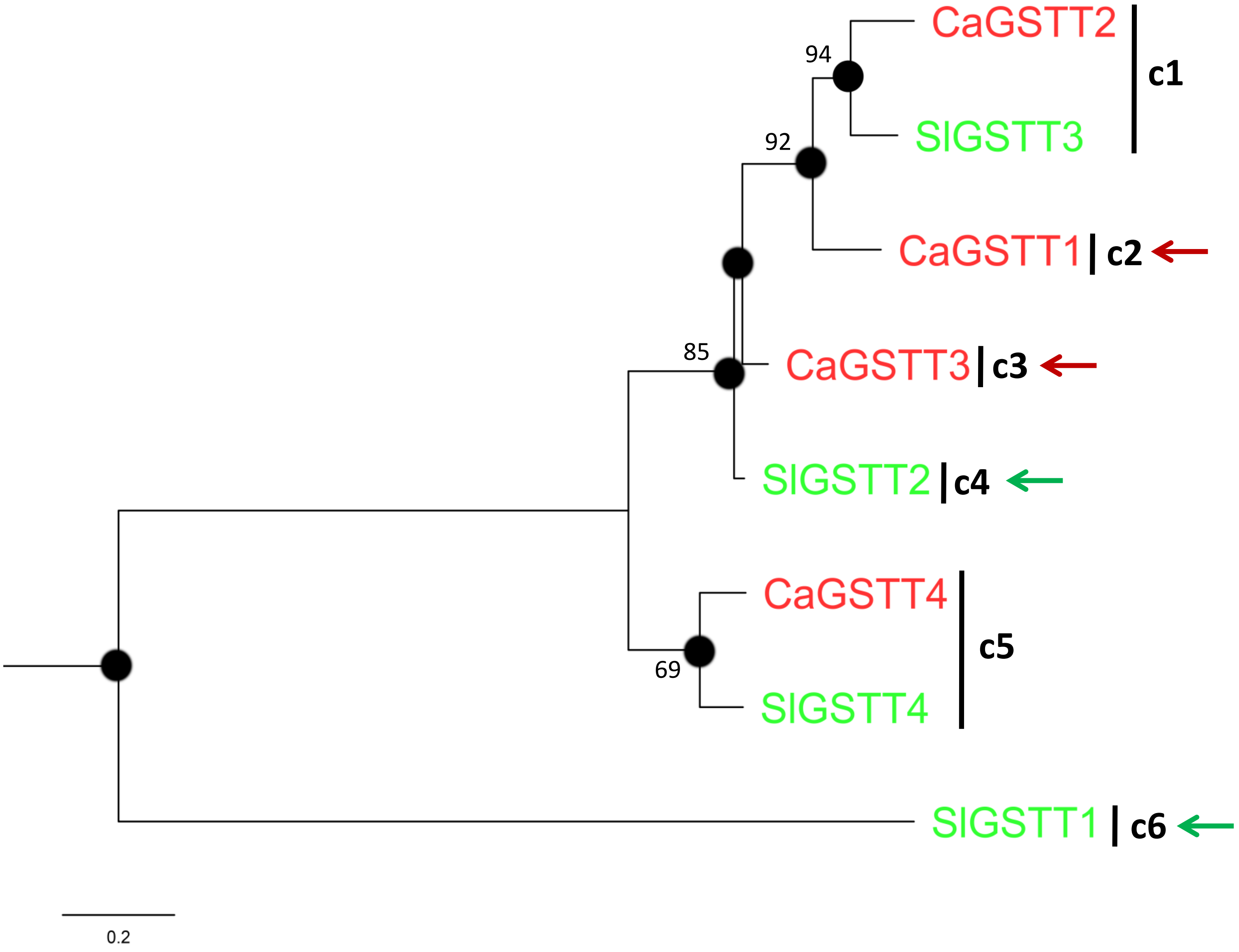

Figure S7. Phylogenetic relationships of the *Capsicum* and tomato theta GSTs.

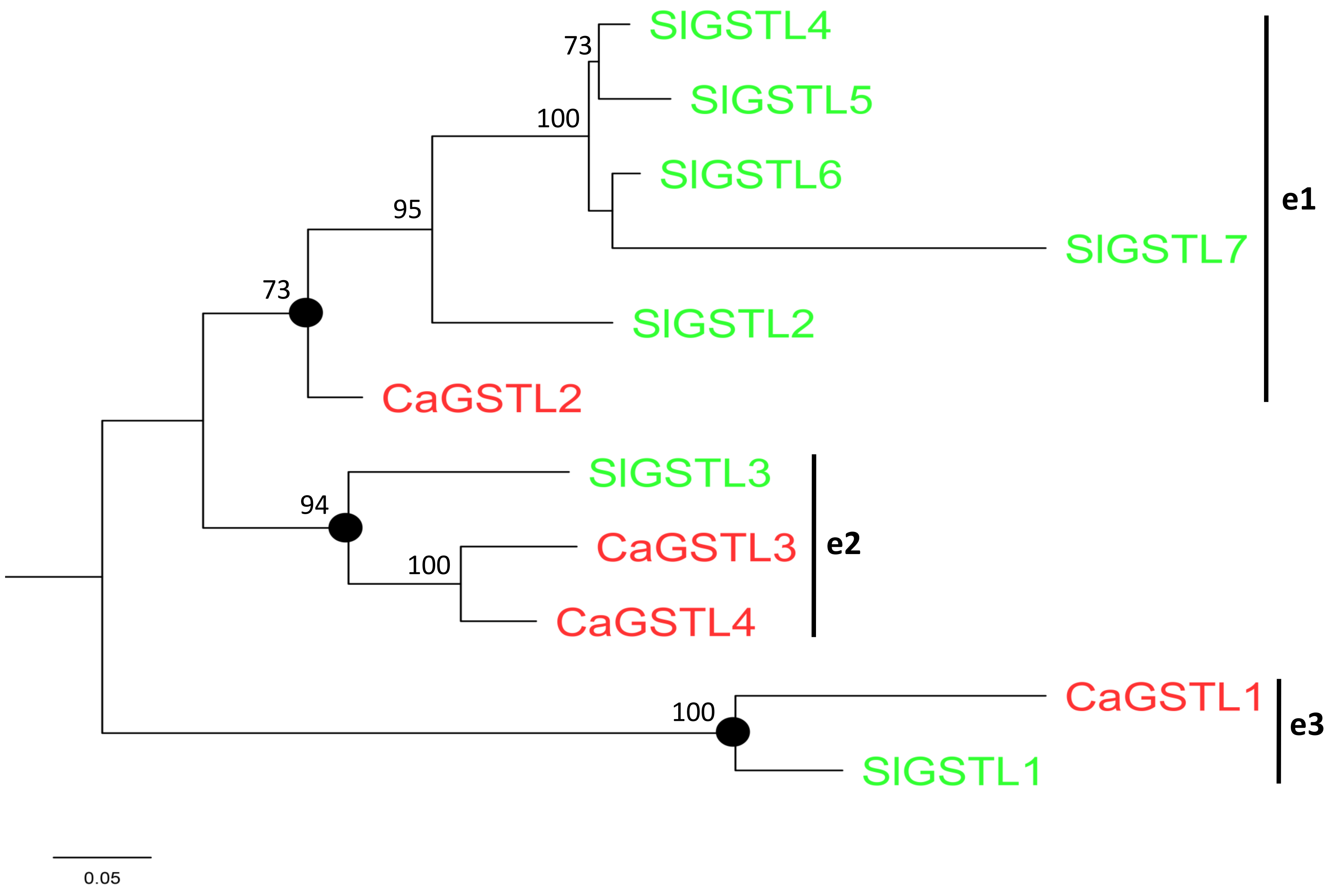

Figure S8. Phylogenetic relationships of the *Capsicum* and tomato lambda GSTs.

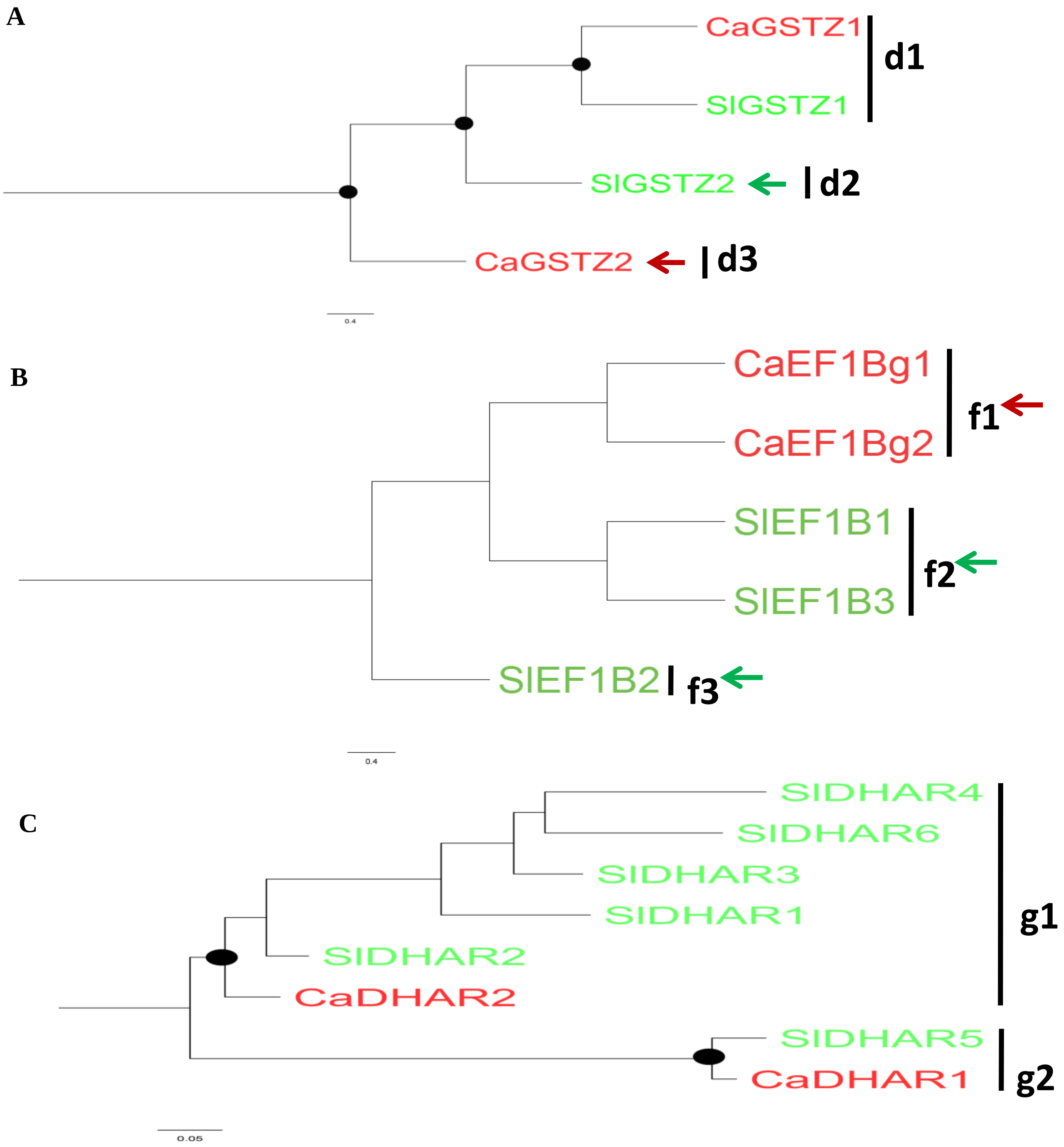

Figure S9. Phylogenetic relationships of the *Capsicum* and tomato zeta GSTs (A), EF1Bγ GSTs (B) and DHAR (C).

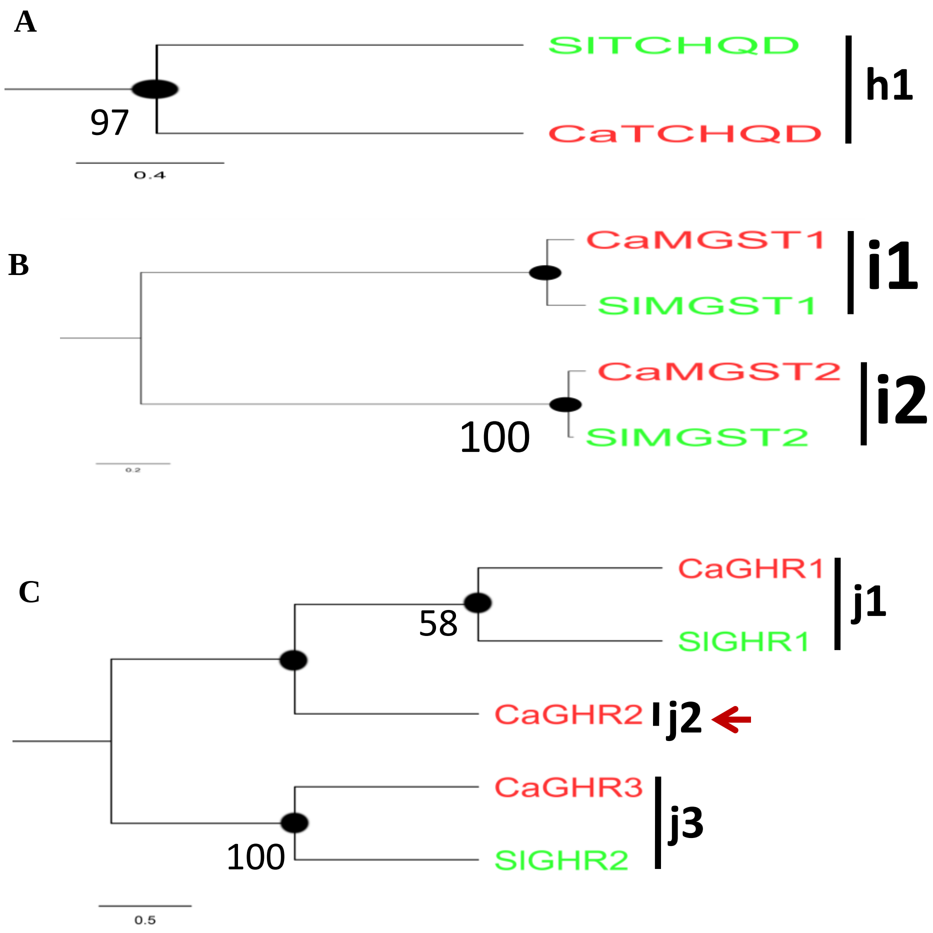

Figure S10. Phylogenetic relationships of the *Capsicum* and tomato TCHQD GSTs (A), MGSTs (B) and GHRs (C).

Text S1. GST protein sequences used in the used to generate phylogenetic tree

>CaGSTU1
MGEENKVTLHGSWASPYVKRVELALKVKGIPFEYVEEDLRDKSPLLLKYNPIHKKVPVLVHNGKPVSESFVILEYIDETWKNQPRLFPQDPYERARV
RFWASFIQQVIDCMLKVFKGVEQDKALEEFYEKLSVLEDGIKNSSLGITTTNIQGRDLGLLDIIIVITLGAYEVQEEVFGVKILSPEKTPLLYSWVT
TLIDLPIVKGITPPRDKVVPFLHFLKDVVFKAPMN
>CaGSTU2
MEGTNNDVVLLDFWPSSFGMRLRIALALKGIQYEGREENLFDKSTLLLEMNSVHKKIPVLIHNGKPICESLIILEYIDQVWHDKYPLLPSHPYDKSQ
ARFWADYVDKKIYSTGRRVWSGKGEDQEEAKKEFIEILKTLEGELGDKTYFGGDDNLGFVDVALVPFTSWFYSYEICANFSIEAVCPKLVAWARRCM
QIESVANSLPHPHKIYDYVLELKHKFGLDTN
>CaGSTU3
MRVRIALAEKGIQYEYKEQDLLNKSTLLLQMNPIHKKIPVLIHNGKPICESHIIVEYIDEVWKDKSPLLPSDPYKRAHARFWTDYIGKKIYDAGMKI
WSSKVEVHETANKEFINCLKVLEGELGDKPYFNGENFGLVDMAFIPFYSWFPVYKKLSNLNIVEECPKFVSWAKRCMQKDSVSKCLVDPDAIYDFIV
MFRQKIGVA
>CaGSTU4
MGDEVVLLDFWPSPFGMRVRIALKEKGIDYEYKEENLSNKSPLLLKMNPIHKQIPVLIHNGKPVCESLIIVHYIDEVWKDKTPLLPSHPYERAHARF
WADYVDKKIYSTGRLVWGAKGEVQEAAKKELIQCFKLLEREVGDKNFFGGDQFGLVDIALIPFYSWFYALETCGNFSMIHECPKLVEWAKRCMKRES
VSTSLPDQYKVYDFILELKKKLELD
>CaGSTU5
MSGVKLLGINGSPASRRVEWALKIKGVEYEFIEEDLQNKSQLLLESNPVYKKIPVLLHNGKPIAESLVIIEYIDEVFEGPCILPKDPYDRAIARFWA
KFLDEKCLPAVWKALWSQGEEQEEDKKEAYEVLKVLDNELKDKKFFGGDGIGFVDIAANFVGFWIGIVEEATGVVLVTSEKFPNFCAWRNKYLNCDQ
VKENMPSREMLLGYFKSRVQAVANGPK
>CaGSTU6
MEEEEVILLDYWPSPFGSTARIALAEKGTNYIHKLEDLSSKSPLLLEMNPVHAKVPVLVHKGKPICESDIIIQYIDEVWKNNFSLLPSDPYQRAKAR
FLVDFINKKVHESSVKIWMGANEEQEKGKEELIEWSKILEEELGDKLYFGGDVFGFVDITLVPFYNWFIVFKTIANFYIIETECPKLVMWGGRCLNR
HSVSKSLPTKDQVYQAYLEFKKGWTHGQ
>CaGSTU7
MAEEVTLLDFWCSMYGMRARIALAEKGVKYEYKEEDLKNKSPLLLQMNPIHKKIPVLIHNGKSICESLVIVQYVDDVWKGTGPVLIPSDPYEKAQAW
FWSDYMDNTEHEYARKTWASKGEEQKQAIKDFLGGLKLLEGVLGDKPYFGGDSFGFLDVSLIGYYSWFLAYETFGKFNVESECPKLISWVKRCLERD
SVSKALPDPQKVCEFVLQLRKKFGLE
>CaGSTU8
MAGEKVKFLGYWASSFALRVHWVLKLKGIEYDYQEEHLPNKSPLLLQYNPVYKKIPVLIHNGKPIAESLVILEYIEDTWKHNPILPEDPYEQSKAHF
WVKFVNDKVITN
>CaGSTU9
MREVKVIGSSASLFYTRVEWALKLKGVTYEYIQEDLLNKSELLLRSNPVHKKIPVLLHDDKPVAESLVILEYIDETWKGYSLLPQDPYERATARFWA
KFVDEKCVIGSWQAMAAEGEAKEKAIESVQEQYAFIEKQIRGKKFFGGEQIGYLDLVMGWKTLWLNAMEELGIMKLLDPEKFPSLHQWTENFKQIPI
IQESMPPQESLVNYFQGGLNYVRSLAANKP
>CaGSTU10
MAEVKVHGIFAGPFNKRVELALKLKGVEYEYIEEDRSNKSAELVKYNPIYKQVPVLVHNGKPICESLIILEYIDETWESAAPLLPNDPYQRSIARFC
ANLIDDKLMGAMYKVCYGKGEEKEKGLDEVSEVLKYLDNELQDKKFFGGDNIGFLDIVASYIALWFGAIQEAIGMELLTKQKFTKLSKWIDEFLCCG
IVMEHLPTRESLVPLYIAQFEAAKASS
>CaGSTU11
MAGEKVKLLGYWASPFTLRVHWALKLKGIEYDYQEEDLPNKSPLILQCNPVHKKIPVLVHDGKPIAESLVILEYIEETWKHNPILPEDPYERAKARF
WVKVDDKCVPGIFGTFSKVGEEQRKIAKEARENLKILEGELGKKRFFGDTKIGFMDVAAAWIICWAQIVGEIVDIKLVDAEEMPSLVSWFQNVLEAA
PVLKECTPPKDKLLEHNKGFHKMLVASAST
>CaGSTU12
MADEVKLYRTWSSPFGLRIVWALHIKGIEYDTIFEDLSNKSPELLQYNPVHKKIPILVHKGKPICESLVILEYIDETWKEVPLLPQDPYQKAMARFW
AKFGEDKLLPSVWSVFSGKGVEEKKEALVPAVQNLQHLEEQLKDNKFFGGEKIGYLDIEFGWLPYLLDVFEEVIDLKLFDAAKFPLLSAWMKNFNDH
PAIKPHYPPRDKLVTKFQLLHEKYQTGN
>CaGSTU13
MVIVEYIDETFEGPPILPKDPYDRAIAHFWAKFFAEKGPAVGRSFLLKGEEQEKAKEEVYEMLKVLDNELKDKKFFVGDKFGFADITANASALWLGV
LEEASGVVLVTSEMLPNFCAWRDQYINYSENKKYLPLRDKLLAKLKARVQPAAAPK
>CaGSTU14
MTEVKLLGVGGSSYSRRVEWALKVKGVKYEFIEEDLQNKSPFLLESNPVLKKIPVLIHNGKPIRKSMIILEYIDEAFEGPSILPKYPYESAIARFWA
KFLDGKVS
>CaGSTU15
MAQVKLLGFWYSPFTHRVEWALKMKGVEYEYIEEDRHNKSSLLLESNPIHKKTPVLIHNGKPICESMFIVEYIDETFKGPSILPKDPYDRALAHFWA
KFLEDKSTVAIDILLHLGEEQEKAKEEVYEMLKVLDNELKDKNFFVAENFGFADIAANLMGFWLGIFLEVSGVTDLVTSEKIPNFCAWRDEYVNCSQ
VKDYLPPRKDELLVYFQLARARIEAAASAKK
>CaGSTU16
MAEVKLLGVWYSPANHRVEWALKIKGVEYEFIEQDLQNKSPLLLESNPVHRKIPVLIHNGKPICESLIIVEYIDETFEGPSILPKDPYDRALARFWS
KYLDDKVPAVVNTFFLKEEQEKAKEEVCEMLKVLDNELKDKKFLVADNFGLADITANFVGHWLGVFQEATGVELVTSEKFPNFCAWRDEYINCSQVK
EYPLPRRDELLAFVQARARAAASASASTRK
>CaGSTU17
MWLSPYVKRVELALKVKGITFEYVEEDLSNKSPLILKYNPVHKQVPVLVHDGKPINESSVILEYIDETWTNGPSLLPEDPYERSKVRFWAAYIQHVM
EFMLKIFTAEDQEKACEEFFQKLRVLEDGMKNMFPGGSPMIEDGKIGLLDIMIVVAFGMCKAQEQVFGIKFLDQEKAPMIHSWVNSLLELAVLKETV
PPHDKVVSFLQAFKERSH
>CaGSTU18

MEDENNKVTLHGMWISTYAKKVELALKIKGITFDYVEEDLSNKSSLLLQYNPIYKKVPVLLHRGKPVSESLVILEYIDETWKNERRFLPEDPYERAT
VRFWATYCLQMSDTMKKAFISDKDVEEGAFDEFFEKLKVMEEGMKDFFPGGRSIICADNLRLLDIIIVCSLGTYKAAEEVLGMKILDPEKYPFVYSW
VNTLLELPLVKETLPQHDKVVSRLEFIKQNGFTFQSHIS
>CaGSTU19
QMEEVKLHGTSYNLFTYRVIWALKLKGIPFEYIEEEHSDKGSLIMKYNPAFKRFPILFHGDKVISESMVIIEYIEDIWPQNPLLPIDPFDRAISRFW
VKFAGDKGHCIGTMYYTSGEKQENAIKETIEMLKIIEEQALDEENNFFGGEKIGIVDIAFGMIPHWLEIIEDVVGVKLLQPNLFPNLSNWVKNFKEE
KVIKENLPNCDEMFVFLKNQREMILSSS
>CaGSTU20
MAEENKVILHTTWASSYAMRVELALKIKGIQFEYVDEDLSNKSPELLKYNPVHKKVPLLVHNGKPVSESLIIILEYIDETWKTGPLILPQDPYERARV
RFWANFIDQHLIPSTAKVWSTTGEEQQKAQEEILEKLRILEEGLKTTNHENDNLGLLQILLVTLFGSYKAYEEVLGVKILVPENTPLLYSCVTSLNK
LPLAKEICPPHDKLVALLNVYRQKLLKSNTN
>CaGSTU21
MAEENKVILHKTYASSYAMRVELALKIKGIQFEYVDEDLSNKSPELLKYNPVYKKVPLLVHNGKPVSESLIIILEYIDETWKTGPLILPQDPYERARV
RFWANFIDQHLIPSTRKVWSTTGEEQEKAKEEIMEKLRILEEELKTTNHENDNLGLLQILLVTLFGSYKVYEEVLGMKILVPENTPLLYSCVTSLNK
LPLVKEVCPPHDKMVALLNVYRQKLLKSNTN
>CaGSTU22
MAEENKVILHKTYASSYAMRVELALKIKGIQFEYVDEDLSNKSPELLKYNPVHKKVPLLVHNGKPVSESLIIILEYIDETWKTGPLILPQDPYERARV
RFWANFIDQHLIPSTRKVWSTTGEEQEKAKEEIMEKLRILEEGLKTTNHENDNLGLLQILLVTLFGSYKVYEEVLGMKILVPENTPLLYSCVTSLNK
LPLVKEVCPPHDKMVALVNVYRQKLLKSSTK
>CaGSTU23
MGDVKLLGLWNSPFVHRVEWALKIKGVEYEFIEENLQNKSPLLLESNPIHKKIPVFFHNGKPICESMVIVEYIDEAFEGPSILPKDPYDRAIARFWA
KFLDDKLQAIAKTFFFKGEEQEKAKGEGYETLKVLDNELKDKKFFVADKFGFADIAANFVGLWLGVFEEATGIVLVTREKFPNFCAWRDEYINCTQN
KEYLPPRDELLAHFQVRFQAAAAASK
>CaGSTU24
MANDEVILLDFWPSMFGMRLRIALAEKEVKYEYKEEDLRNKSPLLLQMNPIHKKIPVLIHNGKPVCESNIAVEYIDEVWKDKAPLLPSDPYDRAQAR
FWADYIDKKMYETARKIYMVKGEEQEAGKKDFIEVLKVLEGALGDKPYFGGDNFGFVDIALIGFYCWFHAYETYGNFSIEAVCPKLVAWAKRCMQRD
SVAKSLPDQHKVLEFVKVIRQANGLE
>CaGSTU25
MAKDEVILLDFWASMFGMRLRIALAEKEVNYEYKEEEGLLNIKSPLLLQMNPIHKKVPVLIHNGKPICESIIGVEYIDEVWKDKAPLLPSDPYERAQ
ARFRADYIDKKLYDSARKIWGTKGEEPEAGKKDFIECLKVLEGELGDKPYFGGDNFGFVDIALIGFYSWFSAYETYGNFSTEAECPKFVAWAKRCMQ
RDSVAKSLPDQHKVLEFIKVLRKKYGVE
>CaGSTU26
MAKDEVILLDFWASMFGMRLRIALAEKQVNYEYKEEEGLLSNKSPLLLHMNPIHKKIPVLIHNGKPICESLIGVEYIDEVWKDIAPLLPSDPYERAQ
ARFWAYYIDKKEDMESKGRRAGGSWFYAYETYGNFSTEDECPKFVAWAKRCMQRNSVAKSLSDQNKILEVLRKKYGVE
>CaGSTU27
MANNEVILLDFWPSMFGMRLRVALAEKEIKYEYKEEDLLNTKSPLLLRMNPVHKKIPVLIHNGKSICESNIAVEYIDEVRKDKAPFLPCDPYERAQA
RFWADYIDKKHLDVIMYYLRKKYKNKNFPSKRYTTIDYFFKVYIDKAYVNYYDDDAGNELATQDASAKTDEVADMETTLINTIKGFSPRAAKKDFIE
VLKVLEGALGDKPYFGGDNFVFVDISLIGFYCWFYTYATYGNFSIEAECPKIVAWGKRCMQRDSVAKSLPNQHKVYEYVPVLKKKVWI
>CaGSTU28
MANDGVILLDFRPSMFGMRLRVALAEKEVKYEYKEEDLWNKSPLLLQMNPVHKKVPVLIHNGKSICESLIGVEYIDEVWKDKAPLLPCDPYERAQAR
FWADYIDKKMYGLGKKIWLTKGEKQEAIKKDFIECLKVLEGALGDKPYFGFVDISLIGYDCWFYTYETYGNFSIEAECPKFVAWAKRCMQRDSVAKS
LPDQYKVLKKKFGIE
>CaGSTU29
MAKDDEVILLGFWPSMFGVRPRIALDEKEVAYEYREEDFMGNNKSSLLLQMNPIHKKIPVLIHNGKPICESFIAVEYIDEVGKDKAPLLPSDPYERA
QARF
>CaGSTU30
MANDEVILLDFWPSMFGMRLRIALAEKEVKYEYKEEDLRNKSLLLLQMNPIHKKIPVLIHNGKPVCESNIAVEYIDEVWKDKAPLLPSDPYERAQAR
FWADYIDKKTVVLVNPHFIFQISLTSKLYHSSRKTWSTKEEDPEAAKKDFIEILKALEGVLGDRPYFGGDNFGFVDIALIGFYSWFYAYETYGGFST
ETECPKFVAWAKRCMQRDSVAKSLPDQHKVLEFAKAARQRLGIE
>CaGSTU31
MANDEVILLDFWPSMFGMRLRIALAEKEVKYESKEEDLRNKSPLLLQMNPVHKKIPVLIHNGKPICESIIAVEYIDEVWKDKAPLLPTDPYERAQAR
FWADYIDKKFFDSGRKIWTTKGEEQEAAKKDFIECLKVLEGALGEKPFFGGDNLGFVDVALIGFYSWFFAYETYGNFSTEAECPKFVTWAKRCMQRD
SVAKSLPDQHKVLEFVQTLRKKYGIE
>CaGSTU32
MSDQVVLLGTHVSMFAMRVKIALAEKGIQYEYKEENLMNKSPLLLQMNPIHKKIPVLIHNGKPVCESLIIVEYIDEVWKDKSPLLPSDPYKRAQARF
WADYVDKKIYDGGKKIWTTKEEDQEAANKEFIEYLKVLEGELGDKPYFGGESFGFVDMALIPYYSWFPAYEKFGKFSIEAVCPKFVAWAKRCMQKES
VSKSLYDPDKISDFVVMLRQRLGIA
>CaGSTU33
MRVRITLAEKGIQYEYKEQDLLNKSTLLLQMNPIHKKIPVLIHNGKPICESHIIVEYIDEVWKDKLPLLPSDPYERAHARFWTDYIGKKVYDGGMKI
WSSKVEVHETANKEFIECLKVLEGELGDKPYFNGENFGLVDMAFIPYYSWFPVYKKLSNLHIVEECPKFVSWAKRCMQKESVSKCLVDPDAIYDFIV
MFRQKTGVA
>CaGSTU34
MEERRDVKLLGTKESIFTQRIVWALKLKGIDYAFIEQDFSSRSSPLLVELNPVYKMVPVIVHDGKPLSESLVILEYIEETWPVNPLFPVDPFQRASA
RFWARFIDGMFYEAAKKAFFSSGEAKAEGVELVEEGLHLLEGQIIGKKFFGGEKIGYLDIIAGWISYWFQYIEEVGEFKAMDSRKYPCLHAWINNFI
QVPIIQQTLPKADDVKAVYRGFKDAAALAGAN
>CaGSTU35
LKLKGIDYAFIEQDLSSRNSPLFVELNPVYKKVPVIVHAGKSLCESLVILEYIEETWPYNPLFPIDPFQRASARFWARFIDGEFYEAAKKAFFSSGE
AKAEGVESVEEGLRLLEGQIIGKKFFGGEKIGYLDIVAGWISYWFQYIEEVGEFKAMDSRKYPCLHAWINNFIQVPIIQQSLAKPDDVKAVFRGFKD
AAALAGAN
>CaGSTU36

MNPIDKTIPVLIHDGKPICESLIIILQYIDETWPQNPILPNDPFQRASARCLAKFSEEKLNGPSRDAFYFTGEKQAKALEAMKEALGILESEISGKKF
FGGDEIGYVDIVVGWSSYWLQFVEIAANYTAMDSTKYPQMDRWMKIFLEVPLIKDNIPPYDGMLGIYQGYRKKSLLAFANDNNK
>CaGSTU37
MAELKLLGVSLSPFSRRVEWALKIKGVEYEFVEEELQNKSPLLLESNPIHKKIPVLIHNGKPICESMIIVEYIDETFEGPFILPKDPYDRAIARFWA
KFFDEKCMPVMGKALFGSGEESDKAKEELGELIKILENELKDKKFFVGDKFGFADMAGNLMAYWMAIVEEASGNVFVTSEKFPIFCAWRDEYANCSP
IKEHFPPREALLAHFNARFQAAAAASASK
>CaGSTU38
MAEIKLLGVSLSPFTHRVEWALKTKGVEYELIVEDTKNKSPLLLESNPIHKKIPVLIHNGKPICESMVIIEYIDETFEGPSILPKDPYERAIARFWA
KFLDDKCLPTMGKALLGEGEEQEKAKEECGELLKILDNELKHKKFFGGDQIGFADIAANALAFWMGIIEEASGVIWVTSEKFPNYCAWRENYNNCSQ
VKEYLPSRDELFTHFQSRFHSAAAPK
>CaGSTU39
MAEVQLLGYWASPFSKRVEIALKLKGIQYEYIEEDIPINKSPRVVKYNPIYKKIPVFLHDGKSIAESLLILEYINETWKEGTPLLPNDPYQRAMARF
WAKFMDEKCLPEMLKLCYESNYEVRMKAKGELHELLKLLENELIKDNTNLFGEYIEIVSILITYWIGIIQEAVQVDILKKEEFPNICGWADKLMSRS
FIKESRLPPRDKLLVFYKTYAKPLIKEVPQ
>CaGSTU40
MAEVKLLGLWYSPYTHRVEWALKIKGVNYEFIEQDPQNKSPLLLESNPVFKKIPVLIHNDKFICESMVIVEYIDDAFEGPPILPKDPYNRAIARFWA
KFFEDETEALGKSFLSKGEEKEKATEEAYEILKVLDNQLEDKKFFVGDKFGFADIAANGLGLYMGILEETTGIVLATREKFPNFCAWRDEYYTQNKE
YLHPRHEVLAKFKALFPAASVAK
>CaGSTU41
MAQVKLLGFWYSPFTHRVEWALKIKGVEYEYIEEDRNNKSSLLLESNPIHKKVPVLIHNGKPICESMVILEYIDETFEGPSILPKDPYDRALARFWA
KFLDEKVPAVVSTFLRVGEEREKGKEDVCEMLKVVDNELKDKKFFVGDKFGFVDMAANLIGLWLGIFQEASGVVLATSENFPNFCAWRDEYINCSQV
KEYLPPRREDLPAFYQARVQLAAVSASTKK
>CaGSTU42
MADVKLLGLWYSPYSNRVEWALKIKGEEYEYIEDDLHNKSPLLLELNPIHKKIPVLIHNGKPICESMVIVEYIDETFEGPPILPKDPYDRAIARFWA
KFFAEKEPATGKSFFLKGEEQEKANEETYEMLKVLDNELKDKKFFVGDSYGFADIAANMMGLWVGVFEEASGVLLATREKFPNFCAWRDEYINCSQN
KEYLPARDELTAYFKARFQAIKSAAK
>CaGSTU43
MVIVEYIDETFEGPSILPKDPYDRAVARFWVKFIDDKGSAVGRSFFHKSYEAKGELSEMLKILDNELKDKKFFAGDKFGFADIAANFLGLWMGVLEE
ATGVNNLVTREKYPNFCAWRDEYIDCSDNKKYLPPRDELLAYFKARFQAAAVPPYSS
>CaGSTU44
MGDVKLLGLWYSPFVHRVEWALKIKGVEYEFIEENLQNKSPLLLESNPIHKKIPVFFHNGKPICESMVIVEYIDEAFEGPSILPKDPYDRAIARFWA
KFLDDKLQAIAKTFFFKGEEQEKAKGEAYETLKVLDNEVKDKKFFVADKFGFADIAANFVGLWLGVFEEATGIVLVTREKFPNFCAWRDEYINCTQN
KEYLPPRDELLAHFQARFQAAAAASK
>CaGSTU45
MVGVKLLGSWYSLFTKRVEWALKIKGVKYEFIEEDLLNKSPLLIQSNPIHKKVPVLIHNGKHICESMVIVEYIDETFEGLSILPEDPYDRAIARFWA
KFLDDKWPAMIKSIFFKGEEQEKAKEEVYEMLKVLDNEFKDKKFFVGDNFGFADIVANAVALWLGVFEEAFGVVLVTSEKFPNFCAWRDEYYTQNKK
YLPSRDELISHYQTYIEAVAASK
>CaGSTU46
MAEVKLLGLWYSPYSNRVEWALKLKGVEYEFIEQDLQNKSPLLLESNPIHKKIPVLIHNGKPICESTVIVEYIDETFEGPPILPKDPYDRAIARFWA
KFFAEKGPAVGRSFFLKGEAQEKAKEEVYEMLKVVDNELKDKKFFVGDKFGFADIAANAAALWLGVLEEASGVVLVTSEMLPNFCAWRDQYINCSEN
KKYLPLRDELLAKLKARVQPAGAPK
>CaGSTU47
MTEVKLLGVGGSSYSRRVEWALKVKGVKYEFVEEDLQNKSPLLLESNPVLKKIPVLIHNGKPIRESMIILEYIDEAFEGPSILPKDPYERAIARFWA
KFLDGKCLDAVREALWSKFGGEQEKIKQEAYEMLKIVDNELKGKKFFGGNKIGFVDVAANYIPFWVEIVEEASGNVLITSEKFPNLCAWMDEYLSCS
EVKENLPDRDFMLSFFKAKALAENIIS
>CaGSTU48
MAGVKLLGVWYSPYSHRAEWALKLKGVEYELVEQDLQNKSPLLLESNPIHKKIPVLIHNGKPICESMIIVEYIDETFEGPSILPKDPYDRARARFWA
KFFDDKGDALTKSFFVQGEEQEKAKEQAYEMLKVLDNELKDKKYFVGDKFGFADIAATGVALYLGVLEEATGIVLATREKFPIFCAWRDEYCSENKE
YLPPRDGLLAAFKALFQAAAAAAAAK
> CaGSTU49
MFIDETFKGPSILPKDPYDRALAHFWAKFLEDKSTVAIDILLRVGEEQEKAKEEVYEMLKVLDNELKDKKFFVAENFGFAFDGTLARNFPTSLWSEK
FPNFCAWRDEYVNCSQVKEYLPPRKEDELLVYFQLAPARIEAAASA
>CaGSTU50
MAGEKVKFLRYWASPFALRVHWALKPKGIEYDYQEEHLPNKNPLLLQYNPVHKKILVLIHNGKPIAESLFILEYIEETWKHNPILPEDPYERAKVRF
WIKFVDDKVITN
>CaGSTU51
MAKNDLKILGAWPSPYVMRPRIALNIKSLAYDFLEEQFGTKSDLLLKSNPIYKKIPVLIHDGKPICESLIIVQYIDENWTNSGPSILPSHPYDRAIA
RFWATYIDDKWFPSLRGIAAAQGEDAKKAATELVVEGLVLLEDVFKNSSKGQKFFGGDTIGYLDIALGCFLGWLRVTEKMNNVTLLNESKTPGLYKW
AEDFCADSAVKDVMPETDKLAEAAKVIMSKMRAQASS
>CaGSTU52
MAGNDVKLLGSWPSTFVMRPRIALNIKSVGYDFLEEQLSSRSDVLLRSNPVYKKIPVLSIDETWTNSGPSILPSDPYDRAIARFWASYIDDKWFPLL
RVLAVAQGDHAIKAALEPLVKGLVLLEEVFKNCSKGKFFGGEKIGYLDIALGCFLGWMRVPQKMNNVKLLDEAKTPGLHKWAGDFCADSVVKDVMPQ
TDKLAEAAKVLLPKIRANASS
>CaGSTU53
MPAIFQVLELAMATSNVKLLGRNGCPFVNRAQMALNLKSIDHEFIQEDPFTKSKLLLKANPIDKKVPVLFHAETPISESLLIVQYIDEAWPNCLSIL
PSDPYERATTRFWAAYVDEKWFPLTAEYRKAQGEEAKAAVRDKVIEGTLLLEEAFIKCSKGKSFFGGDRIGYLDIVLGSLLGWLKVEEILTSTKIFD
ETKVPKLVAWTKRFCADKAVKDFIPV
>CaGSTU54
MATKGVKLLGVWASGYVNRVEMALKTKSIKYEFIQDNLLYKSELLLKANPVYKKVPILFHDEKPICESLVILQYIDEAWSNGPCILPSDPYDRAIAR
FWASYVDQKWFPLVLDLLNTEGKTAKAALLEKMSEASVLLEEAFIKISKGKSFFGGDRIGHLDIVLGSTLGWVKANEIMNEMKILDERKMPSLAEWD
ERFCSDKVLKDIIQQPEKLVEIFREFREIKKAASN

>CaGSTU55
MATGSVKLLGVWVSPFVILVENNPQDKVYWSEFIQETMLNKSELLLKSNPVYKKVPVMFHDEKPICESLVILLYIEEAWPNGPSILPFDPYDRAIAR
FWSDNIDEKVKAFFISDLMTVEFLTAKLKIKSIQSELIYETMLNKSELLLKSNAAYKKVQSCYTMKSLFVSPLSSFSTSMKFDRMVHLFFLLIPMIV
PLHDFGQPI
>CaGSTU56
MATGSVKLLGVWASPFVNRVEIALKMKSVEPEFILETTLNKSELLLKSNPVYKKIPVLLHDEKPICESLVILQYIDDSWPSGPSILPSDPYDRAIAQ
FWASYIDEKWYPLVVEFMKAEGKEAKAAVLEKISEGNLLLEEAFIKINKGKSFFGGDRIGYLDIVLGSLLGWVKAIEMMDEMKILDETKMPSLAEWD
KRFCSDNVVKDIIPQPEKLAEIHRKFHQMKKATSN
>CaGSTU57
MTTSSVKLLGVWASPYVNRVEIALKIKSIESEFIQENMLNKSELLLKSNPVYKKIPVLFHDEKPICESLVILQHIDEAWPSGPSILPSDPYDRAIAR
FWASYIDEKWYPLIVDFTNAEGKEAKAAVQEKMSEGNLLLEEAFIKISKGKSFFGGDRIGYLDIVLGSLLGWVKTIEMVYETKILDEIKMPSLAEWD
ERFCMDNVVKDIIPQPEKLVEVYRKYINRKKATSN
>CaGSTU58
MTTSSVKLLGVWASPYVNRVEIALKIKSIESEFIQENMLNKSELLLKSNPVYKKIPVLFHDEKPICESLVILQHIDEAWPSGPSILPSDPYDRAIAR
FWAYIDEKWYPLIVDFTNAEGKEAKAAVQEKMSEGNLLLEEAFIKISKGKSFFGGDRIGYLDIVLGSLLGWVKAIEMMNETKILDEIKMPSLAEWDG
RFCMDNVVKDIIPQPEKLVEVYRKYINRKKATSN
>CaGSTU59
MEKVKLIGTPFSFFTYRVIWALKLKGINYEYIDEDMSEKSSLLMKYNPIHKKVPVLIHGDKTICESMVIVEYIDDTWQLNPLLSTDPYYRAMARFWV
TYIEEKSTWNVFCNIGEEQQNAIKESLEMFKTIEENALGENNKLFGGENIGIVDIAFGGYAHWLEIIEEIVGVKLLDPHNFPKLNKWIKNFKQVQAI
KDNLPNHDEMLVYMKNAREKMLASP
>CaGSTF1
MVVKVHGSAMAACPQRVMVCLIELGVDFELIHVDLDSLEQKKPEFLLLQPFGHVPVIEEGDFRLFESRAIIRYYAAKYVDKGTKLTGTTLEEKALVD
QWLEVESNNFNDLVYNMVLQLLVFPKMGHKSDMTLVQKCADKLEKVFDIYEQRLSKSKYLAGDFFSLADLSHLPSLRFVMNDGGFAHLVTKRKCLQD
WYLDISSRPSWNKVLDLMIMKKSEMSPGPAKEAA
>CaGSTF2
MGIKIHGNPMSTATMRVVACVIEKDLDFEFVFVDMATDEHKKHPFLSLNPFGQVPAFADGDLKLFESRAITQYIAHVYASNGYQLILQDPKKMPIMS
VWMEVEGQKFEPLASKLTWELGIKPMIGMSTDDAIVKESEEQLSKVLDIYETRLAESKYLGGDSFTLVDLHHMPNIYYLMGTKVKALFDLRPRVSAW
CADILARPAWVKCLEKLQN
>CaGSTF3
MAIKVHGPIMSPAVMRVVAALKEKNLEFELVPVNMQNGDHKKEPFITLNPFGQVPAFEDGDLKLFESRAITQYIAHTYADKGNQLLPNDPKKMAIMS
VWMVEAQKFDPVGSKLAYEIAIKPMMGKVTDDAVVAENEEKMGKLLDVYEARLKESKYLGGDSFTLADLHHAPGLHYLSGTKVKSLFDARPHVTAWC
ADVLARPAWSKTLELNKQ
>CaGSTF4
MQLYHHPFSLDSQKVRLALEEKGIDYTSYHVNPLTGKNMDAFFFRKNPSGKIPVLQNGSHIIYDTTEIIQYIERIAETVSSGGNSTTLSSDVIEWTH
KLQECDSKHFTLFHIPAKYRLYVSKYLRHVIIARMAESPNLASDYHRKLKEAYTDDKMKNVEVLIQSKDHLVKLLDELEVKLGETSYLGGVEFTLAN
VMVIPVLARLELLKLEDEYINIRPNLADYWVVVKQRPSYKKVIGRYFDGWRKRKHY
>CaGSTF5
MEKLTKSFKSLSPDIHPEEEQEHQTLLNQTEKSNLSMQISKPNTELSPPSKPYFNKNDSNISTHQDFSFVTESPCSQHLLLSTSSFRKKSSLSMNRT
KSRLMNSQEQDQRSGAVLKDSEVEEEDPFSDEDLPEEYKKVKFSTLTVLQLVSLVSIIALLVCSFIFIILREKRAFGLELWKWEVMIIVLICGRLVS
GWGIRLVVFFIERNFLLRKRALYFVYGLRNSVQNCIWLSLLLIAWLCIFDKKVDKMTGGKVLPYVSSIWICLLVGAYIWLLKTLVVKVLAMSFHVST
FFDRIQESLFNQYVIEMWSGPPLVEIDQREHEEEEKVMSEVLADLKASTPHKTPTASTPRSSAFSMVISEKEKEKEGGITIDHLHRLNQKNISAWNM
KRLMNMVRKGVLSTLDEKLPQPNYEDESAVQITSGKKAKAAAKKIFNSVAKPGSKFIYLEDLRFMRQDEAVKTMCLIEGGTETCGISKCALKNWVPF
GQVPAFQDEDITLFESRSINRYICDKYGSQGNKGLYGTNPLEKASIDQWIEAEGQSFNPPSSILVFQLAFAPRMKLKQDENLIRQNEEKLKKVLDVY
EKRLGESQYLAGDEFTLADLSHFPNIQYLVNGTDRAELFTSRENMGRWWGEISSRESWKKVVEMQNSPPPS
>CaGSTF6
MTTPVKVYGPTLSTAVSRVLACFLEKDVKFELIPVNMAKGEHKKPDFQNSGKFLPFGQVPAFQDEDITLFVFVLDAHTSSELGSQNEEKLKKVLDVY
EKRLGESQYLAGDEFTLADLSHFPNIQYLVNGTDRAELFTSRENVGRWWGEISSRESWRKDVFVDCTILLLAWSEFFDGAHFHRHEHTRCGCHTLNM
TEWAIKLCIWSLSLGFDISPIRAQSSSSDHWVDSWIIVTSDSGPVDGRLLQSELQIPV
>CaGSTT1
MQIPAIMDGKFKLSESHAIIRYLASVFPIADHWYPTDLYKRAKIESVLDWHRTNFPRGPVGMPLNPQAAAKAEKVLIASLAQIESVWLQKKGRFLLG
SGQPSVADLSLACEIMQLEVLDDKVRERILGPFKRVTKWLDDTKHVMAPHFEEVHSILYKVKKKRQKQTSPVGSSSTQSSRKHDLHSKM
>CaGSTT2
MTLKLYVDRMSQACREVIIFCKVNGIDFEEVHIDLSKRQQLSPEYREINPIQQIPAIVDGSFKLSESHAILRYLTCAYPGIADHWYPADLYKRAKVE
SMLDWHRTTFPRGPGSYFFYAVFASAVGRPLNAKAAARTEKNLIAALAKIESVWLQKKGRFLLGSNQPSIADLSLACEVMQLEGLDEKEHERILGPF
KRVLKWLDDTKNAMAPHFEEVQSTVSKAKEKLQKQRNAAGSNTTQSGRKPVLHSKI
>CaGSTT3
MSQPSRAVIIFCKLNGIDFEEVRINLSKREQLSPEFKEINPMKQVPAIMDGRFKLSESHAILKYLACAFPGIADHWYPADLYKRAKVDSVLDWHHSN
LRRGAAAYVFSTVLAPSFGLTLNPQAAAEAEKVLLTSLAKIESVWLQKKGQFLLGSGQPSIADLSLVCELMQLEVLDEKDRERIIGPYKRVLKWIDD
TKNAMQPHFQEVHVILFKAKERFHKQRSAVGSSLPQSSRKPDLHSKM
>CaGSTT4
MSLKLYVDRLSQPSRALLIFCKLNGIEFEEVNIELSKGQHRTPEYQEVNIMKQVPAIVHGDFKLFESHAILRYLASAFPEVADHWYPSDVKKRASVE
CVLDWHHANLRRGSAGYVFNSVLAPAFGLPLNPQAAAEGKKLLSASLATIDTYWLQKDGRFLLGNSQPSLADLSLVCEIMQLEFLNEEDRGSIFSPH
KNVLKWIDDVKSATTPYFDEMHATLFKVKEVTKQRAAGPSS
>CaGSTZ1
MKIKPAMLLQISKSYNKLAKADNYPRWNYFQRLFGRHFSQTETPTTNSILNASSMESHNSQKKATDSTWVSKIVLYSFWQSSCSWRVRFALNLKGLS
YEYRAVNIDKGEQFTSEFDKLNPLHYVPVLVDGDVVISDSYAILLYLEEKYHQRPLLPVEPQLRALNLQAASIVSSNMQPFHMLSVLRYMEERVGPE
EKQLWVNFHIQKGFGAALEKLLTESAGKYATGEEVYMADVFLAPQIAVATKRFNVDMSEFPTLRKIYNSCEALPEFKASLPERQPDAAL
>CaGSTZ2
MYGIEYLKLNPLAYVPTLVDGDAVIVDSFAIIMYLEEKYPQRALLPQDHQKKAINYQVEELFSAGFYYFFSRQKVIAHHIFEYTEHVLISNYSSSSA
ESVGYEILFF
>CaGSTL1

MAASSIGYQIHINFNSPIIFPSKTKLSSLSFSFSNAKYALKWNHIGCHRICALPVVSIMASRSSTEVLPPALDSSSEPPTIFDGTPKLYISYSCPYA
QRTWIARNCKGLQEKIKLVPIDLKNRPDWYKEKVYPANKFPGRGQRIKLVRSCCFRTVPSLEHNDEVKGESMDLIRYIDSNFEGPSLFPDDPYKREF
AEELFSYFDPFYKAVISSLKEDRIDNATFDSIETALSKFDDGSFFLGTFSLVDIAYAPFIERFQPFLLEVKNYDITAGRIKLAAWIKVMGRGGGRTP
VTGVTSVLPQDTALPLTTRDLRC
>CaGSTL2
MAAPNVQEVLPPSLDSTSQPPSLFDGTTRLYINYQCPYAQRAWITRNFKGLQDKIELVPIDLQNRPAWYKEKVYPPNKVPSLEHNNKVIGESLDLIK
YIDSNFEGPSLLPDDPEKRKFAEELIAYSDTFVPEVYGSFKKDVQTLAGAQFDYLEKALHKFDYGPFFLGQFSQVDIAYVPFIERFQIFLQEVFNYD
ITSGRPKLAKWIEEVNKLDGYKQTKVLDPKRLVEYYKNRFLVATL
>CaGSTL3
MSRPGHLAMDMDMGIWLYISYICPFAQRAWITRNVKGLQDKIELVPIDLQNRPAWYKEKVYPQNKVPALEHNNKVIGESLDLIKYIDSNFEGPSLLP
DDPEKQKFAEELVAYTDTFLKEIYGSFKGDVEKQAGPQFDYLEKALEKFDNGPFFLGQFSQADIAYASFIERFQIFLQEVFNYDITSGRPKLAKWIE
ELNKIDGYIQTKADPKKVVEIYKSRFMA
>CaGSTL4
MATPSAQEIRPPSLISSSEPPSLFDGTTRLYISYICPFAQRAWITRNVKGLQDKIELVPIDLQNRPAWYKEKVYAQNKVPALEHNNKVIGESLDLIK
YIDSNFEGPSLLPDDPEKQKFAEELVTYTDTFLKEIYGSFKGDVEKQAGPHFDYLEKALEKFNNGPFFLGQFSQADIAYASFIERFQIFLQEVFNYD
ITSWRPKLAKWIEELNKIDGYIQTKADPKKVVEIYKSRFMIVLRPVACAGNLFQHSNSC
>CaEF1Bg1
MALILHSTSNNKNAAKGLIAAEYTGVKVELAKDFQMGVSNKTPEFLEMNPIGKVPVLQTPDGPVFESNAIARYVTKTKPDNLLFGSSLIEYAQIEQW
NDFSATEVDANIARWLYPRLGYAVYIPPAEEAAVAALKRALGALNTHLASNTYLVGHSITLADIIMVCNLSIGFGVIMTKSFTKEFPHVERYFWTVV
NQPNFCKILGEVKQAESIPAPPSKKPAPATEKPKAKEEPKKEVKEEPKPAEEEAAPKPKPKNPLDLLPPSPMILDDWKRLYSNTKTNFREVAVKGFW
DMYDPEGYSLWFCDYKYNDENTVSFVTLNKVGGFLQRMDLVRKYAFGKMLIVGSEPPFKVQGLWLFRGKEIPKFVMDEVYDMELYEWKQVDINDEAQ
KERVSQMIEDHEPFEGQALLDAKCFK
>CaEF1Bg2
MALILHSTSNNKNAAKGLIAAEYTGVRDELTKDFQMGVSNKTPEFLEMNPIRKVSVLQTPDGPVFESNAIARMIMTKSFTKEFPHVERYFWTVVNQP
NFCKMVKQAESIPAPQSKKPTQATEKPKAKEEPKKEVKEEPKPAEEEAARKPKPKNPLDLLPPSLMILDDWKRLYSNTKTNFREVDVKGFWDMYDPE
GYSLWFCDYK
>CaDHAR1
MSTVKITPSAASLTTTIKHLASIHNTTIFTSNSTKFRAPRRGFTVSMAAFVHTPLEVCVKQSITIPNKLGDLSVGTMLAQSTMLTPKLMGLGFECSR
AVNQAGPFTQRVLLTLEEKHLPYDMKFVDLSNKPDWFLKISPEGKVPLIKLDEWVPDSDVITQALEEKYPDPPLTTPPEKASVGSKIFPKFVAFLKS
KDSSDGTEQALLDELTAFNDYLKDNGPLINGNEVSAADLSLGPKLYHLEIALGYYKNWSIPDSLSYVKSYMKSIFSRETFINTRALKEDVIEGWRPK
VMG
>CaDHAR2
MGVEICVKAAVGAPNVLGDCPFSQRALLTLEEKKVPYKMILINVSDKPKWFLEVNPEGKVPVINFGDKWIPDSDVIVALLEEKYPNPSLVTPPEFAS
VGSKIFPTFVSFLKSKDSSDGTEQALLDELNALEEHLKAHGPYVNGQNICSVDLSLAPKLYHLQVALGHFKKWSVPESLSHVHNYMKLLFERESFQK
TKAAEEYVIAGWAPKVNP
>CaTCHQD
MQLYHHPFSLDSQKVRLTLEEKGIDYTSHHVNPLTGKNMDAFFFNMNPSAKVPVFQNGSHIIYDTIEIIQYIERIAEKVSSGGNNLNPSSREVVRWM
HKIQEWDAMYLTLFHVPEKYRLYVSKFLRRVIIARMAECPDLAGAYHCKLREAYDIDDKLKNAEVSRRSENHLVRLLDEVELKLGETSYLAGEEFTL
ADVMLIPVLARIELLNLEEEYINSRPNIADYWVLAKQRPSYKKVIGKYFDGWRRRKTLLKTWCFIRIRSMLRKF
>CaMGST1
MAGVELLPREYGYVVLALVVYCFLNFWMSFQVGKARKKYKVTYPTMYAIESENKDAKFFNCVQRGHQNSIEAMPMFFMLMIVGGIRHPLICSSLGAV
YIVSRYFYFTGYSTGDPQNRLKIGGFNFLAIMGLMICSISCGVNFLM
>CaMGST2
MRRVGRIAAVYRAVDGASAVSAPQHRMSTAAHFSSSSNKSYAKSNWLFNNLLTDLSARTAAAHAVAGTMLFSVAATTLTEEVHAKVAVPPELRPKDL
VLYQYEACPFCNKVKAFLDYYDLPYKVIEVNPISKKELKWSDYKKVPVVVVDGEQMVNSSDIIDQLYKKVRSADSTFDANEESKWRKWVDDHLVHML
SPNIYRSTSEALESFDYITTHGNFSFTERITAKYAGAAAMYFVSKKLKKKYNITDERAALYEAAETWVDALKGRDFLGGSNPNLADLAVFGVLRPIR
YLKSGKDMVENTRIGDWYSRMESAVGSARIQA
>CaGHR1
MYLSSMNFPFINRSIQAQTLTHTCGLQQLSFKHSIQMARSALDEMSATGAFERTASTFRNIISRDPGSRFPVESGRYHLYISYACPWASRCLAYLKI
KGLDQAIDFTSVKPIWERTKDTDEHMGWVFASSSTKEPGADPDPVNGAKSIRELYELSSTNYSGKYTVPVLWDKKLKTVVNNESSEIIRMFNSEFND
IAGNAALDLYPSHLQSQINETNDWIYNGINNGVYRCGFAKKQKPYEEAVQELYEALDKCEEILSKQRYLCGDQVTEADIRLFVTLIRFDEVYVVHFK
CNKKLLREYPNLFNYTKDIFQIPGMSSTVNMEHIKKHYYGSHPTINPFGIIPQGPNIDYSSPHDREKFSK
>CaGHR2
MKTVVNNGSSEIIRMFNSEFNDITGNAALDLYPPHLQSQSNETNDWIYNEINNGVNRCGFAKKQEPYDEAVQELYEALDKCEEILSKQSYLGGDQVS
KADIHLFMTLIRLDEDLTTGTIGGAA
>CaGHR3
MSLNQNPNTTSTANLINTVTKLLWGQSLPPQLLISTVRSSWSATWQLMMSQLAPSDPSGSYTRPASQFRLYESNSPPNNLHLYVGLPCPWAHRTLMV
RALKGLETSIPVSIASPGIDGSWEFRVWPDPAKEKIVPGLDIANGCKTLREVYNLRRGGYSGRSTVPMLWDVEKKEVLCNESYDIIEFFNSGLNGIA
GNPELNLSPPALKEEIRKWNDIIYPNINNGVYRCGFAQSQEAYNKAAEVLFRTLEMLEDHLGGSRYLCGDVLTLADVCLFTTLIRFDLVYNVLFKCT
KRKLIEFTNLHGYLRDIYQIPKVAETCNMGQIMEGYYKILFPLNPGGINPIMPSGCEDEVLSKPHNRDSLSLDSKVVQHSVS
>ATGSTF7
MAGIKVFGHPASTATRRVLIALHEKNLDFEFVHIELKDGEHKKEPFIFRNPFGKVPAFEDGDFKLFESRAITQYIAHFYSDKGNQLVSLGSKDIAGI
AMGIEIESHEFDPVGSKLVWEQVLKPLYGMTTDKTVVEEEEAKLAKVLDVYEHRLGESKYLASDKFTLVDLHTIPVIQYLLGTPTKKLFDERPHVSA
WVADITSRPSAKKVL
>ATGSTF6
MAGIKVFGHPASTATRRVLIALHEKNVDFEFVHVELKDGEHKKEPFILRNPFGKVPAFEDGDFKIFESRAITQYIAHEFSDKGNNLLSTGKDMAIIA
MGIEIESHEFDPVGSKLVWEQVLKPLYGMTTDKTVVEEEEAKLAKVLDVYEHRLGESKYLASDHFTLVDLHTIPVIQYLLGTPTKKLFDERPHVSAW
VADITSRPSAQKVL
>ATGSTF5

MGINASHVPETCYHHCNQTFESSRQCFKWCQELARKDEYKIYGYPYSTNTRRVLAVLHEKGLSYDPITVNLIAGDQKKPSFLAINPFGQVPVFLDGG
LKLTESRAISEYIATVHKSRGTQLLNYKSYKTMGTQRMWMAIESFEFDPLTSTLTWEQSIKPMYGLKTDYKVVNETEAKLEKVLDIYEERLKNSSFL
ASNSFTMADLYHLPNIQYLMDTHTKRMFVNRPSVRRWVAEITARPAWKRACDVKAWYHKKKN
>ATGSTF4
MDCLQMVFKLFPNWKREAEVKKLVAGYKVHGDPFSTNTRRVLAVLHEKRLSYEPITVKLQTGEHKTEPFLSLNPFGQVPVFEDGSVKLYESRAITQY
IAYVHSSRGTQLLNLRSHETMATLTMWMEIEAHQFDPPASKLTWEQVIKPIYGLETDQTIVKENEAILEKVLNIYEKRLEESRFLACNSFTLVDLHH
LPNIQYLLGTPTKKLFEKRSKVRKWVDEITSREAWKMACDQEKSWFNKPRN
>ATGSTU18
MATEDVKLIGSWASVYVMRARIALHLKSISYEFLQETYGSKSELLLKSNPVHKKMPVLIHADKPVCESNIIVHYIDEAWNSSGPSILPSHPYDRAIA
RFWAAYIDDQWFISVRSILTAQGDEEKKAAIAQVEERTKLLEKAFNDCSQGKPFFNGDHIGYLDIALGSFLGWWRVVELDANHKFLDETKTPSLVKW
AERFCDDPAVKPIMPEITKLAEFARKLFPKRQA
>ATGSTU17
MASSDVKLIGAWASPFVMRPRIALNLKSVPYEFLQETFGSKSELLLKSNPVHKKIPVLLHADKPVSESNIIVEYIDDTWSSSGPSILPSDPYDRAMA
RFWAAYIDEKWFVALRGFLKAGGEEEKKAVIAQLEEGNAFLEKAFIDCSKGKPFFNGDNIGYLDIALGCFLAWLRVTELAVSYKILDEAKTPSLSKW
AENFCNDPAVKPVMPETAKLAEFAKKIFPKPQA
>ATGSTU24
MADEVILLDFWASMFGMRTRIALAEKRVKYDHREEDLWNKSSLLLEMNPVHKKIPVLIHNGKPVCESLIQIEYIDETWPDNNPLLPSDPYKRAHAKF
WADFIDKKVNVTARRIWAVKGEEQEAAKELIEILKTLESELGDKKYFGDETFGYVDIALIGFHSWFAVYEKFGNVSIESECSKLVAWAKRCLERESV
AKALPESEKVITFISERRKKLGLE
>ATGSTU25
MADEVILLDFWPSMFGMRTRIALEEKNVKFDYREQDLWNKSPILLEMNPVHKKIPVLIHNGNPVCESLIQIEYIDEVWPSKTPLLPSDPYQRAQAKF
WGDFIDKKVYASARLIWGAKGEEHEAGKKEFIEILKTLESELGDKTYFGGETFGYVDIALIGFYSWFEAYEKFGSFSIEAECPKLIAWGKRCVERES
VAKSLPDSEKIIKFVPELRKKLGIEIE
>ATGSTU26,
MANDQVILLDYWPSMFGMRTKMALAEKGVKYEYKETDPWVKTPLLIEMNPIHKKIPVLIHNGKPICESLIQLEYIDEVWSDASPILPSDPYQKSRAR
FWAEFIDKKFYDPSWKVWATMGEEHAAVKKELLEHFKTLETELGDKPYYGGEVFGYLDIALMGYYSWFKAMEKFGEFSIETEFPILTTWTKRCLERE
SVVKALADSDRIIEYVYVLRKKFGAA
>ATDHAR1
MALEICVKAAVGAPDHLGDCPFSQRALLTLEEKSLTYKIHLINLSDKPQWFLDISPQGKVPVLKIDDKWVTDSDVIVGILEEKYPDPPLKTPAEFAS
VGSNIFGTFGTFLKSKDSNDGSEHALLVELEALENHLKSHDGPFIAGERVSAVDLSLAPKLYHLQVALGHFKSWSVPESFPHVHNYMKTLFSLDSFE
KTKTEEKYVISGWAPKVNP
>ATGSTU13
MAQNDTVKLIGSWSSPYSLRARVALHLKSVKYEYLDEPDVLKEKSELLLKSNPIHKKVPVLLHGDLSISESLNVVQYVDEAWPSVPSILPSDAYDRA
SARFWAQYIDDKCFAAVDAVVGAKDDEGKMAAVGKLMECLAILEETFQKSSKGLGFFGGETIGYLDIACSALLGPISVIEAFSGVKFLRQETTPGLI
KWAERFRAHEAVKPYMPTVEEVVAFAKQKFNVQ
>ATGSTU14
MAQNDTVKLIGCSDDPFSIRPRVALHLKSIKYEYLEEPDDDLGEKSQLLLKSNPIHKKTPVLIHGDLAICESLNIVQYLDEAWPSDPSILPSNAYDR
ASARFWAQYIDDKCFEAANALTGANNDEERIAATGKLTECLAILEETFQKSSKGLGFFGGETIGYLDIACAALLGPISVIEMFSADKFVREETTPGL
IQWAVRFRAHEAVRPYMPTVEEVTELVKQRIEEGFKRNFKSNVSTSEYE
>ATGSTF14
MADSKMKLHCGFIWGNSAALFCINEKGLDFELVFVDWLAGEAKTKTFLSTLNPFGEVPVLEDGDLKLFEPKAITRYLAEQYKDVGTNLLPDDPKKRA
IMSMWMEVDSNQFLPIASTLIKELIINPYQGLATDDTAVQENKEKLSEVLNIYETRLGESPYLAGESFSLADLHHLAPIDYLLNTDEEELKNLIYSR
PNVAAWVEKMKMRPAWLKTVVMKNHIVDLMKQRRLPIKLDSSCHESTVVAQKNAIAIENK
>ATGSTU28
MGKENSKVVVLDFWASPYAMRTKVALREKGVEFEVQEEDLWNKSELLLKSNPVHKKVPVLIHNNTPISESLIQVQYIDETWTDAASFLPSDPQSRAT
ARFWADYADKTISFEGGRKIWGNKKGEEQEKGKKEFLESLKVLEAELGDKSYFGGETFGYVDITLVPFYSWFYALEKCGDFSVEAECPKIVAWGKRC
VERNSVAATLPESEKVYQQVLKLRQIFGVE
>ATGSTU15
MGEREEVKLLGTWYSPVVIRAKIALRLKSVDYDYVEEDLFGSKSELLLKSNPIFKKVPVLIHNTKPVCVSLNIVEYIDETWNSSGSSILPSHPYDRA
LARFWSVFVDDKWLPTLMAAVVAKSEEAKAKGMEEVEEGLLQLEAAFIALSKGKSFFGGETIGFIDICLGSFLVLLKAREKLKNEKILDELKTPSLY
RWANQFLSNEMVKNVVPDIDKVAKFIEEFEDRAQYIRCF
>ATGSTU16
MGEKEEVKLLGVWYSPYAIRPKIALRLKSVDYDYVEENLFGSKSELLLKSNPVHKKVPVLLHNNKPIVESLNIVEYIDETWNSSAPSILPSHPYDRA
LARFWSDFVDNKWFPALRMAAITKSEDAKAKAMEEVEEGLLQLEDAFVSISKGKPFFGGEAIGFMDICFGSFVVLLKAREKFKAEKLLDESKTPSLC
KWADRFLSDETVKNVAPEIEKVAEFLQELEVRAQSAASRS
>ATGSTU12
MLKNKKSDNSLSRDTLQIKKRKKTTMAQNGSNTTVKLIGTWASPFAIRAQVALHLKSVEHEYVEETDVLKGKSDLLIKSNPIHKKVPVLIHGDVSIC
ESLNIVQYVDESWPSDLSILPTLPSERAFARFWAHFVDGKLFESIDAVAGAKDDAARMTLAGNLMENLAALEEAFQKSSKGGDFFGGGNIGFVDITV
GAIVGPISVIEAFSGVKFLRPDTTPGLIQWAEKFRAHEAVKPYMPTVAEFIEFAKKKFSV
>ATGSTU11
MGLMNRSKNDEYVKLLGAWPSPFVLRTRIALNLKNVAYEYLEEEDTLSSESVLNYNPVHKQIPILIHGNKPIRESLNIVMYVDETWLSGPPILPSDP
FDRAVARFWDVYIDEHCFTSINGVAVAKGEENINAAIAKLEQCMALLEETFQECSKGRGFFGGENIGFIDIGFGSMLGPLTVLEKFTGVKFIHPENT
PGLFHWADRFYAHEAVKPVMPDIEKLVQFARLKFNTSIFK
>ATGSTU10
MEEKKSKVILHGTWISTYSKRVEIALKLKGVLYEYLEEDLQNKSESLIQLNPVHKKIPVLVHDGKPVAESLVILEYIDETWTNSPRFFPEDPYERAQ
VRFWVSYINQQVFEVMGQVMSQEGEAQAKSVEEARKRFKVLDEGLKKHFPNKNIRRNDDVGLLEITIIATLGGYKAHREAIGVDIIGPVNTPTLYNW
IERLQDLSVIKEVEVPHDTLVTFIQKYRQKCLQQAANA
>AtDHAR2
MALDICVKVAVGAPDVLGDCPFSQRVLLTLEEKKLPYKTHLINVSDKPQWFLDISPEGKVPVVKLDGKWVADSDVIVGLLEEKYPEPSLKTPPEFAS
VGSKIFGAFVTFLKSKDANDGSEKALVDELEALENHLKTHSGPFVAGEKITAVDLSLAPKLYHLEVALGHYKNWSVPESLTSVRNYAKALFSRESFE
NTKAKKEIVVAGWESKVNA

>AtTCHQD1
MQLYHHPYSIDSQRVRLALEEKGIDYTSYHVNPITGKHMDPSFFRMNPNAKLPVFRNGSHIILDTIEIIEYLERIAEVSSGIEDATFNREVVEWMRK
IREWESKLFTLAHIPDNRRLYVSKFLRMVVIARMAESPDLASAYHRKLREAYDTEDKLKDPGALRRSKDHLLRLLDEVETKLEGTTYLAGNEFSMAD
VMLIPVLARLSLLDLEEEYISSRKNLAEYWALVRRRPSYKKVIGRYFNGWRKYATLVKTWMFVRVRSLLRKY
>AtGSTU23
MEEEIILLDYWASMYGMRTRIALEEKKVKYEYREEDLSNKSPLLLQMNPIHKKIPVLIHEGKPICESIIQVQYIDELWPDTNPILPSDPYQRAQARF
WADYIDKKTYVPCKALWSESGEKQEAAKIEFIEVLKTLDSELGDKYYFGGNEFGLVDIAFIGFYSWFRTYEEVANLSIVLEFPKLMAWAQRCLKRES
VAKALPDSDKVLKSVSDHRKIILGID
>AtGSTU22
MADEVILLDFWPSPFGVRARIALREKGVEFEYREENLRDKSPLLLQMNPVHKKIPVLIHNGKPVCESMNVVQYIDEVWSDKNPILPSDPYQRAQARF
WVDFVDTKLFEPADKIWQTKGEEQETAKKEYIEALKILETELGDKPYFGGDTFGFVDIAMTGYYSWFEASEKLANFSIEPECPTLMASAKRCLQRES
VVQSLHDSEKILAFAYKIRKIYCV
>AtGSTU21
MAAEVILLGFWPSMFGMRTMIALEEKGVKYEYREEDVINNKSPLLLEMNPIHKTIPVLIHNGKPVLESLIQIQYIDEVWSDNNSFLPSDPYHRAQAL
FWADFIDKKEQLYVCGRKTWATKGEELEAANKEFIEILKTLQCELGEKPYFGGDKFGFVDIVLIGFYSWFPAYQKFGNFSIEPECLKLIAWGKRCMQ
RESVAKALPDSEKVVGYVLQLKKLYGIE
>AtGSTU20
MANLPILLDYWPSMFGMRARVALREKGVEFEYREEDFSNKSPLLLQSNPIHKKIPVLVHNGKPVCESLNVVQYVDEAWPEKNPFFPSDPYGRAQARF
WADFVDKKFTDAQFKVWGKKGEEQEAGKKEFIEAVKILESELGDKPYFGGDSFGYVDISLITFSSWFQAYEKFGNFSIESESPKLIAWAKRCMEKES
VSKSLPDSEKIVAYAAEYRKNNL
>AtGSTU19
MANEVILLDFWPSMFGMRTRIALREKGVEFEYREEDLRNKSPLLLQMNPIHKKIPVLIHNGKPVNESIIQVQYIDEVWSHKNPILPSDPYLRAQARF
WADFIDKKLYDAQRKVWATKGEEQEAGKKDFIEILKTLESELGDKPYFSGDDFGYVDIALIGFYTWFPAYEKFANFSIESEVPKLIAWVKKCLQRES
VAKSLPDPEKVTEFVSELRKKFVPE
>AtGSTZ2
MSYVTDFYQAKLKLYSYWRSSCAHRVRIALTLKGLDYEYIPVNLLKGDQSDSDFKKINPMGTVPALVDGDVVINDSFAIIMYLDDKYPEPPLLPSDY
HKRAVNYQATSIVMSGIQPHQNMALFRYLEDKINAEEKTAWITNAITKGFTALEKLLVSCAGKYATGDEVYLADLFLAPQIHAAFNRFHINMEPFPT
LARFYESYNELPAFQNAVPEKQPDTPSTI
>AtGSTZ1
MANSGEEKLKLYSYWRSSCAHRVRIALALKGLDYEYIPVNLLKGDQFDSVYRFDLQDFKKINPMGTVPALVDGDVVINDSFAIIMYLDEKYPEPPLL
PRDLHKRAVNYQAMSIVLSGIQPHQNLAVIRYIEEKINVEEKTAWVNNAITKGFTALEKLLVNCAGKHATGDEIYLADLFLAPQIHGAINRFQINME
PYPTLAKCYESYNELPAFQNALPEKQPDAPSSTI
>AtGSTF3
MAGIKVFGHPASTSTRRVLIALHEKNLDFELVHVELKDGEHKKEPFLSRNPFGQVPAFEDGDLKLFESRAITQYIAHRYENQGTNLLPADSKNIAQY
AIMSIGIQVEAHQFDPVASKLAWEQVFKFNYGLNTDQAVVAEEEAKLAKVLDVYEARLKEFKYLAGETFTLTDLHHIPVIQYLLGTPTKKLFTERPR
VNEWVAEITKRPASEKVL
>AtGSTU7
MAERSNSEEVKLLGMWASPFSRRIEIALTLKGVSYEFLEQDITNKSSLLLQLNPVHKMIPVLVHNGKPISESLVILEYIDETWRDNPILPQDPYERT
MARFWSKFVDEQIYVTAMKVVGKTGKERDAVVEATRDLLMFLEKELVGKDFLGGKSLGFVDIVATLVAFWLMRTEEIVGVKVVPVEKFPEIHRWVKN
LLGNDVIKKCIPPEDEHLKYIRARMEKLNIKSA
>AtGSTU6
MGKNEEVKLLGIWASPFSRRIEMALKLKGVPYEYLEEDLENKSSLLLALSPIHKKIPVLVHNGKTIIESHVILEYIDETWKHNPILPQDPFQRSKAR
VLAKLVDEKIVNVGFASLAKTEKGREVLIEQTRELIMCLEKELAGKDYFGGKTVGFLDFVAGSMIPFCLERAWEGMGVEMITEKKFPEYNKWVKKLK
EVEIVVDCIPLREKHIEHMNNMAEKIRSA
>AtGSTU5
MAEKEEVKLLGIWASPFSRRVEMALKLKGIPYEYVEEILENKSPLLLALNPIHKKVPVLVHNGKTILESHVILEYIDETWPQNPILPQDPYERSKAR
FFAKLVDEQIMNVGFISMARADEKGREVLAEQVRELIMYLEKELVGKDYFGGKTVGFLDFVAGSLIPFCLERGWEGIGLEVITEEKFPEFKRWVRNL
EKVEIVKDCVPPREEHVEHMNYMAERVRSS
>AtGSTU4
MAEKEEDVKLLGFWASPFTRRVEMAFKLKGVPYEYLEQDIVNKSPLLLQINPVYKKVPVLVYKGKILSESHVILEYIDQIWKNNPILPQDPYEKAMA
LFWAKFVDEQVGPVAFMSVAKAEKGVEVAIKEAQELFMFLEKEVTGKDFFGGKTIGFLDLVAGSMIPFCLARGWEGMGIDMIPEEKFPELNRWIKNL
KEIEIVRECIPPREEQIEHMKKVVERIKSA
>AtGSTU3
MAEKEEGVKLIGSWASPFSRRVEMALKLKGVPYDYLDEDYLVVKSPLLLQLNPVYKKVPVLVHNGKILPESQLILEYIDQTWTNNPILPQSPYDKAM
ARFWAKFVDEQVTMIGLRSLVKSEKRIDVAIEEVQELIMLLENQITGKKLFGGETIGFLDMVVGSMIPFCLARAWEGMGIDMIPEEKFPELNRWIKN
LKEIEIVRECIPDREKHIEHMMKIVGRIKAV
>AtGSTU2
MAKKEESVKLLGFWISPFSRRVEMALKLKGVPYEYLEEDLPKKSTLLLELNPVHKKVPVLVHNDKLLSESHVILEYIDQTWNNNPILPHDPYEKAMV
RFWAKFVDEQILPVGFMPLVKAEKGIDVAIEEIREMLMFLEKEVTGKDFFGGKTIGFLDMVAGSMIPFCLARAWECLGIDMTPEDTFPELNRWIKNL
NEVEIVRECIPPKEKHIERMKKIIERAKSTF
>AtGSTU1
MAEKEESVKLLGFWASPFSRRVEMALKLKGVPYEYLEEDLPNKTPLLLELNPLHKKVPVLVHNDKILLESHLILEYIDQTWKNSPILPQDPYEKAMA
RFWAKFIDDQILTLGFRSLVKAEKGREVAIEETRELLMFLEKEVTGKDFFGGKTIGFLDMIAGSMIPFCLARLWKGIGIDMIPEEKFPELNRWIKNL
EEVEAVRGCIPPREKQIERMTKIAETIKSA
>AtGSTF9
MVLKVYGPHFASPKRALVTLIEKGVAFETIPVDLMKGEHKQPAYLALQPFGTVPAVVDGDYKIFESRAVMRYVAEKYRSQGPDLLGKTVEDRGQVEQ
WLDVEATTYHPPLLNLTLHIMFASVMGFPSDEKLIKESEEKLAGVLDVYEAHLSKSKYLAGDFVSLADLAHLPFTDYLVGPIGKAYMIKDRKHVSAW
WDDISSRPAWKETVAKYSFPA
>AtGSTF10

MVLTIYAPLFASSKRAVVTLVEKGVSFETVNVDLMKGEQRQPEYLAIQPFGKIPVLVDGDYKIFESRAIMRYIAEKYRSQGPDLLGKTIEERGQVEQ
WLDVEATSYHPPLLALTLNIVFAPLMGFPADEKVIKESEEKLAEVLDVYEAQLSKNEYLAGDFVSLADLAHLPFTEYLVGPIGKAHLIKDRKHVSAW
WDKISSRAAWKEVSAKYSLPV
>AtGSTF8
MGAIQARLPLFLSPPSIKHHTFLHSSSSNSNFKIRSNKSSSSSSSSIIMASIKVHGVPMSTATMRVLATLYEKDLQFELIPVDMRAGAHKQEAHLAL
NPFGQIPALEDGDLTLFESRAITQYLAEEYSEKGEKLISQDCKKVKATTNVWLQVEGQQFDPNASKLAFERVFKGMFGMTTDPAAVQELEGKLQKVL
DVYEARLAKSEFLAGDSFTLADLHHLPAIHYLLGTDSKVLFDSRPKVSEWIKKISARPAWAKVIDLQKQ
>AtGSTF11
MVVKVYGQIKAANPQRVLLCFLEKDIEFEVIHVDLDKLEQKKPQHLLRQPFGQVPAIEDGYLKLFESRAIARYYATKYADQGTDLLGKTLEGRAIVD
QWVEVENNYFYAVALPLVMNVVFKPKSGKPCDVALVEELKVKFDKVLDVYENRLATNRYLGGDEFTLADLSHMPGMRYIMNETSLSGLVTSRENLNR
WWNEISARPAWKKLMELAAY
>AtGSTU8
MNQEEHVKLLGLWGSPFSKRVEMVLKLKGIPYEYIEEDVYGNRSPMLLKYNPIHKKVPVLIHNGRSIAESLVIVEYIEDTWKTTHTILPQDPYERAM
ARFWAKYVDEKVMLAVKKACWGPESEREKEVKEAYEGLKCLEKELGDKLFFGGETIGFVDIAADFIGYWLGIFQEASGVTIMTAEEFPKLQRWSEDF
VGNNFIKEVLPPKEKLVAVLKAMFGSVTSN
>AtGSTU27
MSEEEVVVLNFWPSMFGARVIMALEEKEIKFEYKEEDVFGQKTDLLLQSNPVNKKIPVLIHNGKPVCESNIIVEYIDEVWKDDKTLRLLPSDPYQKS
QCRFWADLIDKKVFDAGRRTWTKRGKEQEEAKQEFIEILKVLERELGDKVYFGGNDNVSMVDLVLISYYPWFHTWETIGGFSVEDHTPKLMDWIRKC
LTRPAISKSLPDPLKIFDRVTQIIKVHEFFYGY
>AtGSTL2
MSVGLKVSAFLHPTLALSSRDVSLSSSSSSLYLDRKILRPGSGRRWCKSRRTEPILAVVESSRVPELDSSSEPVQVFDGSTRLYISYTCPFAQRAWI
ARNYKGLQNKIELVPIDLKNRPAWYKEKVYSANKVPALEHNNRVLGESLDLIKYIDTNFEGPSLTPDGLEKQVVADELLSYTDSFSKAVRSTLNGTD
TNAADVAFDYIEQALSKFNEGPFFLGQFSLVDVAYAPFIERFRLILSDVMNVDITSGRPNLALWIQEMNKIEAYTETRQDPQELVERYKRRVQAEAR
L
>AtGSTF13
MAMKLYGDEMSACVARVLLCLHEKNTEFELVPVNLFACHHKLPSFLSMNPFGKVPALQDDDLTLFESRAITAYIAEKHRDKGTDLTRHEDPKEAAIV
KLWSEVEAHHFNPAISAVIHQLIVVPLQGESPNAAIVEENLENLGKILDVYEERLGKTKYLAGDTYTLADLHHVPYTYYFMKTIHAGLINDRPNVKA
WWEDLCSRPAFLKVSPGLTVAPTTN
>AtGSTF2
MAGIKVFGHPASIATRRVLIALHEKNLDFELVHVELKDGEHKKEPFLSRNPFGQVPAFEDGDLKLFESRAITQYIAHRYENQGTNLLQTDSKNISQY
AIMAIGMQVEDHQFDPVASKLAFEQIFKSIYGLTTDEAVVAEEEAKLAKVLDVYEARLKEFKYLAGETFTLTDLHHIPAIQYLLGTPTKKLFTERPR
VNEWVAEITKRPASEKVQ
>AtGSTL1
MALSPPKIFVEDRQVPLDATSDPPALFDGTTRLYISYTCPFAQRVWITRNLKGLQDEIKLVPIDLPNRPAWLKEKVNPANKVPALEHNGKITGESLD
LIKYVDSNFDGPSLYPEDSAKREFGEELLKYVDETFVKTVFGSFKGDPVKETASAFDHVENALKKFDDGPFFLGELSLVDIAYIPFIERFQVFLDEV
FKYEIIIGRPNLAAWIEQMNKMVAYTQTKTDSEYVVNYFKRFM
>AtGSTL3
MAPSFIFVEDRPAPLDATSDPPSLFDGTTRLYTSYVCPFAQRVWITRNFKGLQEKIKLVPLDLGNRPAWYKEKVYPENKVPALEHNGKIIGESLDLI
KYLDNTFEGPSLYPEDHAKREFGDELLKYTDTFVKTMYVSLKGDPSKETAPVLDYLENALYKFDDGPFFLGQLSLVDIAYIPFIERFQTVLNELFKC
DITAERPKLSAWIEEINKSDGYAQTKMDPKEIVEVFKKKFM
>AtDHAR3
MISLRFQPSTTAGVLSASVSRAGFIKRCGSTKPGRVGRFVTMATAASPLEICVKASITTPNKLGDCPFCQKVLLTMEEKNVPYDMKMVDLSNKPEWF
LKISPEGKVPVVKFDEKWVPDSDVITQALEEKYPEPPLATPPEKASVGSKIFSTFVGFLKSKDSGDGTEQVLLDELTTFNDYIKDNGPFINGEKISA
ADLSLAPKLYHMKIALGHYKNWSVPDSLPFVKSYMENVFSRESFTNTRAETEDVIAGWRPKVMG
>AtGSTF12
MVVKLYGQVTAACPQRVLLCFLEKGIEFEIIHIDLDTFEQKKPEHLLRQPFGQVPAIEDGDFKLFESRAIARYYATKFADQGTNLLGKSLEHRAIVD
QWADVETYYFNVLAQPLVINLIIKPRLGEKCDVVLVEDLKVKLGVVLDIYNNRLSSNRFLAGEEFTMADLTHMPAMGYLMSITDINQMVKARGSFNR
WWEEISDRPSWKKLMVLAGH
>AtGSTT1
MMKLKVYADRMSQPSRAVIIFCKVNGIQFDEVLISLAKRQQLSPEFKDINPLGKVPAIVDGRLKLFESHAILIYLSSAFPSVADHWYPNDLSKRAKI
HSVLDWHHTNLRRGAAGYVLNSVLGPALGLPLNPKAAAEAEQLLTKSLSTLETFWLKGNAKFLLGSNQPSIADLSLVCELMQLQVLDDKDRLRLLST
HKKVEQWIENTKKATMPHFDETHEILFKVKEGFQKRREMGTLSKPGLQSKI
>AtGSTT2
MKLKVYADRMSQPSRAVLIFCKVNEIQFDEILISLGKRQQLSPEFKEINPMGKVPAIVDGRLKLFESHAILIYLSSAYASVVDHWYPNDLSKRAKIH
SVLDWHHTNLRPGASGYVLNSVLAPALGLPLNPKAAAEAENILTNSLSTLETFWLKGSAKFLLGGKQPSIADLSLVCELMQLQVLDDKDRLRLLSPH
KKVEQWIESTRKATMPHSDEVHEVLFRAKDRFQKQREMATASKPGPQSKIIQFSSIGGTSDGPNLVQDTTDRKARRRKWSPPDDVILISAWLNTSKD
RKVVVYDEQQAHTFWKRIGAHVSNSASLANLPKREWNHCRQRWRKINDYVCKFVGCYDQALNQRASGQSEDDVFQVAYQLYYNNYMSNFKLEHAWRE
LRHNKKWCSTYTSENSKGGGSSKRTKLNGGGVYSSSCNPESVPIALDGEEQVMDRPLGVKSSKQKEKKVATKTMLEEREADSRSRLENLWVLDEEEQ
VMDLPLGVKSSKQKERKVATKTMIEEREAANFRSRLGNLWLLKEKEEREADSRSRLENLWALKEKDIEEQKKLTRMEVLKSLLGRRTGETSEKEETL
KNKLIDEML
>AtGSTU9
MDEEVENKVILHGSFASPYSKRIELALRLKSIPYQFVQEDLQNKSQTLLRYNPVHKKIPVLVHNGKPISESLFIIEYIDETWSNGPHILPEDPYRRS
KVRFWANYIQLHLYDLVIKVVKSEGEEQKKALTEVKEKLSVIEKEGLKEIFSDTDGEPTVTNETMSLVDIVMCTLLSPYKAHEEVLGLKIIDPEIVP
GVYGWI
>AtGHR1
MSYSTIISNTSFLSLASKFTTRGSRLQCTVSMARSAVDETSDSGAFQRTASTFRNFVSKDSNSQFPAESGRYHLYISYACPWASRCLSYLKIKGLDD
AISFSSVKPIWGRTKETDEHMGWVFPGSDTEVPGADPDHLNGAKSVRELYEIASPNYTGKYTVPVLWDKKLKTVVNNESAEIIRMFNTEFNHIAGNP
DLDLYPSHLQAKIDETNEWIYNGINNGVYRCGFAKKQGPYEEAVEQVYEALDRCEEILGKHRYICGNTLTETDIRLFVTLIRFDEVSSYFQSKKKKY
TICERISNVETLIQVYAVHFKCNKKLIREYPNLFNYTKDIFQIPGMSSTVNMNHIKQHYYGSHPSINPFGIIPHGPNIDYTSPHDRHRFSK
>AtGHR2

MANCFAPQLTFPSFSPRHFSPRMSHQSPKPSTSTTTSIFTSATKLLWGPSLPPGLLISTARTAWTTVWQLMMTQLAPSDSSGSYTRPTSKFRLDPTQ
FTSAASSELHLYVGLPCPWAHRTLIVRALKGLNDAVPVSIASPGQDGSWEFKNNNIPIKDKDKLIPSLDKANRCRNLKEVYKSRSGGYDGRCTVPML
WDLRKKDVVCNESYDIIEFFNSGLNKLARNDNLDLSPPELKEMIQGWNQIVYPKVNNGVYRCGFAQSQEAYDGAVNELFSTLDEIEDHLGSNRYLCG
ERLTLADVCLFTTLIRFDSVYNILFKCTKKKLVEYPNLYGYLREIYQIPGVAATCDISAIMDGYYKTLFPLNASGIQPAISSSGDQDSLWRPHNRDL
VGKAIEAQLSV
>AtGHR3
MATPMENENPNFARTATSFRNFVSKDPDSQFPAESGRYHLYISYACPWASRCLAILKLKGLDKAISFSSVQPLWRNTKENDEHMGWVFPDSDTEVLG
AERDHINGAKSVRELYDIASSNYTGKYTVPVLWDKKLKTIVNNESSEILRMFNTEFNHVAENPSLDLYPPNLRAIIDETNEWIHDGINNGVYKCGFA
TNQETYDVEVKRLYEALDRCEDILRKQRFLCGNTLTESDIRLFVTVIRFDEAYAVIFKCDKRLVREYYHLFNYTKDIYQIAGMSSTVKMDHIKQNYY
GSFPSINPLEIIAHGPNIDYSLPHDRHRFSLESDYTRLELFESASFVCELKLIEIFDSL
>AtGHR4
MARSGVDETSESGAFVRTASTFRNFVSQDPDSQFPAESGRYHLYISYACPWACRCLSYLKIKGLDEAITFSSVHAIWGRTKETDDHRGWVFPDSDTE
LPGAEPDYLNGAKSVRELYEIASPNYEGKYTVPVLWDKKLKTVVNNESSEIIRMFNTEFNGIAKTPSLDLYPSHLRDVINETNGWVFNGINNGVYKC
GFARKQEPYNEAVNQLYEAVDRCEEVLGKQRYICGNTFTEADIRLFVTLIRFDEVYAVHFKCNKRLLREYPNIFNYIKDIYQIHGMSSTVNMEHIKQ
HYYGSHPTINPFGIIPHGPNIDYSSPHDRDRFSS

>OsGSTF1
MTPVKVFGPAQSTNVARVLLCLEEVGAEYEVVNVDFTVMEHKSPEHLKRNPFGQIPAFQDGDLYLFESRAIGKYILRKYKTREADLLREGNLREAAM
VDVWTEVETHQYNSAISPIVYECIINPAMRGIPTNQKVVDESAEKLKKVLEVYEARLSQSTYLAGDFVSFADLNHFPYTFYFMGTPYASLFDSYPHV
KAWWERLMARPSVKKLAAVMAPQGA*
>OsGSTF2
MAPMKLYGSTLSWNVTRCVAVLEEAGAEYEIVPLDFSKGEHKAPDHLARNPFGQVPALQDGDLFLWESRAICKYVCRKNKPELLKDGDLKESAMVDV
WLEVESNQYTPALNPILFQCLIRPMMFGAPPDEKVVEENLEKLKKVLEVYEARLTKCKYLAGDYISVADLSHVAGTVCLGATPHASVLDAYPHVKAW
WTDLMARPSSQKVASLMKPPA*
>OsGSTF3
MAAPVTVYGPMISPAVARVAACLLEKDVPFQVEPVDMSKGEHKSPSFLKLQPFGQVPAFKDSLTTVFESRAICRYICDQYADSGNKTLMGRKEDGAV
GRAAIEKWIEAEGQSFNPPSLAMAFQLAFAPFMGRATDMAVVEQNEAKLVKVLDVYEQWLGENQYFAGDEFSLADLVHMPNTDLLVRKTNKAGLFTE
RKNLARWWDEVSARPSWKKVVELQNVPRPS*
>OsGSTF4
MAGEGRKLRVYGMALSANVVRVATVLNEKGLDFDLVPVDLRTAAHKQPHFLALNPFGQIPVLQDGDEVLYESRAINRYIATKYKAEGADLLPAEASP
AKLEVWLEVESHHFYPAISGLVFQLLIKPLLGGATDTAAVDEHAAALAQVLDVYDAHLAGSRYLAGNRFSLADANHMSYLLFLSKTPMAELVASRPH
VKAWWDDISSRPAWKKTAAAIPFPPAA*
>OsGSTF5
MYQQSAGQIQLQEGPMAPMKVYGWVVSPWMARVLVALEEAGAEYEVVPMSRSGGDHRRPEHLARNPFGEIPVLEDGDLTLYQSRAIARYIFRKYKPE
FLGLGEGGSLEESAMVDVWLDVEAHQHEAAVRPILWHCIINKFEGRDRDQGVVDESVRKLEKVLGVYEARLSGSRYLAGDRISLADLSHFSNMRYFM
ATEYAGVVDAYPHVKAWWEALLARPTVQKVMAGMPPDFGFGSGNIP*
>OsGSTF6
MPGAVKVFGSPSSAEVARVLACLFEKDVEFQLIRVDSFRGSKRMPQYLKLQPHGEALTFEDGNVTLVESRKIIRHIADKYKNQGNPDLIGMGALERS
SIEQWLQTEAQSFDVPSADVVYSLAYLPAATTQPNKGAAAADGGRCEEEKNDDGGRDRQYSSQRQGGAGAGGGRDGQMAAAHRQKVEEMKQLFEKSS
KELSKVLDIYEQRLEEAEYLAGDKFTLADLSHLPNADRLAADPRTLRMLQSRRNVSRWWADVSGRESWKQVKSLNRPPSAEAPF*
>OsGSTF7
MSPVKVFGRAISTNVSRVLVCLEEVGADYELVTVDFLAGEQNSPEHVERNPFGKIPALQDGDLVLFESRAIAKYILRKYKSSKVDLLRESDIREAAL
VDVWTEVEAHQYYPALSPIVFECIIFPIMRGVPTNQQVVHESLEKLKKVLETYEARLSGSRYLAGDFLSFADLNHFPFTFYFMATPCASLFDAYPHV
KAWWEGLMSRPSIKKISANMPTKF*
>OsGSTF8
MAPVKVFGPAMSTNVARVLVCLEEVGVEYELVNIDFKAMEHKSPEHLKRNPFGQMPAFQDGDLLLFESRAVGRYILRKYKTSEANLLREGNLTEAAM
VDIGIEVEIHQYYPVISSIVYECLFNPAMYGVPTNQKVVDNSLEKLKKVLEVYEARLSQNTYLAGNFLSFVDLSHFPFTFYFMATPYASLLDKYPHV
KAWWDGLAARPSIKKVTAAMVLPLKA*
>OsGSTF9
MAPVKVFGPAKSTAVARVLVCLEEVGAEYELVGIHIPAGEQKSPAHLARNPFGQVPAFQDGDLILFDLLKESNLSQSAIMVDVWLEVESQTFDTAMS
AITFQCLTIPTFMGGIADDKIVEENLGKLKKALEVYEARSCRFRYLAGDFISLADLSHFPMTHYLLATPHASVLDAYPHVKSWINDLMKRPAVKRVR
ELMEA*
>OsGSTF10
MAPAKVYGPAMSTNVMRILVCLEEVGAEYEVVPVDMSTGEHKRPPHISRNPFGQVPAFEDGDLTLFESRAISKYILRKHGSDLLRESNLSESAMVDV
WLEVESSHFDGAMSPIIFQCFIVPMFMGGATDIGVVNESLEKLKKALEVYEAQLSKSKYLAGDFISLADISHFPTVYYLLASAHASVLEAYPRVKAW
IDDVMQRPSVKKVTEALKMPSA*
>OsGSTF11
MEKTCQAYLEQTLCWTSSLLIDFFQLLTPARREAGVEYEVVPLSLTNGDHRRPEHLARNPFGQIPVLEDGDLTLYQSHAIARYVLGKHKPELLGLGE
GGSVEESAMVDMWLEVETHQYEAAVKPIVWHCLVHQHVGLVRDQGVVDESVEKLRAVLEVYEARLSSSSAGRYSYLAGGGSGDRVSLADLSHVPLMH
YFTATEYGGVLGEYPRVKAWWEALLARPSVKKVIAGMPTDFGFGSGNLP*
>OsGSTF12
MAMKVYGLPMSTNVARVLVCLEEAGEQYEVVPIDFSIAEHKSPEHTSRNPFGQVPALQDGDLILFESRAISKYVLRKNNSELLKEHNLSDAAKVDVW
LEAESHHFDEPMSVVIYQCLILPVYFGGQTDAKVVEENLEKLKKTFQVYEERLCKFRYLAGDFLSLADLSHFPTAYYLLATPHAAMLDEFPLVKAWI
DGMLARPSVKKVIEMMKATA*
>OsGSTF13
MSPVKVFGSAPFTNVARVLLCLEEVGADYEIVDVDFGDREHKGPDHLARNPFGQVPAFQDGDLMLFESRAICRYILRKHRATDEANLLREGDPSESA
VVDAWLDVEALRYEPSVHAVFVQRRVVPALGGEPDERVIAESVARLRETLAVYEARLEATRGYLAGGEVSLADLSHFPYTRYFMEMPYEVPVFGAYP
RVTAWWERLLTRPSVRKVAAMMSGGEG*
>OsGSTF14
MAPASVKVFGSPTSAEVARVLMCLFEKDVEFQLVRVDAYRGTQRMPQYLKLQPLGEALTFEDDNLTLSESRGILRHIAHKYARQGNPDLIGTGALER
ASIEQWLQTEAQSFDVPSAEMVYSLAFLPPNMPKQNDNGNGNGNGYGNSNGREVQVANASSKRVVAGATDGKTAASGANGNKQQQKEEEMRKVFEKS

KKDLEKLLDIYEQRLEEAAYLAGDKFTIADLSHLPNADRLASDPRSRRMFEARKNVSRWWNNISSRESWEYVKSLQRPPSAAHAGNAQQQQQQQSPS
AGNNYQHQQGQGQGQQHYRNEQVENYNN*
>OsGSTF15
MAAGLQVFGQPASTDVARVLTCLFEKNLEFELIRIDTFKKEHKLPEFIKLRDPTGQVTFKHGDKTLVDSRAICRYLSTQFPDDGNRTIYGTGSLERA
SIEQWLQAEAQSFDAPSSELVFHLAFAPQLNIPADEARIAENERKLQQMLNVYDEILAKNKYLAGDEFTLADLSHLPNSHYIVNARSPRGKKLFTSK
KHVARWYEEISNRASWKQVVKMQSEHPGAFE*
>OsGSTF16
MAAGLQVFGQPASTDVARVLTCLFEKDLEFELVCIDTFKREHKLPEFIKLRDPNGQVTFKHGDKTIVDSRAICRYVCTQFPEGNKTLYGTGSLERAS
IEQWLQAEAQNFSPPSSALVFHLAFAPHLNIPQDHAVIAENEKKLQQVLNVYDEILSKNEYLAGDEFTLADLSHLPNSHYIVSSERGRKLFTGRKNV
ARWYDQISKRETWKQVVKMQREHPGAFE*
>OsGSTF17
MVAKVYGVAASPYVATVLVCLEEAGASYELVAVDMAAGENRSRHHLARSPFGKIPAFEDGEVTLFESRAIQRYVLRNYNKPDLLREGNLEESAMVDM
WMEVEAHHYDPAIFHIIRECVIKPMIGGGARDQAIVDENVEKLRKVLEVYERRLSESEYLAGDFVSVADLNHFPYTYYLMTTEYATLVESCTNVKAV
EIMGI*
>OsGSTT1
MQPLLKVYADRRSQPSRAIIIFCRVNRIDFEEVTVDLFKREHLSPEFKKINPMGQVPAIVDGRFRLFESHAILRYLATVFPGVADHWYPADLFTRAK
LEAILDWHHSNLRRGAATFILNTVLAPSLGLPSSPQAAKEAEKVLFRSLGLIESMWLKGNAKFLLGNPQLSIADLSLVCEIMQLEVLGDSERDRILG
PHEKIRSWVQNVKKATSPHFDEVHELIFKMKERMAAKRQSEPSKDLKTASKL*
>OsGSTU1
MAEEKELVLLDFWVSPFGQRCRIAMAEKGLEFEYREEDLGNKSDLLLRSNPVHRKIPVLLHAGRPVSESLVILQYLDDAFPGTPHLLPPANSGDADA
AYARATARFWADYVDRKLYDCGSRLWRLKGEPQAAAGREMAEILRTLEAELGDREFFGGGGGGRLGFVDVALVPFTAWFYSYERCGGFSVEEVAPRL
AAWARRCGRIDSVVKHLPSPEKVYDFVGVLKKKYGVE*
>OsGSTU2
MAAEGELKLLGTWASPYVSRVKLALHLKGLSYEYVVEEDHFNNKSELLLSSNPVHKKVPVMIHNGKPICESLIIMEYLDEAFPDTAAPLLPADLHDR
AVARFWAAYIDDKLVPSWKQAFSGKTGEEKAEGMRHMLAAVDALEAAMEWSYKGKPFFGGDAVGFLDVALGGLLSWLHGTEELCGAKILDAAKTPLL
SAWARRFGEMDAAKVALPDVCKLVEFAKMKRVQLEAAMAATTVSRN*
>OsGSTU5
MADEVVLLDLWVSPFGQRCRIALAEKGVEYEYSEQSLADKSDLLLRSNPVHKKVPVLLHAGRPVCESLVILEYIDETWPPEPEKKKESPRLLPSDPY
ARARARFWADYVDKKLFDCQTRLWKLRAGDAAHEQAKRDMAEALGTLEAELGEGDYFGGEAFGYLDVVLVPFVAWFHAYERLAGFAVAEICPRLVAW
GERCKGRDSVAKTLTDPEKVYEFALYLKAKFGAK*
>OsGSTU6
MAAAGDGAGGGGEQLTVLGAWGSPFLVRVRLALNLKGLSYEYVEVDLAGKSDLLLAANPVHAKVPVLLHAGRPVCESMLIVEYLDEAFPSSSSSAAA
ATTILPAADDPYARAVARFWAAFVDGELLSGWMGIYDGGKTGEERAAALARTRAALDALEGALRERAGGRWFGGERVGLVDVALGGFVPAMLASEPT
TGVRIVDADRTPLLAAWVERFCALEEAKAAMPPLERLIAAGKKRYADLQAAAAASE*
>OsGSTU7
MMNSSEPVKLIGAFGSPFVHRVEVALRLKGVPYELILEDMGNKSELLLAHNPVHKMVPVLLHGDRSAICESLVIVEYVDEAFDGPPLLPADPLRRAM
ARFWVHFLDENCLKPLRPALFGEGEEQKKSMEETREGLTVAEAELRGKRFSGGSSIGLADIAGGGVLAHWVGVLQEVAGVSVLSDGDGEYPALRRWA
KDYVANESVMECLPDRDRLLSYFTGIKQKCVSVAKSTLPKYSLLP*
>OsGSTU8
MAGHDELKLLGHWSSAYVTRVKLALHLKGVSYEYVEEDLRNKSDLLLASNPVHKTVPVLIHNGNPIRESQIIVQYIDEVFSGAGDSILPADPYERAV
ARFWAAYIDDKLLAPWKKVFRAKTEEERAAWMKQMFVAVDVLEGGLKECSKGKGCFFGGDSVGYVDVVLGGAVSFVHANDMITGGKLFDAARTPLLA
AWLERFGELDAAKAVLQDVDRAVEHTKVRYARNAATAAND*
>OsGSTU9
MAGAGRDELKLLGMWASPYVSRAKLALQLKGVSYEYIEEDLGNKSDLFLRSNPVHKTVPVLIHNGNPICESSIIVQYIDESFPSSAASLLPADPYDR
AVARFWAAYIDDKVRARVNCNLTLVYFHRCDAAHRRVDCAASGAVENGVQGEDGGGEGRAHEADARGGGRAGGRTEGVLQGEGMLLRRRQRRLRRRR
AGWPRVVGARQRQALRRQALRRRQGAAAGGVAGALRRAGCRQGRPAGRRQGGRVRQEVPAKGFRHGCRSTSSKLGRHCDVQEKY*
>OsGSTU10
MAGDGELKLLGMWTSAFVLRVRFVLNLKSLPYEFVEENLGDKSDLLLASNPVNKTVPVLLHAGRPVNESQVILQYIDEAWPDRPPAVLPSDPYERAV
ARFWAAYVDDKVRLAWLGILFRSETEEERAAAVAQADAALETLEGALRECSGGKPFFGGDGVGLVDVVLGGYLGWFTAIKKLIGRRMIDPARTPALA
AWEDLFRATDAARGVLPDDADKMLEFRQTALALGASKKITL*
>OsGSTU11
MAAGAGGDGGGELKLLGTWASPFVQRVRLALNLKGLAYEFIEEEIGGGKSELLLASNPVHKKVPVLLHRSNPICESQVIVQYLDDAFPGGAAGGDLL
PSDPHARAVARFWAAYIDAEFFAPWNRSFYTASEEEKTAEMGRAAAALATIERAFAELSRGKGFFSGEDRPGFVDVVLGGFVGSMRAYGTAVGVEVL
DAGRTPLLVAWAERVAALDAARGVIPDVERVVELSRYARKK*
>OsGSTU12
MAGGGDELKLLGMWASPYVLRVKLALSLKGLDYEYVEEDLKNKSELLLSSNPVNKKVPVLIHNGKPVCESQIILQYLDEAFPDAGATLLPADPHERA
VARFWAAFCDDTIAKASQQASSGKTEEEKAEGEKKVVEALEKMEVGLSECSKGKPFFGGDTVGYVDIVLGSFLAWVRAGDAMKGVKRFDPATTPLLA
AWAERFVELDVAKAAMPEVDKLIELAMARMAGAAAAATN*
>OsGSTU13
MAGKDDDVKVLGVVVSPFAIRVRIALNIKGVSYEYVEEDIFNKSELLLTSNPVHKKVPVLIHGGKPISESLVIVQYVDEVWAAAPSVLPADPYDRAV
ARFWAAYVDNNMFPGMAGVLFAATEEERAAKAEETLAALAQLEKAFAECAGGKAFFGGDSIGYVDLALGSNLHWFEALRRLFGVALLDAGKTPLLAA
WAKRFVEAEAAKGVVPDAGVAVELGKKLQARAAAASTAA*
>OsGSTU14
MAGEGDDQLKLLGLWVSPYTHRVKLALSFKGLSYEYVEEDLSNKSELLLSTNPVHKKVPVLIHNGKPICESQVIVQYLDEEFPNSGVSLLPSDSYDR
AIARFWAAYINDKLMPSWLQSSMGKTEEERAEALKQTLEAVANLETAFKECSKGKPFFGGDTVGYLDVSLGAMIGWMRAGEALHGRRTFDATRSPLL
NAWMERFAALDAAKAAMPDNNKLVEFVRVRRAAAANN*
>OsGSTU15
MSSTNNSSGEPPPAVRVLGGWASPFTNRVVVALKLKGVEHEMLQETVGKKSELLLRSNPVHKKFPVLLHHSKPLPESLVIVEYIDEVWPASNGGAPA
ILPRDPHGRAVERFWARYVDDKILPGLRVLRGSVAGDKDQTAGEMSTTLQRLEEAFVKCSQGKEYFGGDSIGYLDIALGSFLGWIKAVEKIAGVELL
NETKLPILAVWADRFCAHPAVVDVVPDADKLVEFTVQYGSVLNTVNVLPK*
>OsGSTU16

MEKQENGGEAPELKLFGSWASSYTHRVQLALRLKALEFVYAEEDLGNKSEALLRLNPVHKKVPVLVHRGRPLAESVIILQYLDDAWPESRPLLPSDP
FDRALARFWCHFADDKLGPAVGAVFALTGREQEAAVQQVHDNLALLEAELREGAFKGRRFFGGDQVGLLDVVLGCGSYWLAVFEEVTGVRLVDADAF
PLFHAWLRDFEAQEEVKETIPSVDRLLEYARGLRQMLLALAAGAGAGAAAAAGSSPVTTAVDAPAPAAPPAAPQAAAVDI*
>OsGSTU17
MAADKGVKVFGMWASPMAIRVEWALRLKGVDYEYVDEDLANKSEALLRHNPVTKKVPVLVHDGKPLAESTVIVEYIDEAWKHGYPIMPSDPFDRAQA
RFWARFAEEKCNAALYPIFMTTGEEQRKLVHEAQQCLKTLETALEGKKFFGGDAFGYLDIVTGWFAYWLPVIEEACGVEVVTDEALPLMKAWFDRVL
AVDAVKAVLPPRDKLVALNKARREQILSA*
>OsGSTU18
MAAGGGGGDELKLLGLWASPYVLRAKFALSFKGLSYENVEEDLHNKSELLLSSNPVHKKVPVLIHNGKPICESQIIVEYVDEAFPDAGESLLPSDPY
DRAVARFWAAYINDKFMPAWQKASLGLTEEEKAEAVKQMLAAIENLETAFKELSKGKPFFGGDTAGYLDVTLGAVVGWARAGEVLFGRKLFDATRSP
LLAAWMERFVALDAVKAVLPDNAELIEYGKMRMAHYAKLAAALAAANKK*
>OsGSTU19
MAGGGEELKLLGMWASPFALRAKLALSFKGLSYDYVEEDFKNKSDVLLSSNPVHKKVPVLIHKGKPICESQVIVQYIDEVFPDAGVTLLPADPHDRA
VARFWAAYIDEKLFSAWILVFRSKTEEEKAEAVKQTFAVVEKLEGALSECSKGKPFFGGDTVGYVDVVLGGFVAWVHAIEEVFGLNQFDAAKTPLLA
AWLERFDELDAVKEVMPDIGRLVELAKMRQAQAAGAAAAAAGEAS*
>OsGSTU20
MAGGGDDLKMLGVYVSPFPLRVKLALSFKGLSFEYVEEDLHNKSDLLVSSNPVHKRTPVVIHNGKPISESMVIVQYLDEAFPGAGAALLPSDPLDRA
VARFWASYVDDKLFSAWKMVFKGKTEEEKVEGRKQTFAVAETLEGALRECSKGKPFFGGDAVGYVDVALGGFVPWVHAMEELFGLKQFDAAKTPLLA
AWLERVGELEAYKAVMPDAGMMIEFKKKQAQEAAAEA*
>OsGSTU21
MSSSSTSGSAEPAAAVRVLGSWTSPFVMRVVVALKLKGVEEYELLQETRGKKSELLLRSNPVHKKIPVLLHHGKPLAESLIIVEYIDEVWPASDGAP
AILPRDPYCRAVERFWAQYIDDKFPRGTRVLRGTVAGEEPWLKCPPL
>OsGSTU22
MAGGGDELKLLGMWASPFALRAKLALSFKGLSYDYVEEDFKNKSELLLSSNPVHKKVPVLIHNGKPICESQVIVQYIDEVFPDAGVTLLPADPHDRA
VARFWASYIDEKLFGAWIPVFRGKTEEEKAEGVKQTFAVAEKLEGALSECCKGKPFFGGDTVGYVDVVLGGFVAWVHAIEEVFGLNQFDAAKTPLLA
AWLERFDELDAAKEAMPDIGRLVELEKMRQAQAQAAVY*
>OsGSTU23
MAGKDDDVKVLGLVMSPFAIRVCIALKLKGVSYEYIEEDLANKSELLLSSNPVHKKIPVLIHGGKPVSESLVIVQYVDEAWAPSPTSPSILPADPYD
RAVARFWAAYVDDKMVPGMVGVLRAATEEERAAKADETLAAMAQLEKAFAEVAAKNGKPFFGGDTVGYVDLALGCNLHFLEAIRRLHGVALVDAGKT
PLLAAWAERFVEVEAAKGVVPDADDAVEFARKVQARVAAAAASTAAK*
>OsGSTU24
MAGGGDELKLLGMWASPYVLRVKFALSLKGLSYEYVEEDLMNKSDLLLSSNPVNKKVPVLIHNGKPVCESQVILQYLDEAFPGAGATLLPADPHERA
VARFWAAFNDDTLVEASQAASWGKTEEERAEGEKKVVEALEKMEVGLRECSKGKPFFGGDTVGYLDVVLGGFLAWVRATDVMRGVKRFDPATTPLLA
AWAERFVELDAAKAVMPDMDKMIEFGKVLQARAAAATN*
>OsGSTU25
MAAEDELKLLGFWASPYVCRVKLALHLKGLIYDYVKEDVFTNKSELLLSCNPVHAKVPVLIHNGKPICESQVIVQYIDEVFPDAGVTLLPADPHDRA
AARFWAAYIDDKLLPPWVHAYRGKTDEEKAERMKQTLAVVDALETAMEECSKGNAFFGGDTVGYVDVALGGLLSWLHGTEELCGAKILDAAKTPLLS
AWARRFGELDAANAALPDVGRLVEFCKMRHVELEAAEAAAARN*
>OsGSTU26
MAGGGDELKLLGTWFSPFVSRVKFVFHLKGLSYENIEEDLKNKSELLLKSNPAIKKVPVLFHNGKPLCESMIIVEYIDETFAGVGPSVVPTDAYERA
VARFWVSYIDNKLVAPWFQVFRSKSMEEKAEGLKQIFVAVMVLEEAFKECSKGRPFFGGDNAGIVDIALGSQLGWVRASQALSGIKLFDPAKTPLLA
AWAERFLALDAAKASMPEFGRLIEYAKMRQAESDAANAAAN*
>OsGSTU27
MAGGGDELKLLATWFSPFASRVKFVFHLKGLSYENIEEDLKNKSELLLKSNPVIKKVPVLLHNGKPLCESMVIVEYLDETFAAVGPSVVPADPYERA
VARFWVSYIDNKLVAPWFQVFRGKTKEEKAEGLKQMFEATAVMEVAFRECSKGRPFFGGDAVGIVDVALGSQLGWLRASETLSGIKLFDPAKTPLLL
AWAERFLALDAAKASMPESGRLLAYAKMRQAETDAANASK*
>OsGSTU28
MAGRNNHELKLLGTWPSPFVVRVRLALGLKGLSYEYVEQDIRDKSELLVVSNPVHKKVPVLIHGGKPVCESQIIVQYIDEAFPGAGASLLPSDPHER
AVARFWATYIDDEFATKFRAMGEAKEEEEKDEAAAQVFAALETLEEAMKGKVFFGGDSAGYVDVALGGFLGWIKAAEALAGVAFLDGARTPLLAAWA
ARFSALEAAKEAIPSVERLREFHGAMHAAAATVAGN*
>OsGSTU29
MAGAGDELKLLGMWTSPFALRVKLALSFKGLSYEYAEEDLSNKSELLLSSNPVHKKVPVLIHNGKPICESQVIVQYIDEAFPGAGVPLLPSDPYERA
VARFWAAYIDDKLLKSWLQASMCKTEQEKAAAMKETFAAVANLEAAFKECSKGKPFFGGDAVGYVDVTLGAVIGFVRVGEAVHGMRLFDASRSPLLD
AWLDRFAALDAAKAVLPDTGRLAEYAKMKQAEWAAAATN*
>OsGSTU30
MAGGGGAGELKLLGHWASAYVTRVKLALHLKGVSYEYVEEDLRNKSDLLLASNPVHKTVPVLIHNGNPIRESQIIVQYIDEAFSGAGDSLLPADPHE
RAVARFWTAYIEDKLVAPWEKVFRAKTEEERAAWMKQMFVAVEALEGGLKECSKGKGCFFGGDSVGYVDVVLGGGVSFVHANDVITGGKLFDAAKTP
LLAEWLGRFGELDAAKAVLQDVDRAVEYTKVLYARNAATTAANN*
>OsGSTU31
MSSTNSSGDPAAVRVVGGWASPFMNRVVVALKLKGVEHEMLQETVGKKSELLLRSNPVHKKIPYIDEVWPASNGAPSILPRDPYGRAVERFWAKYID
DKIPPGIRVLRGSVEEDKDKAAGEMSTALQHLEEAFVKCSQGKQYFGGDNIGYLDIALGSFLGWIRAVEKIAGVELLNETKVPNLAAWADRFCAHPA
VVDVVPDADRLVEFTVQHAALLRAVNVPK*
>OsGSTU32
MAHHHFFAPRKQRRSAAQERGKRELDGMDQEVWADMGTAVRVGAPPLLHLCTQLLPHPTAAGKLLLSSSNGMQPPHCTKKKQIAKMSSTNSSGDPAA
VRVVGGWASPFMNRVVVALKLKGVEHEMLQETVGKKSELLLRSNPVHKKIPVLLHHGKPIAESLIIV*
>OsGSTU33
MAAAAAGGGGSPDHELKLLGSTNPSPFVTRVELALALRGLTYDLVAVDLDRKTDLLLAANPVHAKVPVLIHRGRPVCESRVILEYIDDAFPFPGGGG
APLLPPADDPLARAAARFWAAHVDDEFVASWRPAYLGSTEGERAEGMARMAAAVGALEGALAAAEGKPFFGGDAPGLVDVTLGSVIPRTRANEALTG
TRVLDAARTPLLAAWAERFGELDAARKVLPAVGDVVEYLETRLRRSNVVIARKQ*
>OsGSTU34

MAAAAEEGEGVRLLGGRMSPFTMRARMALALRGVEYELVEEALHPRKSGRLLAANPAYGRIPVLLLPGGRAVCESAVIAQYVDDAWGGAGAGAAILP
VDPYERAMHRFWTAYIDDKFWPALDAISLAPTPEARATATASTRAALKLLEEAFAARSNGGAFFSGGGAAASPGLLDVALGCFLPALWACENLNGLR
LLDDDATPLLRAWSARLAATPAAMAVMPETEEVVAFTRFLQTKFGVAGSK*
>OsGSTU35
MGERVKLIGAFASAYGHRAEVALRLKGVRYELILEDLRNKSDLLLNHNPVHKLVPVLLHGDRSLSESLVILEYIDESFHGPPILPTDPYDRAVARFW
AQFIDQKFGRFNFWIPFVQMEGNMQDCFVREAKENLALLEGQLKGRRFFGGDAIGFLDIAACLIAHWLGAFEEVCGVTLATDEEFPALCEWRRRYVN
DEAVKPCLPNRDELVAYYRERKEMIKAAGRQHK*
>OsGSTU36
MADPVKLIGAFGSPFVHRAEVALRLKGVAYEFIHEDLDNKSDLLLAKNPIHKKVPVLLHGDRAICESLVIVEYADEVFDGRPILPTDPYDRAMARFW
AHFIEHKCSRSSWLALWLDGEEQEGLLKETKENLALLEAQLHGKRFFAGDSVGYLDIVASGLAHWISVVEEVTGVSLMGGADEDDEYPALRRWAKEY
TTDETVMQCLPSREHLAAFFAAKKDKLKMVAKAMLHQ*
>OsGSTU37
MADPVKLIGAFGSPFVHRVEAALQLKGVAYELIHEDLENKSNLLLASNPVHKKVPVLLDGGRAICESLVIVEYVDDAFDGPPILPADPYDRATARFW
AQFIDHKCTLPLLLALWLDGEEQKGFLKETKENLSLLEAQLEGKRFFAGDAVGYLDVAAGGMAHWIGVLEEVTGVSVIGSEDDDEYPSLQRWIKEYA
NIDAVKLSLPDREELVAFYTRNKDKYKMMFRAMVHQ*
>OsGSTU38
MAMTTAAVAAAAQPKEVKLYGAWGSAHAAMARNALELKGVRYEYVEEDLERKSETLLLRLNPAHAGKVPVLVVVDDDGGGGGCPLAESLVILEYVDE
VWPQAPRLLPPPSSPRARAAARFWARFFHGEVSPLSRAAAVLAPTPEERAEAVREMKARMAVMEAGFERDFPSSVVGGPFVHGATPGLLDVILGSCA
AGTRAISAMAGEEVVEPDALPHVHASMAAFDERVAGFGTSVPHELLLARLLEREERRRAAASASA*
>OsGSTU39
MAGRGGGGELRLLGTWSSPWVIRVRVALGMKGLSYEYTEEDLSSKSDHLLRSNPVHEKVPVLIHGGRPVCESLVVLEYIDETWGATGTPQLLPADPY
DRATARFWTNYVNDTFFPSWKVLFRSTAAEQRAEAFKNVVPRVEALERAFGECSKGKAFFGGDDAGLVDVALGSHLVWIKVVDEVAGANLLDEAKFP
GLAAWAERFLAVDAVRQVMPDAGDVLKQYKGFLAKWTAGAGSS*
>OsGSTU40
MAGRGGGGGGGELQLLGTWYSPYAMRAKIALGLKGLSYEYIEQDLFGKSELLLKSNPVHKKVPVLIHAGRPVCESRVVLEYVDEAWPGAAPPLLPAD
PHDRATARFWATYFDSTFFPPWRALMRATTAEQRAEAFMNAVPQVEVLERAFVECSKGKAFFGGDAVGLVDVVVGGFVVWFKVVDEVAGSSLLDEAK
FPGLAAWAERFLAVDAVREAMPDAGKLLEHYKGFLAKLASPAGST*
>OsGSTU41
MVKLISAFGSPFGHRAEAALRLKGVQYELLLEDLRSKSDLLLAHNPVHKLVPVLLHSDGRSVAESLVVVQYVDDAFHGPPLLPADPYARAQARFWAQ
FIDDKFSRPFWLSFWMEDGEKKEAFVREAKENLRPLEAQLDGGNKRFFGGDAIGLVDIAASGLAHWVGVFEEVTGVSLVSEREFPALCRWSQRYVND
GAVRQCLPSRDELVALFTANKEAYTLLAKAKLQK*
>OsGSTU42
MASPAPAPVKVIGTFDSPYSQRAEAALRLKGVPYELILEDLRNKSDLLLTHNPIHKKVPVLLHGDDDHRAAVCESLVIVEYVDEAFPAPLLLLPADP
GLRAAARFWARFIDDKCTKPFWLALWSTDDGEVREGFAAEIKENLKLLEAQLKGRRFFGGDAIGYLDLAASGYAYWLEVLEEVAGVSLVTGDEFPDL
CRWAKEYAADDRIKACLPDRAKLLEHFTAMKEMFMATARSMAAK*
>OsGSTU43
MANLVKLIGAFGSPFVHRAEVALRLKGVAYEFIHEDLNNKSDLLLAKNPIHKKVPVLLHGDRAVCESLVIVEYIDEAFNGPPLLPADPYHRAMARFW
AHFIDHKSTRPSWLALWLEGEEQKGFLKETKENLALLEAQLGGKRFFAGDSIGYLDIAAGGLAHWVGVLEEVTGVSLVAGDDGDDEYPALRRWTNEY
TANDAVKLCLPNRERIAAFFTPKDKYKIMARAMLRQQ*
>OsGSTU44
MEGEKKSVVLINCAVSMYGNRVRIALARKGVAYEEKPENLAAKSALLLSSNPVHGQVPVLLVGGKPVCESLVILEFIDEEFAGVGEPLLPAGPYERA
QARFWASYIDAKLAPCAGRVWRSPAGAAGAAAVEAARGELVAAMRTLEAELGGRRYFGGGGEALGYVDVALAPFTAWFATYERFGGFSVAAECPELA
AWAARCVRENACVAASLPDPEFVYQFACGMRKHFGLDG*
>OsGSTU45
MRARIALHVLQVGFGFVEEDLRIRERSDLVLRMNPVHRSVPILIHRGRPICGSINILQYIDEVWAKRVGTRLLPPDPLKRASARFWADFVDHEVFST
QTRFLKSKGEEKEMAKAELLDQLRRLEGVLGDRSFFSGDEFGFLDIVLIPFSSMFHGYKQHMWVKRCKERESVRQVLPDEGEMYELHKKWYGIE*
>OsGSTU46
MAGGELVLLDFWASPFGQRCRIALAEKKLPYDYSEQELLGAKSDLLLRSNPIHAKVPVLLHGDGDGRAVCESLAILEYLDDAFPDATPRLLPSAADD
PYARARARFWADYVDKKVYPVGTRLWKVKGEEGVRAAAGARGELVEALRTLDGELGEKEFFGGEFGFVDVALVPMMPWVYSFARYGGFSVEEECPRV
AAWARRCMERDSVAGSLRSPEEIYDFIGLLRKHYGIDD*
>OsGSTU47
MRVMVALRLKGVEYELLQETMGKKSELLLASNPVHKKIPVLLHRGKPISESLVIVQYVDEVWPPPASILPRDDPYAAAIHRFWGQYIDDMFPPRIRI
LRGTVPGDKDEASDEMTTALLYLEEAFVECSKGKQYFGDDSIGYLDIALGSHLGWIRAVERIAGVELLGGAKVPNLAAWADRFCGHPAVVDVMPDVD
ILVEFTAKLI*
>OsGSTU48
MAEELKLLGSLSGVSPYVIRAQMALAVKGLAHDYLPEDLTRKSKLLLDSNPVHKSVPVLIHNGKPVCDSLVIVEYVDEAFPGGAAALLPADPYHRAV
ARFWAAFIDSKVFPPCLAILKTAAAEAEEEKAAKVKETVEALQLVEGAFGECSKRKPFFGGDAVGYLDVVLGCYLCWFEGVSEIAGGVSPPLLDASR
TPQLAAWAARFRSAADAVGCSVPRVDKVEAYLNNVLKPKWSAAAAASSH*
>OsGSTU49
MVVGAGGGDELKLLGVWDSPYVNRVQIVLNLKGLSYEYVEEDLMNKSDLLLGSNPVHKKVPVLIHNGKPIAESRVIVEYLDEAFAAGAGGSTGASVL
PSDPYERAVARFWAAYVDDKVGSPWYTILFARERGEKVEAAARAISALETVEAGAFRDRSSEGKTTNAAAAPFFGGDSIGFVDVVLGSYLGWFRVIE
KMIGVRIMDAARTPRLAAWAERFEAADAVRGVLPDDVDKVIDFLQAFLH*
>OsGSTU50
MAGSGELKLLGVWSSPYAIRVRVVLNLKSLPYEYVEENLGDKSDLLLASNPVHKSVPVLLHAGRPVNESQVIVQYIDEVWPGGAGGRPSVMPSDPYE
RAVARFWAAYVDDKVRPAWLAILFGSKTEEERAAAVAQAVAALETLEGAFGECSKGKPFFGGDGVGFVDVVLGGYLGWFTAIDKLIGRRLIDPARTP
ALAAWEERFRATDAAKGVVPDDADKLLEFRQTLLRWSASKAK*
>OsGSTU51
MDHQELEEGAEKMKLLGIWSSPYVVKVIWALRIKHVEYDIEEDLRNKGNLLLECNPVHQKVPVLIYQGKPSDVIIEFIDDVWKDSGQGRIYSTQLSP
PIWKWFTTQGKEQEDA*
>OsGSTU52

MWALRIKGVEYDYIEEDLRNKSNLLLECNPVHKKVPVLIYQGKPIAESDVILEFIDDVWKDLRYRILPEDPYECAMARFWSKFGLDKLSPPIWKWFT
TQGKEQEDAYEAAMEQLLVLEKVLDEKKFFGGERIGFVDLSLGSLSYVIPIYEDITGVRLITSDKFPWLSAWMEGFLGLPLVKEHLLPLDKLRPRYQ
AIREAFLSK*
>OsGSTZ1
MAAAEKTKPVLYSEWMSSCSYRVRIALNLKGIDYEYRAVTRGDPDYGKINPIKYVPALVDGDFTISDSLAIILYLEDKYPQHPLLPQDLKKKALNMQ
IANIVCSSIQPLQCYAVIGLADGKMSANESLQIVQHYTDKGFRAIEKLLEGCRSKYATGDEVQLADVFLAPQIHAGITRFQIDMSKYPILARFYKAY
MELPAFQAAVPENQPDAPSS*
>OsGSTZ2
MASSKPILYGAWISSCSHRIRIVLNLKGVDYEYKSVNPRTDPDYEKINPIKYIPALVDGDLVVSDSLAIALYLEDKYPQHALLPKDLKKKALNLQIA
NIVCSSIQPLQGYAVIGLHEGKLSPDESLQIVQHYIDKGFKAIEKLLEGSNFKYATGDEVQLGDVFLAPQIHAGINRFQIDITKYPNLARLHDTYME
IPAFQAALPKNQPDAPSC*
>OsGSTZ3
MAEAAGAAVAPAKLGLYSYWRSSCSHRVRIALNLKGLEYEYKAVNLLKGEHSDPEFMKVNPMKFVPALVDGDAVIGDSYAIALYLEDKYPEHPLLPQ
DLKMKALNLQIASIVCSGIQPLHNLTVLVRTDLHSISYCHRFIEKKVGTGESIPWTQQQIDRGFAAAENLVKGCAGKYATGDEVRLADVFLAPQIYA
AVTRFQINMLNYPTLARLHEEYMKHPAFQAALPDRQPDAPSST*
>OsGSTZ4
MASSGSPEARQTHGEIAGAAAPERRLKLYSFWRSSCSYRVRIALSLKGLDYEYKPINLLANEQSHPEFEKLNPMKYVPALVDGDDTVVVDSFAILLY
LEDTYPQHPLLPQDPKMKALNIQIASIVGSSIQPLQNNSVLDFIEEKLDSQEKVNWIQYHLNRGFTALEKMLKGCTTTYATGDEIQLGDLFLEPQIY
GGIKRFGIDMTNYPTLARLHEAYMEHPAFQAALPERQPDAPSSPEI*
>OsDHAR1
MGRHVRMTITLPSPSRIPKPPEHGGTASWAPHVILTPSVSGELPLPINPASPISPVISPPIARRRRNPKSSLPVREKRQVVVAAMGVEVCVKAAVGH
PDTLGDCPFSQRVLLTLEEKKVPYEMKLIDVQNKPDWFLKISPEGKVPVFNGGDGKWIPDSDVITQVIEEKYPTPSLVTPPEYASVGSKIFSCFTTF
LKSKDPNDGSEKALLTELQALEEHLKAHGPFINGQNISAADLSLAPKLYHLQVALEHFKGWKIPEDLTNVHAYTEALFSRESFIKTKAAKEHLIAGW
APKVNA*
>OsDHAR2
MAVLLRTTTSATTATSGGSSSATALLATTFRRGGRRLLLLPATRGSAPRRAALLTARASAEPLEVCAKASLTVPDRLGDCPFTQRVLLTIEEKHLPY
DIKLVDLANKPDWFLKISPEGKVPIVKLEEQWVADSDVITQAIEEKYPEPSLATPPEKASVGSKIFSTFIGFLKSKDPNDGTEQALLSELTSFDSYL
KDNGPFINGETISAADLSLAPKLYHMEIALGHYKNWSVPDSLSHVKKYMKTIFSMDSFVKTIALQEDVIAGWRPKVMG*
>OsTCHQD1
MQLYHHPYSLDSQKVRMALEEKGIDYTSYHVNPLTGKNMNVAFFRMNPSAKLPVFQNGAHVIYRAFDIIQYLDRLSVHLSGEIVPVNTEVYQWMQKV
DSWNPKMFTLTHTPIKYRTFVSKFIRRVLIARMAEAPDLASMYHAKLREAYETEDKLKDPDIMKQSEEELSKLLDDVEAQLNNGKYLAGDEFSPADS
VFIPILARITLLDLDEEYINCRPRLLEYYTLVKQRPSYKVAIGKFFGGWKKYRTLFKTSFFLCVRTLFRKY*
>OsEF1G1
MALVLHTFDGNKNAFKALIAAEYSGVKVELAKNFQMGVSNKTPEYLKMNPIGKVPILETPDGPVFESNAIARYVTRSKSDNPLYGSSLIEYAHIEQW
IDFSATEVDANTGKWLFPRLGFAPYVAVSEEAAIAALKRSLGALNTHLASNTYLVGHSVTLADIVMTCNLYMGFARIMTKNFTSEFPHVERYFWTMV
NQPNFKKVMGDVKQADSVPQVQKKAAAPKEQKPKEAKKEAPKEAPKPKAAEKPEEEEEAPKPKPKNPLDLLPPSKMILDEWKRLYSNTKTNFREVAI
KGFWDMYDPEGYSLWFCDYKYNDENTVSFVTMNKVGGFLQRMDLCRKYAFGKMLVIGSEPPFKVKGLWLFRGPEIPKFVMDEVYDMELYEWTKVDIS
DEAQKERVSAMIEDLEPFEGEALLDAKCFK*
>OsEF1G2
MALVLHCGSGNKNAFKALIAAEYTGVKVELTKNFEMGVSNKTPEFLKMNPLGKIPVLETPEGAVFESNAIARYVARLKDNSSLCGSSLIDYSHIEQW
MDFSATEVDANIGRWLYPRLGFGPYVPVLEEFAITSLKRSLGALNTHLASNTYLVGHSVTLADIVMTCNLYYGFVRILIKSFTSEFPHVERYFWTMV
NQPNFKKVIGDFKQAESVPPVQKKAAPPKESKAKEAKKEAPKEAPKPKVEASEEEEAPKPKPKNPLDLLPPSKMILDEWKRLYSNTKTNFREIAIKG
FWDMYDPEGYSLWFCDYKYNDENTVSFVTMNKVGGFLQRMDLCRKYAFGKMLVIGSTPPFKVKGLWLFRGQDIPKFVMDEVYDMELYEWTKVDLSDE
AQKERVNAMIEDQEPFEGEDLLDAKCFK*
>OsGSTL1
MAAAAAPRSSGKEALPAALGSASEPPRLFDGTTRLYICYFCPFAQRAWIIRNFKGLQDKIELVGIDLQDKPAWYKEKVYEQGTVPSLEHNGKIMGES
LDLIKYIDSHFEGPALLPEDPEKRQFADELIAYANAFTKALYSPLISKADLSAETVAALDKIEAALSKFGDGPFFLGQFSLVDIAYVTIIERIQIYY
SHIRKYEITNGRPNLEKFIEEINRIEAYTQTKNDPLYLLDLAKTHLKVA
>OSGSTL2
MAAAAAAPASSEKEVLPPSLTSSSEPPPLFDGTTRLYVAYHCPYAQRAWIARNYKGLQDKIKIVAIDLADRPAWYKEKVYPENKVPSLEHNNQVKGE
SLDLVKYIDTNFEGPALLPDDSEKQQFAEELLAYTDAFNKASYSSIVAKGDVCDEAVAALDKIEAALSKFNDGPFFLGQFSLVDIAYVPFIERFQIF
FSGIKNYDITKGRPNLQKFIEEVNKIHAYTETKQDPQFLLEHTKKRLGIA
>OsGSTL3
MLSVTSTITLICICQIISTLLMYKKLQWQIVHVNICPYAQRAWIARNYKGLQEKIKLVPMDTNDRPAWYKEVYPKNTLPSLEHNNKIIGESLDLIKY
IDINFAGPRLTPDDSEKQRLAEELLAYSDIFNQAVRSALISKDMTAEAAAALDNIEFSLSKFDDGPFFLGQFSLVDIAYAPFIDGFQTLFAGIKNYD
ITEGRANIQIFIKELNKIDAYMHTKQDPSEVIALTKKKLGGRIHRWGLSLSSISAEPPALHAEQQPHGFGREAAYCISADQPGRKEGANEDNISRSM
GD
>OsGHR1
MLTRLPHHSSPLVFPCRLSAAAAARTLSTATGSNSTTVKMARSALDEVTDAGAFDRSPSTFRSSISRDSSARFPAVPGRYHLYVSYACPWASRCLAY
LKLKGLDHAIGFTSVKPIFERTRETDDHLGWVFPATGDEEPGADPDPFNGAKTIRELYEIASPNYIGKPTVPVLWDKQLKTVVNNESSEIIRMLNTE
FNEIAKNPDLDLYPAHLQTSVNEINELVYDAINNGVYKCGFAKKQGPYDEAVTRLYEALDKCEEILSRQRYICGNQLTEADVRLFVTLIRFDEVYAV
HFKCNKRLLREYPNLFNYTKDIYQIPGISSTVNMEHIRKHYYGSHPSINPYGIIPAGPNIDYNAPHDRERFSA*
>OsGHR2
MPMWSQPPPPPSSLQLRRPPPPLPHRPRRLRSRLSPIAASQDPLTALSRLLWGRALPPSQLVLAVRHGWTAAWGLLMRQLAPSDPATGAFTRTPSRF
PAVVGTPSARLHLYVGLPCPWAHRALLVRALLGLERRLPLSVAVPGDDGAWSFTPDSPDALYGKRKLREVYASARRGGFEGRASVPLLWDAERREVV
CNESIEITKFLCDLAAADGSAGGLDLWPPELRQDIDRWYSFIYPSVNNGVYRCGFAQSQEAYDAAAGELFAALDRLEDHLSGSRYLCGDTLTLADVC
LFTTLVRFDLVYHSLFRCTRRKLVEYASLHAYTRDIYQMPGVAGTCDMAAIADGYFGALFPLNPGGILPLVPASCSPEALLEPHGREALSSSAAADA
GGGGNGRQLEATSASN*
>SlGSTU1
MQNLEFIEEQLKGKKFFGGESIGYVDLALGWMAYLLDVFEEVLDLKLFDADKFPLLSGWTKNFCDAPAIKQHLPPRDKLVTKFQLFHEKFQTAN*
>SlGSTU2

MADEVKLYRTWSSRFSLRIIWALHIKGIEYEAIFEDLSHKSPQLLKYNHVHKKFPVLVHNDKPICESLVILEYIDETWKETSHLLPQDPYEKAMARF
WAKFVDDKFKNLNVLNHIIYGPKQNFIIN*
>SlGSTU3
MADEVKLYRTWSSPFGLRIVWALHIKGIEYEAIFEDLSQKSPQLLQYNPVHKKIPVLVHKGKPICESLVILEYIDETWKETAPLLPQDPYEKAMARF
WAKFVEDKLLPSVWSIFTEQGYDAKKEAFVPAVQNLEIIEEQLKEKKFFGGESIGYVDLVLGWMAYLLDVFEEVLDLKLFDADKFPLLSGWMKNFCD
APAIKQHLPPRDKLVTKFQLLHEKFQTAN*
>SlGSTU4
MASEKVKLLGYWASPFALKVHWALKLKGIEYEYQEEDLSNKSPLLLQYNPVHKKIPVLVHNGKPIAESLVILEYIEETWKHNPLLPEDPYERAKARF
WAKFVDDKCVPGIFGTFAKVGVEQQKIAKEARENLKILEDELGKKHFFGDAKIGFMDVTSAWIICWAQIVEEVVDIRLIDAEEMPSLVSWFQNVLEA
APILKECTPPKDKLLEHNKGFHEMLVASAPS*
>SlGSTU5
MGEVKVHGIFAGPFNKRVELALKLKGVKYEYIEEDRSNKSDELVKYNPIYKQVPVLVHNGKPICESIIILEYIDDTWENNTIPLLPKHPYQRSMARF
LAKLIDEKLMGAMYKVCYGKGEEREKGCDETFEVLKYLDNELQNKKFFGGDSIGFVDIVASYIALWFGAIQEAIGMELLTEQKFPKLSKWIDEFLCC
RIVKENLPNREVLVPLYKAQFAAATQKASS*
>SlGSTU6
MEDVKLLGTKESIFTQRIIWALKLKGICYEFIEQDFSSRSSPLLVKLNPVYNKVPVIVHDGKSLAESLVILEYIEETWPLINPLFPLDPFQRASTRF
WARFVDGKFYEAAKKAFFSSGETKAEGVESVVEGLHLLEGQIIGKKFFGGEKIGYLDIITGWIAYWFQYIEEIGEFKAMDSTKYPCLHAWINNFIQL
PIIKQSLPTPDVVKSVFRGFKDAAALADAN*
>SlGSTU7
MSKDDELRLLDFWASPFCMRVKIALSEKGVAYESQQEDLFGGKSDMLLKSNPIYEKVPVLLDNGKPIVESNNIVYYIEDKYPSTNPLLPSCAYGRSR
ARFWADFIDKKIFEGGMCIWKSKGEELEIAKKDFIEILKKLEGAMGDKDFFGGDNFGYVDVIAIAMTSWFHAYEVFGDFKVEQECPKFGCWMK
RCLERESVSSVLPDPEKIYQCVVMLRKMHGIE*
>SlGSTU8
MGEENKVTLHGMWLSPYVKRVELALKVKGIPFEYIEEDLSNKSPLILKYNPIHKKVPILVHNGKPVNESFVIVEYIDETWKNGPQLLPEDPYERSKV
HFWAAYIQQVMESMLNIFTAEDQKQACNEFHQKFRLLEDGMKNFFPTIENRNIGLIDIWIVVAFGMCKAQEEAFGVNFLDPEKVPLIHSRVNS
LLELPLLRET VPDHDKAVSFLRALKETSTK GQAH*
>SlGSTU9
MEEENDRVTLHGMWISTYAKKVELALKIKGIAFDYVEEDLSNKSSLLLKYNPIHKKVPLLLHRGKPLSESLVILEYIDETWNNLQPLLLPEDPYERA
TVRLWASYCLQISDTMKKAFISARDVEGGAFDELFEILKVMEEGMKDFFPGGRSKICAENLGLLDIIIVCSLATYKAAEEVVGTKILDPEKNPFVYS
WVTTLLELPLVKETLPPHDKLVSRLDFIKKNGFRFQ SNI*
>SlGSTU10
MAKNNLKILGAWPSPYVMRPRIALNIKCLAYDFLEEQFGTKSELLLKSNPIYKKIPVLIHDGKPICESLIIVQYIDENWTNFGHSILPSHPYDRAIA
RFWAFYIDDKWFPALCGVAAAQDEDAKKAAMETVIEGLVLLEDVFKINSKGKKFFGGDKIGYLDIALGCFLGWLKVNEKLNNVNLLDESRTPSLYQW
AKDFCVDSVVKDVMPETDQLVQAAKIIWAKI*
>SlGSTU11
MRPRIALNVKSVCYDFLEEQLSSKSDLLLISNPVYKKIPVLIHDGKSICESLNIVQYIDEKWTNSGPSILPLDPYDRAIARFWACYIDDKWFPLFRS
FAVAQGEDAIKTALEPVFDGLVLLEDAFKNCSKGKKFFGGDKIGYVDIALGCFLGWMRVIEKMNNVTLLDEAKTPGLYNWAEDFCADSSVKDVMPET
NKLAEAAKNLIPKIRANASS*
>SlGSTU12
MATNSVKLLGTWACPYVYRVEIALKMKSIEYEFIQERVFNKSELLLKSNPVYKKIPVLFHDEKPICESLVILQYIDEAWLNGPAILPSDPYDRAIAR
FWAAYIDEKWYPLVADYRNVEGKEAKAAMVEKISEGTLLLEEAFINMSKGKSYFGGDSIGYVDIVFGSLLGWVKVIEIVDELKILDETKTPSLAEWD
EKFCSHNVVKDIIPETEKLVEIYHKYVELKKANLS*
>SlGSTU13
MATSIIKVLGTPASPFANRVSIALNVKSVDYEFVQEDMSNKSELLLKSNPVYKKIPVLILGENIICESLVIVQYIDETWTNGPSVLPSNPLDRAITR
FWVAYIDCKWLPLMSDLGKAQGEEAILEVQEKLQQALVPLEEAFVKCSKGKSFFGGENIGYIDIALWCILGWIKAIKIMLGIEIFNVTKAPELVNWG
NRFLEDKCVKGAMLEPEKLVEIVKLHLAKKEANNAN*
>SlGSTU14
MNIFKVNGWTCTPPLQLSLKIKSIEYEFIQEHILNKSELLLKSNPVNKKIPILFHDEKPICESLVILQNIDEPWLNGPSILSSDPYNRSIARFWAAY
IDDKEYRNAEGKEAKAVVVDKMSEGNMLLEETFIKISKGKSFFSGDSIGYVDIVLGSLLGWVR*
>SlGSTU15
MGQVKLIGSSGSLFCTRVEWALKLKGVDYEYIQEDLLNKSELLIKSNPVHKKIPVLLHDDKPVVESLLILEYIDETWKGYPLLPQDPHERATARFWA
KFVDDKCVIGSWEAMAMQDEGEAKTKAIESIQELYAFIEKQIEGKKFFGGEQIGYLDLVMGWKTLWLSAMEEVGNVKLLDPEKFPSLHQWAENFKQI
PIINECMPQQETLVNYFQVGLNYLRSLAANKP*
>SlGSTU16
MKEENNVTLHGMWAKSTNTVHNGNQICESSVIIEYIDETWKNESPLFPQDPYQRIKVRFWASYIHQVYDCMLKVFRGKEALEGFYAKLSVLEDGINN
FSLGITSNMNNIGMLDIMIVITLGAYRVQEEVFGFKLLEEENTSLLYSWVTTLIELPIVKGITPPHEKVVSFLQYLKNKVFKAPPHAS*
>SlGSTUU17
MTGRRVWSGKGEDQEEAKELIEIFKTLEGELGDKTYFGGDEKLGFVDVTCANFSIEAECPKLVAWAKRCMEIENVSNSLTHPHKIYGYVLELKHKVG
LA*
>SlGSTU18
MANDEVILLDFWPSMYGMRLRIALAEKEIKYEYRDEDLRNKSPLLLQMNPIHKKIPVLIHNGKPICESIIGVEYIDEVWKDKAPLLPSDPYERAQAR
FWADYIDKKLYATGSKIYTATGDEQEAGKKDFVEILKVLEGALGEKPYYGGDNFGFGDIALIGFYCWFHAYEVYGNFSIEAECPNLVAWAKRCMQRD
SVAKTLPDQHKIIEFVKILREKLGLE*
>SlGSTU19
MNPIHKKIPVLIHNGKSICESIIGVEYIDEVWKDKAPLLPSDPYERAQARFWADYIDKKLYRSARKIWGTKGEEQEAGKKDFIEVKVLEGALGEKPY
FGGDNFGFVDIALIGFYSWFHSYETYGNFSTEAECPKFVYHQF*
>SlGSTU20
MANDELILLDFWASMFGMRLRIALAEKGIKYEYKEEEGLISNKSALLLEMNPIHKKVPVLIHNGKPICESIIGVEYIEEVWKDKAPLLPSDPYERAQ
ARFWVDYIDKKLYVSARKIWGTKGEEQEAGKKDFIEVLKVLEGELGEKPYFGGDNFGFVDIALIGFYSWFYAYETYGNFSAEAECPKFVAWAKRCMQ
RDSVAKSLPDQHKVLEFIQMLRRKFGIE
>SlGSTU21

MANDEVILLDFWPSMFGMRLRIALAEKEVKYEYKEEDVWNKSPLLLEMNPIYKKVPVLIHNGKPICESIIGVEYIEEVWKDKAPLLPFDPYERAQAR
FWADYINKKCETYGNFSLEAECPKLVAWAKRCMQRDCGQVFA*
>SlGSTU22
MANDEVIVLGFWPSMFGLRLRIALAEKEVKFEYREEDLKNKSPLLLQMNPIHRKIPVLIHNGKPICESIIGVEYIEEVWKDKAPLLPSDPYERTQAR
FWADYIDKKFYWPARKLWTTKGEEQEIAKKDFIECLKVLEGVLGDKPYFGGDNFGFVDIALIGFYCWFSAYETYGNFSTEAEFPKFFAWAKRCMQRD
SVAKSSPDQHKVLEFVKVVRQRLGIE
>SlGSTU23
MANNEVILLDFWPSMFGLRLRIALAEKEVKYEYREEDLPNKSPLLLQMNPIHKKIPVLIHNGKPICESIIGVEYIDEVWKDKAPLLPSDPYERAQAR
FWADYIDKKFYWASRKLWTTKGEELDAAKEEFIVCLKVLEGALGDKPYFGGDNFGFVDIALIGFYCWFSAYETYGNFSTEAESPKFVAWAKRCMQRD
SVAKSSPDQHKVLEFVKVVRQRLGIE
>SlGSTU24
MANDEVILLDFWPSMFGMRLRIALAEKEIKYEYRDEDLRNKSPLLLQMNPIHKKIPVLIHNGKPICESIIGVEYIDEVWKDKAPFLPSDPYERAQAR
FWADYIDKKLYDSGRKLWTTKGEEQETAKKDFIECLKVLEGALGEKPYFGGDNFGCVDIALIGYYSWFYAYESYANISVEAECPKFVAWAKNCMLRD
SVAKSLPDQHKVCEFVKVLRQKFGIE
>SlGSTU25
MADEVVLLGTYVSMFAVRVKIALAEKGIQYEYKEENLVNKSPLLLQMNPIHKKIPVLIHNGKPICESLIIVEYIDEVWNDKSPLLPSDPYKRAQARF
WADYVDKKIYDGGKKIWTTKVEEQEAANKEFIECLKVLEGELGDKPYFDGESFGFVDLALIPYYSWFPAYEKFGKFSIEPECPKFVAWANRCMQKEN
VSKYLSDPDKIYDFVVMLRQRIGIA
>SlGSTU26
MGDEVVLLDTFVSVFGMRVRIALAEKGIQYEYKEEDLMNKSQLLLQMNPIHKKIPVLIHNGKPICESLIIVEYIDEVWKDKSTPLMPSDPYKRAHAR
FWADYIGKKIYDGGMKIWSSKVEEHKTANKDFIECLKVLEGELGDKPYFDGKNFGLVDMAFIPYYSWFPVYKKLSNLNIEAECPKFVAWAKRCMQKE
SVSKTLVDPDKIYEFIVLFRQKIGVA
>SlGSTU27
MGDEVVLLDLWVSPFGMRVRIALKEKGINYESKEENLSNKSSLLLKMNPIHKQIPVLIHNGKPICESLIIVQYIDEVWKDKAPLLPSDPYERAHAKF
WADYVDKKIYSTGRLVWTTKGEAQEAAKKELIHHFKLLEKELGDKTFFGGDQFGLVDIALIPFYSWFYALETCGNFSMIHECPKLVEWAKRCMERES
VSTSLPDQYKVYDFILELKKKLDLD
>SlGSTU28
MEEENKVTLHGMWTSPYVKRVERALKVKGIHYEYVEEDLMNKSELLLTYNPIHKKVPILVHNGNPICESSVIIEYIDETWKNESPLFPQDPYQRAKV
RFWASYIHQVLLLYSTFLLSSQIHIKQTNPYTIFLIDLYIS
>SlGSTU29
MVDVKLLGLWYSPASHKVEWALKLKGVKYEFIEENLQNKSPLLLESNPVHKKIPILIHNGKPICESMIILEYIDETFEGPSILPKDPYDRALARFWA
KFLDDKVGAMVNTFLLKGEEQEKGKKEVCEMLNVLDNELKGKKFFVGDKFGYADMAANFVGYWLGVFQEASGVVLVTSEKFSNFCVWRDEYVNCSQV
KEYLPPRNDLLAFVEARTQASASKA
>SlGSTU30
MADVKLLGLWYSFFSHRVEWALKIKGVKYEIIEEDLQNKSPLLLQSNPIHKKIPVLIHNGKSICESMIILEYIDETFEGPYILPKDPYDRALARFWA
KFLDDKVGAVVSTFIRKGEEREKGKEEACEMLKVLDNELKDKKFFVGDKFGFADIAANLVGYWLGIFQEASGVELVTSEKYPNFCAWRDEYMNCSQV
KEYLPPRNDELLAFFQGCAAAASASTQN
>SlGSTU31
MVILEYIDETFEGPSILPKDPYDRALARFWAKFLDDKVVTVVNAFLGKGEENEKAKEEVYEMLKILDNELKNKKFFVGDKFGIADIVANLVGLWLGV
FQEGSGVELVTSEKFPNFCSWRDEYVNCSQVKEYLPRRDDLLAFFQAFTRAQAAASASTQK
>SlGSTU32
MAQVKLLGFWYSPFTHRVEWALKIKGVKYEYIEEDRYNKSPLLLESNPIYKKVPVLIHNGKPICDSIVILEYIDEIFEGPSILPKDPHERALARFWA
KFLDDKVGAVVNTFLRKGEEQEKGKKEVCEMLKVLDNELKDKKLFVGDKLGFADMVANLVGLWMSVFEEASEVVLATNENFPNFCAWRNTYISCNQV
KEYLPLRIDELLAFYQDRVRALATTLATPQK
>SlGSTU33
MLKVLDNDFKDKKLFVGDKFGFVDIVANLVELWMGVFQEATGVVLATNENFPNFCARRDTYMNCSQVKEYLPSRIDELLV
FYQAYIRHSSYNFCFS*
>SlGSTU34
MVDEVKLLGVSGSSYSRRVEWALRVKGVKYEFIEEDLQNKSPLLLESNPVLKKIPVLIHNGKSICESMVIVEYIDETFEGPSILPKDPYDRAIARFW
ATFLDGMCLDAVRKGLWSKREEKEKNIQEEAYEMLKIVDNELKDKKFFSGDKIGFVDVAANYIPFWVEIVEEATGNVLITSEKFPNLCAWIDKYLKC
SEVQENLPDRDMMLSFFKAKALAEIGAK
>SlGSTU35
MADVKLLGLWYSPYSHRVEWALKIKGVEYEFIEEDLRNKSPLLLESNPIYKKIPVLIHNGKPICESMVIVEYIDETFEGPSILPKDPYDRAIARFWA
KFFDEKGSSVGRSFFLKGEEQEKAKEELHEMLKVVDNELKDKKYFVVDKFGFVDIVANVVALWLGVLEEASGVVLVTNEKYPNFYAWRDEYINCSEN
KKYLPSRNELLAKFKARILAASVAE
>SlGSTU36
MEEVKLLGLWYSPFCHRVEWALKVKGVKFEFIEENLQNKSPLLLESNPIHKKIPVLIHNGKSICESMVIVEYIDETFEGPSILPKDPYDRVIARFWV
KFFEDKGSAVGTSFFHKSEKAKEEVCEMLKILDNELKDKKFFVGDKFGFADIAANFLALWMGILEEATGIILVTKEKYPNFYAWRDEYINGNKEYLP
SRDELLAFFKARFQAAATPPYSN
>SlGSTU37
MGDVKLLGLWYSPFSHRVEWALKFKGVQYEFIEQDLQNKSPILLESNPIYKKVPVLIHNGKPICESIVILEYIDEVFEGPSILPKDPYNRALARFWV
KFFEDKGPSMRKSILLKGEEQEKAKEEVFEMLRILDNELKGKKFFVGDKFGFVDIVANAGALWLGVLEEVSGVVLVTKEKFPNFCVWRDEYCTQNKE
YLPSRDELLIRFKTYI
>SlGSTU38
MADIKLLGLWYSPFSKRVEWALKTKGVEYEYIEDDLQNKSLLLLQSNPIHKKVPVLIHNGKPICESSVILEYIDETFEGPSILPKDPYDRALARFWA
KFFEDKWPSMMKSLFFKGEEQEKGKEEVNEMLKILDNELKDKKFFVGNNFGFVDVVANAVALWFGVLEEVIGVVSVTSEKFPNFCDWRDEYYIQNKE
YLPSRDELFAHYQAYIQRVAASK
>SlGSTU39
MAEVKLLGLSYSPFNHRVEWALKIKGVKYEFIEEDLQNKSSLLLESNPIHKKIPVLIHNGKPICESMVILEYIDEAFEGPSILPKDPYDRALARFWA
KYVDDKGSAVWKSFFFKGEEQEKAKEEAYEMLKILDNEFKDKKYFVGDKFGFADIVANGAALYLGILEEVSGVVLATSEKFPNFCAWRDEYCIQNKE
YFPSRDELLIRYRAYIQPVDASK

>SlGSTU40
MADVKLIGLWYSPFSRRVEWALKIKGVEYEYIEDDLHNKSLLLLQSNPIHKAVPVLIHNGKPLCESSVILEYIDETFEGPSILPKEPYDRSLARFWA
KFFDDKGLAIRKSIFFKGEEQEKAKEEVYDMLKVLDNELKNKKIFVGEKFGFVDIVANAAALWLGVLEEASGVVLVTREKYPNFCDWRDEYCTQNKK
YLPPRDELLAHYQVYIQRVTTSK
>SlGSTU41
MVIVEYIDKTFEGPSIIPKDPYDCAIARFWAKFLDDKMPPVGKSFFLKGEEQERAKEEAYEILKILDNELKGQEVLCW*
>SlGSTU42
MGDVKLLGLWYSPFSHRVEWALKIKGVQYEFIEQDLQNKSPLLLESNPIHKKIPVLIHNGKSICESMVIVEYIDETFEGPSILPEDPYDRALARFWV
KFLEDQIAAVGKSIFLKGEEQEREKKAACEMLKILENELKDKKFFVGDKFGLADIAANVLAIWLGVFEEASGVALVTSENYPNLYGWRNEYCNQNKE
YLPSRDELLIHFQPRFPAKAK
>SlGSTU43
MAGVKLLGISLSPFSRRVEWALKIKGVEYEFVEEDLHNKSPVLLELNPIHKKIPVLIHNGKPICESMVIVEYIDETFEGPSILPKDPYDRAIARFWA
KFFDDKCMPVMGKAIFGSGEESNKAKEELGDLIKILENELKDKNFFVGDKFGFADMAGNLMAYWMGIVEEASGNIFVTSEKFPIFCNWRNEYVNCST
IKEYLPPRDEILAHFKARFAAAQK
>SlGSTU44
MGKIKLLGVSLSPFTHRVEWALKIKGVEYELIVEDPQNKSPLLLEYNPIHKKIPVLIHNGKPICESMVIVEYIDETFEGPSILPKDPYDRATARFWA
KFLDDKCLPTMGKALLGNEEEKEKAKEECGELLKILDNELKDKEFFVGDKIGFVDIAANALAFWMGIIEEASGVILVKNEKFPNYYTWRDNYINCSQ
VKKYLPSRDELFSHFQSRFHSASTTK
>SlGSTU45
MLGMKQEVYTQGIEYEFIEAQRPIKKCPNIIKYNPIYKKVPVFLHKGNPIPESLVILEYIDENWKDGTSLLPKDPYQRAIARFWAKFIDEKCLPEIL
KLCYDSNYEVKVKAMGELQELLKLLENELMKDNNKIFFGGENKVGYMEIVSILITYWLGVMQEALGVDILNKKEFPNICGWADKVISFSFMKENLPP
REKLLAIYKEYAQPLVPPNNEINHTHK
>SlGSTU46
MEEQVKLFGAFPSPFSHRIIWALKHKNISYEYIEEDLSNKSQHLLTYNPIYKMIPILVHNEKPIVESTIILEYIEETWPQNPLFPKDPYEKAKARFW
IKFGEDKNSEFHQIFHKIGEEQVKATENAKKILKIIEEQGLGDKKFFSGDTIGLIDIAFGWLAFWLEVIQEAAGVKVYEPNNFPHLQSWINNFKQVA
IIKENIPNRNAMLDYFKLRRDMIVAL
>SlGSTU47
MDQDLKLHGSWASPYSLRIIWALKLKGLLYEYIEEDLANKSDLLLKYNPIFKKIPILVHDGKPICESMIILEYLDQIWPNQYPLLPIDSYQRALARF
WVNYFEQKSVSLWMIFRSKGEEQEKAVKDSLEMLKIIEENAFKNQKNNIFFIGGKIGIVDISFGWICHWLKIIEDVGGVKLIEENSFPNLQNWMKKF
KEVPLIKESLPNHQKLFLPFKLIRDMLLAS
>SlGSTU48
MDEVKLHGTSYNLFTYRVIWALKLKGIPFEYIEEEHSNNGSLIMKYNPVFKRFPILFHGEKVISESMVIIEYIEDTWPQNPLLPIDPLDRSIARFWV
KFAGDKGACVGTMYYTSGEKQEKAIKETMEMLKIIEEQAFIEDEENIFFGGEKIGIVDLAFGVIPHWLEIIEDIIGVKLLEPNLFPNLLNWVQNFKE
EQIIKENLPNYEEMFVFLKNPKKMKLSSS
>SlGSTU49
MANEEVILLDFWPSMYGMRVRVALAEKCVNFEYKEQNMIEKSPILLEMNPIYKKIPVLIHNGKPICESLNVVQYIDEVWKNKVIFLPSDPYEKYQAM
FWADYVEKVFDTGRKLWMEKGGEKQTRKGNYIDTLRMLEGIIGDKLYFGGEKFGYLDICLIGICSWFYTYEKFGEFSTEVETPKIIAWMKRCMKRES
VYKNVVEPLKVYDFALQLRKHYGIE
>SlGSTU50
MENDEVILLDFWPSMFGMRVRVALAEKAIEYEYKEEDLFTSKSPLLVKMNPIHKKIPVLIHNGKPVCESFVVVEYIDEVWKDKAPLLPSHPYDRSQA
RFWASYTDKLYDFGRRIWTVKREEFAEGKKDFIDPLKLLEEAALGDKPYFGGESFGFVDIALIGFYSWFYTYETICNFSIEAECPKIAAWGKRCMKR
ESVSKSLADSRKIYEVVIEFRKKNGLE
>SlGSTU51
MSRVKLLGVYGSPASQRVEWALKIKGVKYEFITEDLQNKSPLLLKSNPVYKKIPVLLHNDKPIAESLVIIEYIDEAFEGPSILPKDPYDRAIARFWV
KFLDEKCLPAVWKALWSQGDEQEKDKEEAYEVLKVIDNELKDKKFFGGDNIGFVDVVANFVGFWIGIVEEATGVVLVTSENFPNFCAWRDEYLNCDR
VKENMPSREMLLGYFKSRVQAVAAISK
>SlGSTU52
MEEEVILLDFWCSMYGMRARIALEEKGVKYEYKEEDLKNKSPLLLQMNPIHKKIPVLIHNGKSICESLVIIQYIDDVWKDIGPLLIPKDPYDKAQAW
FWSDYMDNTVHEYARKTWATKGEEQEQAIKDFLGGLKLLEGVLGDKPYFGGENFGFLDVSLIGYYSWFLAYETFGKFNVELECPKLISWVKRCMERE
SVSKALPDSKKVCEFVLHLRNKIGLE
>SlGSTU53
MSNEVVLLSAYVSMFGMRVRIALHEKGIQYEYKEEDLSNKSELLLQMNPIHKKIPVLIHNGKPICESLIIVEYIDEVWKDKSPLMPFNPYKRAQARF
WADFIDKKVYDSGKRIWATKGEDQEAAKKEFIEYLKLLEGELGDKTYFNGENFGFVDLALIPFYSWFPTFEKFGNFNIEKECPKFVAWANKCIYKDS
VSKSLAESNKVYEYVLKMKQHLGLP
>SlGSTU54
MTQEEEVVLLDYWASPFGTMARIALVEKGVNFIHKFEDLSNKSPLLLEMNPVHHKIPVLVHKGKSICESNIIIQYIDEIWKNNSPLLPYEPYQRAKA
RFLVDFINKKVHGSSVKVWMGQIEEQENGKKELVECSKFLEEELGDKLYFGGDVFGFVDIALVPFYNWFIVFKTFANFNTIEIQCPKLVMWGERCLN
RDSVSKSLPTSNQVYQAYLDFKKGW
>SlGSTU55
MAELTLLGLCYNPFSHRVEWALKIKGVKYEFIEEDLRNKSLLLLKSNPIYKKIPVLIHNGKCICESMVILEYIDEAFEGPSILPKDPYDQALARFWA
KYVDNKVYFFSFL
>SlGSTU56
MEEENKVTLHGMWANPYVKRVELALKVKGIPYEYVEEYLMNKSELLLTYNPIHKNTWKNESPLFPQDQYQRAKVRFWASYIHQVYDCMLKVFRGKEA
LKRFYAKLSVLEDGINNFSLGITSNMNNIGMLDIMIVITLGAYKVQEEVFGFKLLEEENTPLLYSWVTTLIDLPIVKGITPPHDKVVSFLQYLKNKV
FKAPPHAS
>SlGSTU57
MEIVKLIGTPFSFFTYRVIWALKLKGINYEYIDEDMSKKSSLLVKYNPIHKKVPVLIHGDKIICESMVIVEYINETWKLNPLLSTDSYERATSRFWA
KYIEEKSHSSWNVFCYTGEKQQNAIKESLEMFKTIEENALGENNILFGGENIGFVDIAFGGYSLWMEIIEEIVGIKLLNPHNFPRINNWIKKFKEVQ
TIKDNLPNRDEMFVYMKNARGRMLASP
>SlGSTF1

MVVKVYGSAMAACPQRVMVCLIELGVDYELIHVDLDSLQQKKPDFLLLQPFGQVPVIEEGDFRLFESRAIIRYYAAKYEDKGKKLTGTTLEEKALVD
QWLEVESNNYNDLVYNMVLQLLVFPKMGHKSDLIVVQKCANNLEKVFDIYEQRLSKSKYLAGDFFSLADLSHLPSLRFLMNEGGFAHLVTQRKYLHD
WYLDISSRPSWSKVLDFMNLKKLEMLPGPPKEEVKV*
>SlGSTF2
MAIKVHGPMMSPAVMRVVATLKEKDLDFELVPVNMQAGDHKKEPFISLNPFGQVPAFEDGDLKLFESRAITQYIAHTYADKGNQLLPNDPKKMAVMS
VWMEVEAQKFDPIGSKLGFEIVIKPMLGMVTDDAVVAENEEKLGKLLDVYESRLKESKYLGGESFTLADLHHAPSLHYLSGSKVKSLFDARPHVSAW
VADILARPAWSKTIELSKQ*
>SlGSTF3
MAIKVHGPMLSPAVVRVVAMLKEKNLDFELVHVDLQNGDQKKEPFISLNPFGQVPAFEDGDLKLFESRAITQYIAHTYADKGNQLLPNDPKKMAIMY
VWIEVEAQRFEPVVSKLCYEIVIKPLLDMVTDDAIVAENEEKLSKLLDVYESRLKDSKYLGGDSFTLADLNHAPALHYLMGTKVKSLFNARPHVGAW
VANILARPAWAKSLELTK*
>SlGSTF4
MRVISCLIEKDLDFEFVFVDMAKEEHKRHPFLSLNPFAQVPAFEDGDLKLFESRAITQYIAQVYASNGIQLILQDPMKMAIMSVWMEVEGQKFEPPA
SKLTWELVIKPMIGLGSTDDVIVKESEEQLSKVLDIYETRLTESKYLGGDSFTLVDLHHIPNIYHLMNTKAKALFDSRPRVSVWCADILARPAWVKG
LEKMQK*
>SlGSTF5
MATPVKVYGPTLSTAVSRVLACLLEKNVQFHLIPVNMAKGEHKKPAYLKIQPFGQVPAYQDEDITLFESRSINRYICDKYGSQGNKGLYGTNPLEKA
SIDQWIEAEGQSFNPPSSVLVFQLAFAPRMKLKQDENLIRQNEEKLKKVLDVYEKRLGDSQYLAGDEFTLADLSHLPNIQYLVNGTDRAELITSREN
VGRWWGEISNRESWKKVVEMQTSPPPS*
>SlGSTF6
MQLYHHPFSLNSQKVRLTLEEKGIDYTSHHVNPLTGKNMDAFFFSMNPSAKVPVFQNGSHIIYDTIEIIQYIERIAEKVSSGGNNLNLSSREVIGWM
HKIQEWDSMYFTLFHVPEKYRLCVSKFLRRVIIARMAESPDLASAYHCKLRQAYDTDDKLKNADVLRRSENHLVRLLDEVELKLGETSYLAGEEFSL
ADVMLIPLLARIELLNLENEYINSRPNIADYWVLVKQRPSYKKVIGKYFDGWRRRKTLLKTWCFIRVRSVLRKY*
>SlGSTT1
MVVKVYGSAMAACPQRVMVCLIELGVDYELIHVDLDSLQQKKPDFLLLQPFGQVPVIEEGDFRLFESRAIIRYYAAKYEDKGKKLTGTTLEEKALVD
QWLEVESNNYNDLVYNMVLQLLVFPKMGHKSDLIVVQKCANNLEKVFDIYEQRLSKSKYLAGDFFSLADLSHLPSLRFLMNEGGFAHLVTQRKYLHD
WYLDISSRPSWSKVLDFMNLKKLEMLPGPPKEEVKV*
>SlGSTT2
MTLKLYVDRMSQPSRAVIIFCKLNGIDFEEIHINLSKRQQLSPEFKEINPMKQVPAIIDGRFKLFESHAILRYLACAFPGIADHWYPADLYKRAKVD
SVLDWHHSNLRRGAAGYIFNTVLAPAFGLPLNPQAAAEAEKVLLASLAKVESVWLQRKGRFLLGSGQPSIADLSLVCEIMELEILDEKDRERIIGPY
KRVLKWIDDTKNAMEPHFQEVHVILFKAKEKFHKQRHAVGSSIPQSSRKPDLHSKM*
>SlGSTT3
MTLKLYVDRMSQACREVIIFCKLNGIDFEEVHIDLSKRQQLSPEYREINPIRQIPAIMDGRFKLSESHAILKYLACAFPRIADHWYPADLYKRAKVE
SVLDWHRTNFPRGPGSYTFYSVLAPTVGLPLNTKAAARTEKMFIACLATIESVWLQKKGRFLLGSDQPSIADLSLACEIMQLEILDEKDRERILGPF
KRVLKWLDDTKNAMAPHFEEVQSTLAGYKEKVQKQRNTLGSKITQSGRKPVLQSNM*
>SlGSTT4
MSLKVYVDRLSQPSRAILIFCKLNGIEFEEVNIDLAKGQHRTPEYQEVNIMKQVPAIVHDTFKLFESHAILRYLASAFPETADHWYPKDLQKRANVE
CVLDWHHANLRRGSAGYVFNTILAPAFGLPLNPQAAAEGKNLLSASLATIDTYWLQKDGSFLLGNSQPSLADLSLVCEIMQLQFLDEKDREGLLSPH
KNVLKWIDDVKSATAPYFDEIHATLFKVSEIFQKQRAGGASS*
>SlGSTL1
MAASSIGHQIHINVNSPILLPLRTNFSSLSFTFSNARYPLKWNHIGCPKICALPAVSIIASGSSREMLPPALDSSSEPPAIFDGTPKLYISYSCPYA
QRTWIARNCKALQEEIKLVPIDLKNRPDWYKEKVYPANKVPSLEHNNEVKGESMDLIRYIDSNFEGPSLFPDDPSKREFAEELFSYFDSFYKAVISS
LKEDKINDAIAAFDSIETALSKFVDGSFFLGSLSLVDIAYAPFIERFQPFLLEVKNYDITTGRTKLAAWIKEMNQIEGYTVTKRDPKEHLENYKRRF
LSQL*
>SlGSTL2
MAALSVQEVLPATLESTSEPPSLFDGTTRLYINYQCPYSQRVWITRNVKGLQDKINLVPIDLQNMPDWYKEKVYPQNKVPSLEHNNKMIGESLDLVK
YVDSNFEGPSLLPDDPEKRKFAEELIAYSDIFVPEVYKSFFRDAQTLAGAQFDYLEKALDKFDDGPFFLGQFSQVDIAYVPFIERFQIFMEKGINYD
ITSARPKLAKLIEEMNKLDGYKQTKVLDPEKLVEYYKNLFLKKA*
>SlGSTL3
MATPSVQEIRPASLDSTSESPALFDGTTRLYISYVCPFAQRPWIARNFKGLQDKIELVPIDLQNRPVWYKEKVYPQNKVPSLEHNNKVIGESLDLVK
YIDSNFEGPFLLPDDPEKQKFAEELIAYSDTFLKEIYANFKGDIEKHAGPQFDYLEKALDKFDDGPFFLGQFSQVDIVYAPFVERFQIFLKEGLNYD
ITSGRPKLAKWTEELNKLDSYIQTKADPKEVVDLYKKKYLA*
>SlGSTL4
MASPSVQDLLPPSLDSTSQPPSLFDGTTRLYINYQCPYSQRVWITRNVKGLQDMIKLVPIDLQNRPDWYKENVYPKNKVPSLEHNNKVTGESLVLVK
YVDCNFEGPSFLPDDQEKRKFAEELIAYSDTTFVPEVYRSFAKDARTLAGAQFDYLEKALHKFDDGPFFLGQFSQVDIIYAPFVERFHVFMPEGFNY
DITTGRPKLAKWTEEMNNLDGYKQTKVLEQEKMIEYYKNRFLPKA*
>SlGSTL5
MLLTQPVLLLSPLPFKKKQLSMASPSVQDLLPPSLDSTSQPPSLFDGTTRLYINYQCPYSQRVWITRNVKGLQDMIKLVPIDLQNRPDWYKEKVYPK
NKVPSLEHNNKVTGESLVLVKYVDCNFEGPSFLPDDQEKRKFVEELIAYSDTTFVPEVYKSFAKDARTQAGVQFDYLEKALHKFDDGPFFLGQLSQV
DIIYAPFVERFHVFMPEGFNYDITTGRPKLAKWIEEMNNLDGYKQTKVLEQEKMVGYYKNRFLLVPTS*
>SlGSTL6
MASSSVQDLLPPSLDSTSQPPSLFDGTTRLYINYQCPYSQRVWITRNVKGLQDMIKLVPIDLQNRPDWYKEKVYPKNKVPSLEHNNKVIGESFVLVK
YVDYNFEGPSFMPDDQEKQKFAEELIAYSDTTFVPEVYRSFAKDARKLAGAQFDYLEKALHKFDDGPFFLGQFSQVDIIYAPFIERFHVFMPEGFNY
DITTGRPKLAKWIEEMNNLDGYKQTKVLEQEKMVEYYKNRFLPKA*
>SlGSTL7
MGIFFYSDRSVKHLLPPSLDSTSQPPSLFDGTTRLYMNYQCPYSQHVWITRNVKGLQDMIKLVPINQQNRPDWYKEKVYPKNKVPSLKHNNKVIRES
LVLVKYVDCNFEGPSFMPDKYTDLSQMMHGNWLVRIDHVFVETHHVLFVKCFLCKLTVLTLVLGAQFDYLEKALHKFDDGPFFLGQFSQGFNYDITT
ERPKLAKWIEEMNNLDGYKQTKVLEQEKMVEYYKNRFLPKA*
>SlGSTZ1

MKINPPMLLQISRSYIKLVKADNFQLWFGRHFSNTVTPTTNSIVDVSLAVPISGSSMESDNSMKKATDSTWVSKIVLYSFWQSSCSWRVRFALNLKG
LSYEYRAVNLGKGEQFTSEFDKLNPLHYVPVLVDGDVVISDSYAILLYLEEKYHQRPLLPIKPQLRALNLQAASIVSSNMQPLHMLSVLRYMEERVG
PEEKQLWAKFHIQKGFGALEKLLTGSAGKYATGDEVYMADVFLAPQIAVATKRFDIDMSEFPTLRKIYDSCEALPEFQASLPERQPDASP*
>SlGSTZ2
MAGSGEESKKLQLYSYWRSSCAFRVRIALNLKGLDYEYKAVNLLKGEQRDPEYLKLNPLGYVPTLVDGDAVIADSFAILMYLEEKYPQRALLPQDCQ
KRAINYQAANIVSANIQPLQNLAVLKYIQEKIGPDETTPWVQGHITKGFEALEKLLKDYAGKYATGDEVYMADLFLAPQIHAAIKRFEVDMNQFPTL
LRVFEAYQELPAFQDAMPEKQPDAIHHL*
>SlDHAR1
MAVLRFPSLFYLGLLLRGSKIFSTFVSFMKSKDSSDGTEQALLDELKALEEHLKVHGPYVDGKNVCSVHMILAPKLYHLEVALGHFKKWSVPESLSH
VRNYMNDFLGSGANFPVGNVSCFLFYSRHVAAFVIAEMKIVLFI*
>SlDHAR2
MVVEVCVKAAVGAPDVLGDCPFSQRVLLTLEEKKVTYKKHLINVSDKPKWFLEVNPEGKVPVINFGDKWIPDSDVIVGIIEEKYPNPSLIAPPEFAS
VGSKIFPTFVSFLKSKDSSDSTEQALLDELKALEEHLKAHGPYINGQNVCSVDMSLAPKLYHLEVALGHFKKWSVPESLSHVRNYMKLLFERESFQK
TKAEEKYVIAGWAPKV*
>SlDHAR3
MESKDSSDCTEQALFDELKALEEHLKAHGPYVNGQNVCSVDMSLAPKLYHLKVALGHFKKWSVTESLTHVRNYMKDFVGNMSCFLFYSRDVAASVVV
EMKIVFIYLTHWP*
>SlDHAR4
MKSKDSSDCTEHALFDELKALEEHLKAHGPYVNGQNVCSVDMSLAPKLCHLEVALGNFKKWSVTESLSHVRNYMKNDIKSAFWSMLLCYIEQHVSHS
LFSSMQ*
>SlDHAR5
MSTAKITPSAASFATSIKHLAGIQLPRCQSTIFTSNSTKFRAPRRGFTVSMAASIETPLEVCVKQSITTPNKLGDCPFTQRVLLTLEEKHLPYDMKF
VDLSNKPDWFLKISPEGKVPLIKLDEKWVPDSDVISQALEEKFPKPPLTTPPEKASVGSKIFPKFVAFLKSKDSGDGTEQALLDELTAFNDYLKENG
PFINGNEVSAADLSLGPKLYHLEIALGNYKNWSIPDSLSYMKSYMKSIFSRESFINTRALKEDVIEGWRPKVMG*
>SlDHAR6
MKSKDSSDCTVQALFDELKALEEHLKAHGPYVNGQNVYSVDMSLAPKLYHLEVALEHFKKWSVTECLSHVRNYMKLDPFLLPVVQGSILVIQLNT
>SlEF1Bγ1
MALILHSTDNNKNASKALIAAEYTGVKVDLAKDFQMGVSNKTPEFLEMNPIGKVPVLQTPDGPVFESNAIARYVTKTKPNNPLFGSSLIEYAQIEQW
NDFSATEIDANIARWLYPRLGYAVYISQAEEGAVAALKRALGALNTHLASNTYLVGHFITLADIIMVCNLSIGFRMILTKSFTKEFPHVERYFWTVV
NQPNFVKILGEVKQAESIPAVQSKPAQPEKLKAKEEPKKEVKKEEPSPVEEEAAPKPKAKNPLDLLPPSKMILDDWKRLYSNTKTNFREVAVKGFWD
MYDPEGYSLWFCDYKYNDENTVSFVTLNKVGGFLQRMELVRKYAFGKMLIVGSEAPFKVQGLWLFRGKEIPKFVMEEVYDMELYEWKEVDINDEAQK
ERVSQMIEDHEPFEGQALLDAKCFK*
>SlEF1Bγ2
MDLFLKAMLSHSTVRPCLIFFHLSSHHIAKYSFDTDFTNSRSYQINAQQSSFGSSLFEYSEEAAVSALKRALGTLNTHLASTKYLVEHLITLADNIV
VCNLSIGFRMIMTKSFTKEIPRVERYFWTVVNQQNFSKILGKVKQAKSILAVQSKKPTQLEKTKANEEPTKEVNKEEPSLVEKETTPKPKAKNPLDL
LPPSKISG*
>SlEF1Bγ3
MLQILHSTNNNKNASKALIAAEYTGVKVEVPKDFQMGVSNNTPEFLKMNPIGKVPVLETPDGPVFESNAIARYVTKLKPNNPLFGSSLIEYSQIEQW
NDFSATEVDANIGRWLYPRLGFRVYIPAAEEAAVAALKRALGALNTHLASNTYLVGHSITLADIIMVCNLSIGFRMIMTKSFTKEFPHVERYFWTVV
NQPNFCKILREVKQAESIPAVQSKKPAQPEKPKAKEELKKEVKKEEPSPVEEEAAPKPKAKNPLDLLPPSKMILDDWKRLYSNTKTNFREVAVKGFW
DMYDPEGYSLWFCDYKYNDENTVSFVTLNKVGGFLQRMDLVRKYAFGKMLIVGSEAPFKVQGLWLFRGKEIPMFVMEEVYDMELYEWKEVDINDEAQ
KERVSQMIEDHEPFEGEALLDAKCFK*
>SlTCHQD
MQLYHHPFSLNSQKVRLTLEEKGIDYTSHHVNPLTGKNMDAFFFSMNPSAKVPVFQNGSHIIYDTIEIIQYIERIAEKVSSGGNNLNLSSREVIGWM
HKIQEWDSMYFTLFHVPEKYRLCVSKFLRRVIIARMAESPDLASAYHCKLRQAYDTDDKLKNADVLRRSENHLVRLLDEVELKLGETSYLAGEEFSL
ADVMLIPLLARIELLNLENEYINSRPNIADYWVLVKQRPSYKKVIGKYFDGWRRRKTLLKTWCFIRVRSVLRKY*
>SlMGST1
MAGVEFLPKEYGYVILALVVYCFFNFWMSFQVGKARKQYKVPYPTMYATEAENKNANSFNCVQRGHQNSLEMMPTFFMLMIVGGIRHPLICASLGAV
YIVSRYFYFTGYSTGDPQNRLTLGKYNFLAIMALMICAASCGVNFLMS*
>SlMGST2
MTSWAEIFPEPTAPGAQFKLLTWQSLKPSLNPKLSCLQIATKAHRIKVCIFQIFGRGKRIAGMRRVSRIAALYRAVDGAAAMEVPQHRMSTAAQFST
SSNKSSTRSNWLFNNLLTDLSARTSAHAVAGTMLFSVAATTLTEEVHAKEVVPPELRPKDLVLYQYEACPFCNKVKAFLDYYDLPYKIIEVNPISKK
ELKWSDYKKVPVVLVDGEQMVNSSDIIDKLYEKVRSGDSTFDADEESKWRKWVDDHLVHMLSPNIYRNTSEALESFDYITSHGNFSFTERITAKYAG
AAAMYFVSKKLKKKYNITDERAALYEAAETWVDALKGRDFLGGSKPNLADLAVYGVLRPIRYLKSGRDMVENTRIGDWYSRMESEVGVSARIQA*
>SlGHR1
MSATGAFERTASTFRNIVSREPGSVFPVESGRYHLYISYACPWASRCLAYLKIKGLDQAIDFTSVKPVWERTKDSDEHTGWVFASSSTEEAGADLDP
LNGAKSIRELYELASTNYSGKYTVPVLWDKKLKTIVNNESAEIIRMFNSEFNDIAENAALDLYPPHLQSLINEANEWIYDGINNGVYQCGFAKKQEP
YDEAVQKVYKALDKCEEILSKQRYICGDQVTEADIRLFVTLIRFDEVYAVYFKCNKKLLREYPNLFNYTKDIFQIPGMSSTVNMEHIKKHYYRSHPS
INPFGIIPQGPNIDYSSPHDREKFSK*
>SlGHR2
MYSAQVSSFISIPFPPSKSKTHKLKYPKILHTKLCNSTPKMSLNQNSNTNLINTITKLLWGPSLPPQLLISTVRSTWSATWQLMMSQLAPSDPTGSY
TRPTSQFRLYSNPELKFSPKDLHLYVGLPCPWAHRTLIVRALKGLEDSVPVSIASPGIDGSWEFRVFSDPDKDKLVPGLDKANGCKTLREVYKLRRG
GYSGRSTVPMLWDMGKKEVLCNESYDIIEFFNSGLNEISGNPELDLSPPALKVDIRKWNDIIYPNVNNGVYRCGFAQSQEAYNKAAEGLFRTLEMLE
DHLAGSRYLCGDVLTLADVCLFTTLIRFDVVYNVLFKCTKKKLIEFTNLHGYLRDIYQIPKVAETCNMGQIMEGYYKILFPLNPGGINPIMPSGCED
EVLSKPHNRDCLSLETKVVQHSVS*
>StGSTU1
MEAITNIKVLGTSTSPFSNRVEIALNIKSLDYEFIVEDNFNNKSELLLQSNPIHKKIPILIHGDKAMCESLVILQYIDETWPDGLSILPSDPYDCAI
ARFWATYVDNKWFPLMLEIREAMGKDAKKVVLQNIIEGLDQLEEAFVKCSKGKDFFGGDNIGYVDIVLGCFLGWIRGMEMMLGLNLVYEAQTPSLAK
WAERFLSEKVVKDVILEHGVLDFLL*
>StGSTU2

MGEVNVHGIFAGPFNKRVELALKLKGVKYEYIEEDRSNKSDELVKYNPIYKQVPVLVHNGKPICESIIILEYIDDTWESNTIPLLPKNPYQRSMARF
LAKLIDEKLMGAMYKVCYGKGEEKEKGCDETLEVLKYFDNELQNKKFFGGDNLGFIDIVASYIALWFGAIQEAIGMELLTEQKFPKLSKWIDEFLSS
RIVKENLPTREVLVPLYKAQFAAATQKVSS*
>StGSTU3
MTGEKVKLLGNWASPSALRVHWALKLKGIEYDYQEEDLRNKSPLLLQYNPVHKKIPVLVHNGKPIAESLVILEYIEETWKHNPLLPEDPYERAKARF
WAKFVDDKCVPGIFGTFVKVGEEQQKIAKEARENLKILEGELGKKHFFGDTKIGFMDVASAWIICWAQIVEEIVDIKLIDAEEMPSLVSWFQNVLEA
APILKECTPPKDKLLEHNKGFHKMLVASASP*
>StGSTU4
MADEVKLYRTWSSRFSLRIVWALHIKGIEYEAIFEDLSHKSPQLLQYNPVHKKVPVLVHQGKPICESLVILEYIDETWKKETTPLLPQDPYEKAMAR
FWAKFVDDKTTSN*
>StGSTU5
MADEVKLYRTWSSPYGLRIVWAMHIKGIEYENVFEDLSQKSPQLLQYNPVHKKIPVLVHKGKPICESLVILEYIDETWKETTPLLPQDPYEKAMARF
WAKFVDDKLLPSVWSVFTEKGYEAKKEALVPAVQNLEIIEEQLKEKKFFGGESIGYVDLALGWVAYLLDVFEEVIDLKLFDAHKFPLLSGWMKNFCD
APAIKQHLPPRDKLVTRFQLFHEKFQTAN*
>StGSTU6
MEDVKLLGTKESIFTQRIMWALKLKGICYEFIEQDFSSRSSPLLVKLNPVYNKVPVIVHDGNSLAESLVILEYIEETWPLINPLFPVDPFERASGRF
WARFVDGKFYEAAKRAFFSSGETKAEGVESVVEGLHLLEGQIIGKKFFGGEKIGYLDIIIGWIAYWFQYIEEIGEFKAMDSTKYPCLHAWINNFIQL
PIIQQSLPTPDVVKAVFRGFKDAAALAGAN*
>StGSTU7
MVDEVVLLGTYVSMFAVRVKIALADKGIQYEYKEENLVNKSPLLLQMNPIHKKIPVLIHNGKPICESLIIVEYIDEVWKDKSPLLPSDPYKKAQARF
WADYVDKKIYDGGKKIWTTKVEDQEAANKEFIESLKVLEEELGDKPYFDGESFGFVDLALIPYYSWFPAYEKFGKFSIEPECPKFVAWAKRCMQKEN
VSKYLSDPDKIYDFVVMLRQRMGIA*
>StGSTU8
MANDEVILLDFWPSMFGMRLRIALAEKEIKYEYKDEDLRNKSPLLLQMNPIHKKIPVLIHNGKPICESIIGVEYIDEVWKDKAPFLPSDPYERAQAR
FWADYVDKKLFDSGRKLWTTKGEEQEAAKKDFIECLKVLEGALGEKPYFGGDNFGFVDIALIGFYSWFHSYETYGNFSTEAECPKFVAWAKRCMQRD
SVAKSLPDQHKVLDFVKTLRHKFGIE*
>StGSTU9
MANDEVIVLGFWPSMFGLRVRIALAEKEVKYEYREEDLKNKSPLLLQMNPIHTKIPVLIHNGKPICESIIGVEYIDEVWKDKAPLLPSDPYERAQAR
FWADYINKKFYWPARKLWTTKGEEQETAKKDFIECLKVLEGELGEKPYFGGDNFGFVDITLIGFYCWFSAYETYGYFSTEAEFPKFFAWAKRCMQRD
SVAQSLPDQHKVLEYVKPVRQRLGIE*
>StGSTU10
MVNDEVILLDFWPSMFGMRLRIALAEKEVKYEYKEEDLWNKSTLLLEMNPIHKKVPVLIHNGKPICESIIGVEYIEEVWKDKTPLLPSDPYERAQAR
FWADYINKRYETYGSFSIEAECPKLVAWAKRCMQRDSVANSLPDQHKVCEFVSVARQKLGIE*
>StGSTU11
MFGMRLRIALAEKEIKYEYKEEEGLITNKSSLLLEMNPIHKKVPVLIHNGKPICESIIGVEYIEEVWKDKAPLLPSDPYERAQARFWADYIDKKLYI
SSRKIWGTKGEEQEAGKKDFIEVLKVLEGELGEKPYFGGDNFGFVDIALIGFYSWFYAYETCGNFSTEAECPKFVAWAKRCMQRDSVAKSLPDQHKV
LEFIQMLRQKFGIE*
>StGSTU12
MSKDELRLLDFWASPFCMRVKIALSEKGVAYESQQEDLFGGKSDVLLKSNPIYEKVPVLLDNGKPIVESNNIVYYIDDKYPSPNPLLPSCAYGRSRA
RFWADFIDKKIFEAGMGIWKNKGEELEIAKKDFIEILKKLEGAMGDKDYFGGDNFGYVDVIAIAMTSWFHAYEVFGDFKVEQECPKFGCWMKRCLKR
ESVSSVLPDPEKIYQCVIMLRKMHGIE*
>StGSTU13
MGEENKVTLHGMWLSPYVKRVELALKVKGIPFEYVEEDLSNKSPLILKYNPIHKKVPILFHNGKPVNESFVIV
>StGSTU14
MEEENNKVTLHGMWISTYAKKVELALKIKGITFDYVEEDLSNKSSLLLKYNPIYKKVPLLLHRGKPVSESLVILEYIDETWNNLRPLLLPEDPYERA
TVRLWASYCLQISDTMKKAFISATDVEGGAFDEFFEILKVMEEGMKDFFPGGRSKICAENLGLLDIIIVCSLAAYKAAEEVVGMKILDPEKNPLVYS
WVTTLLELPLVKETLPPHDKVVSRLEFIKKNGYRFQSNN*
>StGSTU15
MSNKSELLLKSNPVHKKIPVLIHGDNTICESLVIVQYIDETWTNGPSILPSNPLDRAIARFWVAYIDDKWLPLMSDLGKAQGEEAKLEVQEKLQEAL
VPLEEAFVKCSKGKSFFGGDNIGYIDITLGCILGWIKAIKIMLGIEIFNVTKTPGLVNWGDRFLEDKFVKGAMLEPEKLVEILKLHLAKREANNAN*
>StGSTU16
MSNKSELLLKSNPVHKKIPVLIHGDNTICESLVIVQYIDETWTNGPSILPSNPLDRAIARFWVAYIDDKWLPLMSDLGKAQGEEAKLEVQEKLQEAL
VPLEEAFVKCSKGKSFFGGDNIGYIDITLGCILGWIKAIKIMLGIEIFNVTKTPGLVNWGDRFLEDKFVKGAMLEPEKLVEILKLHLAKREANNAN*
>StGSTU17
MATNCVKLLGVWASPYVNRVEIALKMKSIEYEFIQEHVLNKSELLLKSNHVSKKIPVLFHDEKPICESLWYPLVAEYRNAKGKEAKAAVVEKMSEGN
LLLEEAFMKISKGKSYFGGDSIGYVDIVLGSLLGWVKVIEIMDEMKILDETKTPSLAEWAERFCSDNVVKDIIPQTEKLVGIYRKYLEVK*
>StGSTU18
MATGSVKLIGSWASPFVNRVEIALKINSIDSELIQENVLNKSELLLKSNPVYKKIPVLIHNEQPICESLVFLQYIDEAWLNGPSILPSDPYDRAIAR
FWAVYIDEKVKT*
>StGSTU19
MAENDVKLLGSWPSTFVVRPRIALNVKSVCYDFLEEQLSSKSDLLLKSNPVYKKIPVLIHDGKSICESLNIVQYIDEKWTNSGPSILPLDPYDRAIA
RFWACYIDDKWFPIFRCLAVAQGEDAIKAALEPVFDGLVLLEDAFKNCSKGKKFFGRDKIGYVDIALGCFLGWMKVTEKMNNVTLLDEAKTPGLYKW
AEDFCADSSVKDVMPETNKLAEAAKDLIPKIRANASS*
>StGSTU20
MEEVKLLGFWVSPFSIRVEMALKLKGIEYEYIEAQLPIKKCPKIVKYNPIYKKVPVFFHNGKPIPESLVILEYIDENWEDGTSLLPKDPYQRAMARF
WAKFMDEKCLPEIIKFCYDSNYEVKVKAMGELQELLKLLENELIKDNNNIFFGGENKVGYMEIVSILITYWLGVMQEALEVDILNKKEFPNICEWAD
KLMSCSFMKENLPPREKLLAFYKEYAQPLVLPNNELSQ*
>StGSTU21
MGEVKLIGSSGSLFCTRVEWALKLKGVDYEYIQEDLLNKSELLIKSNPVHKKIPVLLHDDKPVVESLVILEYIDETWKGYSLLPQDPHERATARFWA
KFVDDKCVIGSWQAMAMQDEGEAKAKTIESVQELYAFIEKQIEGKKFFGGEQIGYLDLVMGWKTLWLSAMEEVGNVKLLDPEKFPSLHQWAENFKEI
PIIHESMPQQETLVNYFQGGLNYLRSLETNKP*

>StGSTU22
MANDEVILLDFWPSMFGMRLRIALAEKEVKYEYKEEDLRNKSPLLLQMNPIHKKIPVLIHNGKPICESLIGVEYIDEVWKDKSPLLPSDPYVRAQAR
FWGDYIDKKLYDTARKIYSATGDEHEAGKKDFIEILKVLEGALGEKPYYGGDNFGFGDIALIGFYCWFPAYEIYGNFSIEAECPKLVAWAKRCMKRD
SVAKTLPDQHKILEFAKILREKLGLE*
>StGSTU23
MYVQQTNQYFGNFENFFFLILLNLLIHTKKKANDEVILLDSWPSMFGMRLRIALAEKEIKYEYKEEDLWNKSPLLLQMNPIHKKIPVLIHNGKPICE
SLIGVEYIEEVWKDKSPLLPSDPYERAQARFWGDYIDKKLYDTSRKIYTTTGDEQEAGKKDFIEILKVLEGALGEKPYFGGDNFGFGDIALIGFYCR
FHAYEIYGNFSIKAECPKLVAWGKRCMQRDSVAKTLPDQHKILEFIQMLRQKLGVE*
>StGSTU24
MANDELILLDFWPSMFGMRLRIALAEKEIKYEYKEEDLWNKSPLLLQMNPIHKKIPVLIHNGKPICESIIGVEYIEEVWKDKSPLLPSDPYDRAQAR
FWADYIDKKLYYSARKIWGANGEEQEAGKKEFIECLKVLEGALGEKPYFGGDNFGFVDIALIGFYSWIHSCETYGNFSTEAECPKFVVWAKRCMQRD
SVAKSLPDQHKVLEFAKTLRHKLGLE*
>StGSTU25
MANDEVILLDFWASMFGMRLRIALAEKEIKYEYKEEEGLITNKSSLLLEMNPIHKKVPVLIHNGKPICESIIGVEYIEEVWKDKAPLLPSDPYERAQ
ARFWADYIDKKLYVSSRKIWGTKGEEQEAGKKDFIEVLKVLEGELGEKPYFGGDNFGFVDIALIGFYSWFYAYETYGNFSTEAECPKFVAWAKRCMQ
RDSVAKSLPDQHKVFEFIQMLRQKFGIE*
>StGSTU26
MGDEVVLLDFWPSPFGMRVRIALKEKGIDYESKEENLSNKSSLLLKMNPVHKQIPVLIHNGKPICESLIIVQYIDEVWKDKGPLLPFDPYERSHARL
WADYVDKKIYSTGRLVWATKGEVQEAAKKEVIDHFKLLETELGDKTFFGGDQFGLVDIALIPFYSWFYALETCGNFSMIHECPKLVGWAKRCMERES
VSTSLPDQYKVYDFILELKKKLDLD*
>StGSTU27
MSDEVVLLDTFVSVFGMRVRIALAEKGIQYEYKEEDLMNKSHLLLQMNPIHKKIPVLIHNGKPICESLIIVEYIDEVWKDKSPPLMPSDPYKRAHAR
FWADYIGKKIYDGGMKIWSSKVEEHKTANKEFIECLKVLEGELGDKPYFEGENFGLVDMAFIPYYSWFPVYKKLSNLNIEAECPKFVAWAQRCMQKE
SVSKSLVDPDKVYEFIVLFRQKIGVA*
>StGSTU28
MVDVKLLGLWYSPASHKVEWALKLKGVKYEFIEENLQNKSPLLLESNPVHKKIPVLIHNGKPICESMIILEYIDETFEGPSILPKDPYERALARFWA
KFLNDKVGAAVNTFLHKGEEQEKGKEEVFEMLKILDNELKDKKFFVGDKFGYADIAANFVGYWLGVFQEASGVELVTSENFPNFCAWRDEYVNCSQV
KEYLPPRNDLLAFIQAYAQASASKT*
>StGSTU29
MADVKLLGLWYSFFGHRVEWALNIKGVKYELIEEDLQNKSPLLLQSNPIHKKIPVLIHNGKSICESMVIVEYIDETFEGPSILPKDPYDRALARFWA
KFLDDKVGAVVSTFIRKGEEQEKGKEEACEMLKVLDNELKDKKFFVGDKFGFADIAANLVGYWLGIFQEASGVKLVTSENFSNFCAWRDEYINCSQV
KEYLPPRNDELLAFFQGCARAHATASASTQK*
>StGSTU30
MAQVKLLGFWYSPFTHRVEWALKIKGVKYEYTEIEQDKKNLPRLEDYPSYKQVPVLIDNDRSICGSMVILEYIAEKFGRTSILPDDFCDRALARFWA
NFLHNKMETVRNAFLGKGKVQEKSIVEVCEMLKILGDKLEGKTFFVANEFGFADIVANMVGFWLGVYQEVSGVELVTEKKFPIFCVWRDKYVNCSQV
KKYLPSRDKLIAFYQASARPQAAASASTQALSIVTGP*
>StGSTU31
MEQVKLLGFWYSPFSHIVEWALKIKGVNYEYIEEDRNNKSSLLLESNPIHKKIPVLIHNGKPICESLVIVEYIDETFEGPSILPKDPYDRALAPFWA
KFLDDKVAVVVNAFLRKAEEHEKAKEEICEMLKILDNDLKDRKLFLANKFGYADMA
>StGSTU32
MAQVKLLGFWYSLFTHRVEWALKIKGVKYEYIEEDRYNKSSLLLESNPIYKKVPVLFHNGKPICDSMVILEYIDETFEGPSILPKDSHDRALARFWA
KFLDDKVVTVVNAFLGKGEEQEKAKEEVCEMLKILDNELKDKTFFVANKFGYADIVANLVGLWLGVFQEGSGIELVTSEKFPNFCGWRDEYVNCSQV
KEYLPSRNDLLAFFQVFVRAQAAAAASASTQK*
>StGSTU33
MATNLVGLWMGVFEEASGVVLATNEEFPNFCAWRDEYINCSQVKKYLPSRIDELLVFYQDHVRAQAPTSTTLQK*
>StGSTU34
MTQVKLLGFWYSPFSHRVEWALKIKGVKYEYIEEDRDNKSLLLLQSNPIHKKVPVLIHNGKTICESMIILEYIDETFEGPSILPKDPYDRALARFWA
KFLDNKVGAVVNTFLRKGEEQEKGKEEVSEMLKVLDNELKANKKYC
>StGSTU35
MVILEYIDETFEGPSILPKDPQDRALARFWAKFLDDKVVTVVNAFLGKGEEQEKAKEEVCEMLKILDNELKDKTFFVANKFGYADIVANLVGLWLGV
FQEGSGIELVTSEKFPNFCGWRDEYVNCSQVKEYLPSRNDLLAFFQVFVRAQAAAAASASTQK*
>StGSTU36
MAQIKLLGFWYSPFTHRVEWALKIKGIEYEYIEEDRYNKSPLLLESNPIYKKVPVLIHNGKPICDSMVILEYIDEIFEGPSILPKDPYDRALARFWA
KFLDDKVAAVVNAFLRKGEEHEKAKEEICEMLKILDNELKDKMLFVADKFGYADMAANLVGLWMGFFEEASGVVLATNEKFPNFCTWRDAYINCSQV
KEYLPSRFDELLAFYQDRVRAQAPTSATPKNK*
>StGSTU37
MAQVKLLGFWYSAFSHRVEWALKIKGVKYEYIEEDPHNKSLLLLQSNPIHKKVPVLIHNGKPICESMVILEYIDETFKGPSILPKDPYDRALARFWA
KFFDNKVVAVVNAFLGKGNEQEKGKEEVCEMLKVLEKELKDKKLFVGDKFGFVDIAANLVGLWMGVFQEASGVVLATNENFPNFCAWRDAYINCSQV
KEYLPPRIDELLAFYQAYIRTQTATSVSPKK*
>StGSTU38
MVDQVRLLGVSGSSYSRRVEWALRVKGVKYEFIEVDLQNKSSLLLESNPVLKKIPVLIHNGNPICESIVIVEYIDETFEGPSILPKDPYDRAIARFW
AKFLDGKCLEAVGKALWCKGEEQEKNIQEEAYEMLKIVDNELKDKKFFSGDKIGFVDVAANYIPFWVEIVEEATGNVLITSEKFPNLCAWIDEYLKC
SEVQENLPDRDMMLSFFKAKALAEITAK*
>StGSTU39
MADVKLLGLWYSPFSHRVEWALKIKGVKYEFIEQDLQNKSPLLLESNPVYKKIPVLIHNGKSICESTVIVEYIDEMFKGPSILPKDPYDRAIARFWG
PAVGRSFFLKGEEQDKAKEEVYEMLKVLDNELKDNKKYFVGDNFGFVDIVANAAALWLGVLEEASGVVLVTSEKFPNFCAWRDEYINCITFKR*
>StGSTU40
MEEVKLLGLWYSPFCHRVEWALKIKGVKCEFIEENLQNKSPLLLESNPIHKKIPVLIHNGKSICESMVIVEYIDETFEGPPILPNDPYDRAMARFWV
KFFEDKGSAVGTSFFHKSEKAKEEVCEMLKILDNELKDKKFFVGDKFGLADIAANFLALWMGILEEATGIILVTKEKYPNFYAWRDEYINGNKEYLP
SRDELLAFFKARFQAAATPPYSN*
>StGSTU41

MADVKLLGLWYSPFSHRVEWALKIKGVKYEFIEEDLQNKSPLLLESNPIHKKIPVLIHNGKPICESIVILEYIDEAFEGPSILPKDPYDRALARFWV
KFFEDKGPTMRKSIFLKGEEQEKAKEEVF*
>StGSTU42
MADVKLLGLWYSPFSKRVEWALKIKGVEYEYIEDDLQNKSLLLLQSNPIHKKVPVLIHNGKSICESSVIVEYIDETFEGPSILPKDPYDRALVRFWD
KFFEDKGPSMMKSLFLKGEEQEKAKEEVYEMLKILDNELKDKKFFVGDKFGFVDIVANAVALWFGVFEEVTGVVLVTKEKFPNFCVWRDEYYIQNKE
YLPPRDELFAHYQAYIQRVAASK*
>StGSTU43
MAEVKLLGLSYSPFSHRVEWALKIKGVKYEFIEEDLQNKSPLLLQSNPIHKKIPVLIHNGKCICESMVILEYIDEAFEGPSILPKDPYDRALARFWA
KYVEDKGAAVWKSFFSKGEEQEKAKEEAYEMLKILDNEFKDKKCFVGDKFGFADIVANGAALYLGILEEVSGVVLVTSEKFPNFCAWRDEYCTQNEE
YFPSRDELLIRYRAYIQPVDASK*
>StGSTU44
MSNVKLIGLWYSPFSKRVEWALKIKGVEYEYIEDDLQNKSSLLLQSNPIHKAVPVLIHNGKPLCESSVILEYIDETFEGPPILPKDPYDRALARFWA
KFFEDKGQAIRKSIFFKGEEQEKGKEEVYEMLKVLDNELKDNKKYFVGDKFGFVDIVANAAALWLGVLEEISGVVLVTREKYPNFCGWRDEYCTQNK
EYLPPRDELLAHYQVYIQRVTASK*
>StGSTU45
MVIVEYIDEAFEGPSILPKDPYDRAIARFWAKFLDDKMAPVGKSFFLKGEEQEKAKEEAYEILKILDNELKDKKLFVGDKFGFADIAANFVGIWVGV
FEEASGEVLVTKENFPNLCAWRDEYINCSENKEYLPPRNELVAHFQARFQAAPK*
>StGSTU46
MADVKFLGLWYSPFSHRVEWALKIKGVEYEFIEQDLQNKSPLLLESNPIHKKIPVLIHNGKSICESMVIVEYIDETFEGPSILPKDPYDRALARFWV
KFLEDQVAAVGKTIFLKGEEQEKAKKEACEMLKILENELKDKKYFVGDKFGFADIGANVLAIWLGVFEEASGVPLVTSENYPNLYAWRNEYCNQNKE
YLPSRDELLAHFQARFPAKAK*
>StGSTU47
MVGVKLLGVSLSPFSRRVEWALKIKGVEYEFVEEDLHNKSPLLLEYNPIHKKIPVLIHNGKPICESMVIVEYIDETFEGPSILPKDPYDRAIARFWA
KFFDDKCMPVMGKAIFGSGEESDKAKEELGDLIKILENELKDKKYFVGDKFGFADMAGNLMAYWLGIVEEASGNIFVTNEKFPIFCNWRNEYINCST
IKEYLPPRDAILAHFKARFEAAQK*
>StGSTU48
MTQVKLIGVTPCPFSRRVEWALKFKGVEYEFIEENLQNKSPLLLESNPIHKKIPVLIHNGKPICESMVILDYIDETFEGPFILPKDPYDLLFGKGEE
AEKSKEEFGGLLKILDNEFKDKKFFVGDNFGFADVAANFMAFWFGILEEASGVVLVTSEKFPNFCGWRDEYINRSQVKEYLPSRDVLLAHFQSRFQS
LK*
>StGSTU49
MEEIKLLGVSFSPFTHRVEWALKIKGVEYELIVEDPQNKSPLLLESNPIHKKIPVLIHNGKPICESMVIVEYIDETFEGPSILPKDPYDRAIARFWA
KFLDEKCLPAMGKALLGNEEEQEKAKEECSELLKILDNELKDKEFFVGDKIGFVDIAANVLAFWMGIIEEASGVILVTNKKFPNYYAWRDNYINCSQ
VKEHLPSRDELFSHFQSRFHSASTTK*
>StGSTU50
MVILEYIDETFDGPSILPKDPYDRALARFWAKFLDDKVTAVVNTFFRKGDEQEKGKEEVYEMLKVLDNELKDKKFFVCDKFGFADIVANMVALWLGI
YEETSGVVLVTSQKFPKFCAWRDEYINCSQVKEYLPSRRDELLTFFHARVCARAQVTTSASASK*
>StGSTU51
MEEQVKLFGVFPSPFSYRVIWALKHKNISYEYIEEDLSNKSQHLLTYNPICKMIPILLHNGKPIVESTIILEYIEETWPQNPLFPKDPYKRLRLDSG
SSSEKIR*
>StGSTU52
MEEQVKLFGAFPSPFSYRIIWALKHKNICYEYIEEDLSNKSQQLLTYNPICKMIPILLHGGKPIVESTIILEYIEETWPQNPLFPKDPYKRLRLDSG
SSSEKIRELINSEFYQIFHKIGEEQVKATENAKKIFKIIEEQGLGDKKFFSGDTIGLIDIAFGWLAFWLEVIQEAAGVKVYEPNNFPHLQSWINNFK
QVAIIKENIPNRDAMLDYFKRRREIIVTI*
>StGSTU53
MDQDLKLHGSWASPYSLRIIWALKLKGLSYEYIEEDLANKSDLLLKYNPIFKKIPILVHDGKPICESMIILEYLDQIWPNQYPLLPIDPYERALARF
WVNYFEQKSVSIWMMFRSKGEEQQKAVKDSLEMLKIIEENAFKNQKNNIFFIGGKIGIVDIAFGWICHWLKIIEDVGGVKLIEGNSFPNLQNWMKKF
KEVPLIKESLPNHQKMFLPFKLIRDMLLAS*
>StGSTU54
MEEVKLHGTSYNLFTYRVIWALKLKGIPFEYLEEEHSNKGSLIMKYNPAFKRFPILFHGEKVISESMVIIEYIEDTWPQNPLLPIDPLDRSIARFWV
KFAGDKV*
>StGSTU55
MDQDLKLHGSWPSPYSLRIIWALRLKGLSYEYIEEDLANKSDLLLKYNPIFKKIPILVHDGKPICESMIILEYLDQIWPNQYPLLPIYPYKELWLDF
G*
>StGSTU56
MEEVKLHGTSYNLFTYRVIWALKLKGIPFEYIEEEHSNKGSLIMKYNPAFKRFPILFHGEKVISESMVIIEYIEDTWPQNPLLPIDPLDRSIARFWV
KFAGDKGSCIGAMYYTSGEKQEKAIKETIEMLKIIEEQAFIGDQENNFFGGEKIGIVDLTFGIIPHWLEIIEDIIGVTLLEPNLFPNLLNWVQNFKE
EQIIKENLPNYDEMFVFLKNQREMILSSS*
>StGSTU57
MENDEVILLDFWPSMFGMRVRVALAEKAIEYEYKEEDLFCSKSPLLLKMNPIHKKIPVLIHNGKPVCESFVVVEYIDEVWKDKAPLLPSDPYERSQA
RFWASYTDKLYDFGRRVWTHKREEFAEGKKDLIDPLKLLEEAALGDQPYFGGESFGFVDIALIGFYIWFYTYETICNFSIEAECPKIAAWGKRCMKR
ESVSKSLPDSHKIYEVVIENRKKSGLE*
>StGSTU58
MANEEVILLDFWPSMYGMRVRVALAEKCVNFEYKEQNMVEKSSILLEMNPIYKKIPVLIHNGKPICESLNVVQYIDEVWKDKVTFLPSDPYEKHQAM
FWADYVEKVFDTGRKLWMEKGGEKQTRKENYIDTLRMLEGILGDKLYFGGEKFGYLDISLIGICSWFYTYEKFGEFSTEVETPKIIAWMKRCMKRES
VSKYVVEPLKVYDFALQIRKHYGIE*
>StGSTU59
MSGVKLLGVNGSPASQRVEWALKIKGVKYEFITEDLQNKSPLLLKSNPVYKKIPVLLHNENPISESLVIIEYIDEAFEGPSILPKDPYDKAIARFWA
KFLDDKCLPAVWKALWSQGEEQEKDKEEAYEVLKVLDNELKDKKFFGGDNIGFVDIVANFVGFWIEIVEEATGVVLVTSEKFPNFCVWRDEYLNCDK
VKENMPSREMLLGFFKSRVQAAAATLK*
>StGSTU60

MTQEEEEVVLLDYWPSPFGTMARIALVEKGVNYIHKFEDLSNKSPLLLEMNPVHHKIPVLVHKGKSICESNIIIQYIDEIWKNNFPLLPCEPYQRAK
ARFLVDFINKKVHGSSVKVWMGQNEEQEDGKKELVEWSKFLEEELGDKLYFGGDEFGFVDIAFVPFYNWLIVFKTFANFNTIEIECPKLVMWGERCL
KRDSVSKSLPTSDQVYQAYLEFKKGWSHG*
>StGSTU61
MSNEVVLLSAYVSMFGMRARIALDEKGIQYEYKEEDLSNKSSLLLQMNPIHKKIPVLIHNGKPICESLIIVEYIDEVWKGKSPLMPSDPYKRAQARF
WADFIDKKVYESGKRIWTTKGEDQEAAKKEFIECLKLLEGELVDKPYFNGENFGFVDLALIPFYSWFPTFEKFGNFNIEKVCPKFVAWANKCMHKDS
VSKSIAEPNKVYEYVLKLKQQLGLP*
>StGSTU62
MEEEVILLDFWCSMYGMRARIALEEKGIKYEYKEEDLKNKSPLLLQMNPIHKKIPVLIHNGKSICESLVIIQYIDDVWKGTGPLLVPKDPYEKAQAW
FWSDYMDNTVHEYARKTWATKGEEQEQSIKDFLDGLKLLEGVLGDKPYFGGDNFGFLDVSLIGYYSWFLAYETFGKFNVELECPKLISWVKRCLERD
SVSKALPDSKKVCEFVLHLRNKFGLE*
>StGSTU63
MARNDLKILGAWPSPYVMRTRIALNIKSLAYDFLEEQFGTKSELLLKSNPIYKKIPVLIHDGKPICESLIIVQYIDENWTNFGHSILPSHPYDRAIA
RFWASYIDDKWFPALRGVAATQEENAKKAATETVIEGLVLLEDVFKNCSKGKKFFGGDKIGYLDIALGCFLGWLKVNEKLNNVNLLDESKTPGLYKW
AEDFCGDSVVKDVMPETDKLAQAAKVIWAKIRAQASS*
>StGSTU64
MNHVEIALKIKSIESEFIQQNMLKKSELLLKSNPIYKKIPVLIPDEKTNCESFWFPSFRCLAISQGEDAIKATLEPVFDSLVLLEDAFKNCIKGKKF
FDRDKIGYMGIALGFFLSWMRVTEKMNNVTLLNEAKIPGLYK*
>StGSTU65
MEEENRVTLHGMWASPYAKRVELALKVKGIPYEYVEEDLMNKSALLLKYNPINKKVPILVHNGNPICESSVILEYIDETWKNESPLFPQDPYQRAKI
RFWASYIHQVYDCMLKVFRGEEQDKALEEFYAKLSVLEDGINNTSLGITTNMNNIGMLDIMIVITLGAYKVQEEVFGSKLLEAEKTPLLYSWVTTLI
ELPIVKGITPPHDKVVSFLQYLKNKVFKATPHAN*
>StGSTU66
METVKLIGTPFSFFTYRVIWALKLKGVNYEYIDEDMSKKSPLLVKYNPIHKKVPVLIHGDKTICESMVIVEYINEMWQLNPLLSTDSYERATSRFWV
KYIEEKSHNTWNVFCNTGEKQQNAIKESLEMFKTIEENALGENNILFGGENIGLVDIAFGGYSHWMEIIEEIVGVKLLDPHNFPRINKWIKNFKEVK
AIKDNLPNRDEMFVYMKNARERMLASP*
>StGSTF1
MVVKVYGSAMAACPQRVMVCLIELGVDYELIHIDLDSLQQKKPDFLILQPFGQVPVIEDGDFRLFESRAIIRYYAAKYEDKGKKLTGTTLEEKALVD
QWLEVESNNYNDLVYNMVLQLLVFPKMGHNSDLIVVQKCANNLEKVFDIYEQRLSKSKYLAGDFFSLADLSHLPSLRFLMNEGGFAHLVTQRKCLHD
WYLDISSRPSWNKVLDFMNMKKSEMLPGPPKDEVKV*
>StGSTF2
MVIKVHGPMMSPAVMRVVATLKEKDLDFELVPVNMQSGDHKKEPFISLNPFGQVPAFEDGDLKLFESRAITQYIAHTYADKGTQLLPNDPKKMAIMS
VWMEVEAQKFDPIGSKLGFEIVIKPMLGMVTDDAVVAENEEKLGKLLDVYESRLKESKYLGGESFTLADLHHAPSLHYLMGSKVKSLFDARPHVSAW
CADILARPAWCKTLELSKQ*
>StGSTF3
MAIKVHGPMLSPAVVRVVATLKEKNLDFELIHVDLQNGDHKREPFISLNPFGQVPAFEDGDLKLFESRAITQYIAHTYADKGNQLLPNDPKKMAIMY
VWIEVEAQKFEPVGSKLSYEIVIKPMLGMVTDDAIVAENEEKLSKLLDVYESRLKNSKYLSGDSFTLADLHHAPVLHYLIGTKVKSLFNARPHVSAW
VADILARPAWAKSLELTK*
>StGSTF4
MRVVSCLIEKDLDFEFVFVDMANEEHKKHPFLSLNPFAQVPAFEDGDLKLFESRAITQYIAHTYASNGIQLILQDPKKMAIMSIWMEVEGQKFEPLA
SKLTWELVIKPMIGMGSTNDDIVKESEEQLSKVLDIYETRLTESKYLGGDSFTLVDLHHIPNIYHLMNTKAKALFDSRPRVSAWCADILARPAWMKG
LEKLQK*
>StGSTF5
MATPVKVYGPTLSTAVSRVLACLLEKNVQFQLIPVNMAKGEHKKPDYLKIQPFGQVPAYQDEDITLFESRSINRYICDKYGSQGNKGLYGTNLLEKA
SIDQWIEAEGQSFNPPSSVLVFQLAFAPRMKLKQDENLIRQNEEKLKKVLDVYEKRLGDSQYLAGDEFTLADLSHLPNIQYLVNGTDRAELFTSREN
VGRWWGDISNRESWKKVVEMQTSPPPS*

>StGSTL1
MATPRSVQQIRPASLDSTSEPPALFDGTTRLYISYICPFAQRAWITRNFKGLQDKIELVPIDLQNRPVWYKEKVYPQNKVPSLEHKNKVIGESLDLV
KYIDSNFEGPSLLPDDPEKQKFAEELIAYSDTFLKEIYGNFKGDIEKHAGPQFDYLEKALDKFDDGPFFLGQFSQADIVYAPFVERFQIFLKEVFDY
DITSGRPKLAKWIEELNKLDSYIQTKADPKEVVDLYKKKYLVVTHMHA*
>StGSTL2
MAASSIGYQIHINVNSPILLPLRTNFSSLSFTFSNAKYPLKWNHIGCPKICALPAVSIMASGSSREILPPALDSSSEPPAIFDGTPKLYISYSCPYA
QRTWIARNCKGLQEEIKLVPIDLKNRPDWYKEKVYPANKVPSLEHNNEVKGESMDLIRYIDSNFKGPSLFPDDHSKREFAEELFSYFDSFYKAVISS
LKEDKINDAIAAFDSIETALSKFVDGSFFLGSFSLVDIAYVPFIERFQPFLLEVKNYDITTGRTKLAAWIKEMNQIEGYTVTKRDPKEHLENYKRRF
LSQL*
>StGSTL3
MELPGCISVINAPMLNVCGLPGMLRFGLQDKINLVPIDLQNRPDWYKEKVYPPNKVPSLEHNNKVIGESLDLVKYIDSNFEGPSLLPDDPEKQKFAE
ELIAYSDTFVPEIYRSFMRDAQTLAGAQFDYLEKALGKFDDGPFFLGQFSQVDIAYVPFIERFQIFIPAGFNYDITSGRPKLAKWIEEMDKLDGYKQ
TKVLEPEKLVEYYKNLFLKA*
>StGSTL4
MGLFFYLHRIVQEVLPPTLDSTSQPPSLFDGTTRLYINYQCPYSQRVWITRNVKGLQDKIKLVPIDLKNRPDWYKEKVYPTNKVPSLEHNNKVIGES
LDLVKYVDSNFEGPSLLSDDPEKRKFAEELIAYSDTTFVPDVYRSFAKDARTLAGAQFDYLEKALLKFDDGPFFLGQFSQVDIAYAPFIERFQVFMP
EGFDYDITTGRHKLAKWIEEMNKLDGYKQTKVLEQEKMVEYYKNRFLPVLTLQILDIFHFLVLV*
>StGSTL5
MGLFFYLHRIVQEVLPPTLDSTSQPPSLFDGTTRLYINYQCPYSQRVWIARNVKGLQEKIKLVPIDLKNRPDWYKEKVYPTNKVPSLEHNNKVIGES
LDLVKYVDSNFEGPSLTPDDPEKRKFAEELIAYRAQFDYLEKALLKFDDGPFFLGQFSQVDIAYAPFIERFQVFMPEGFDYDITTGRHKLAKWIEEM
NKLDGYKQTKVLEHEKMVEYYKNRFLPE*
>StGSTT1

MTLKLYVDRMSQACREVIIFCKLNGIDFEEVHIDLSKRQQLSPEYREINPIRQIPAIMDGRFKLSESHAILRYLACAFPRIADHWYPADLYKRAKVE
SVLDWQRTTFPRGPGSYVFYSVLGTTVGMPLNTKAAARTEKNLIASLALIESVWLQKKGRFLLGSDQPSIADLSLACEIMELEVLDDKDHERILGPF
KRVLKWLDDTKNAMAPHFEEVQSTLSNYKEKVQKQRNAVGSKITQSGRKPVLQSKM*
>StGSTT2
MTLKLYVDRMSQPSRAVIIFCKLNGIDFEEIHINLSKRQQLSPEFKEINPMKQVPAIMDGRFKLFESHAILRYLACAFPGIADHWYPADLYKRAKVD
SVLDWHHSNLRRGTAGYIFNTVLAPAFGLPLNPQAAAEAKKVLLASLANIESVWLQRKGRFLLGSGQPSIADLSLVCELMELEILDEKDRERIIGPY
KRVLKWMDDTKNAMEPHFQEVHVILFKAKEKFQRQRYDVGSSIPQSSRKPEFHSKM*
>StGSTZ1
MKINPPMLLQISRSYIKLVKADNFQRCFGRHFSNTETPTTNSIVDASLAVPISASSMESDNSKKKATDSTWVSKIVLYSFWQSSCSWRVRFALNLKG
LSYEYRAVNLGKGEQFTLEFDKLNPLHYVPVLVDGDVVISDSYAILLYLEEKYHQRPLLPVEPQLRALNLQAASIVSSNMQPLHMLSVLRYMEERVG
PEEKQLWVKFHIQKGFGALEKLLTGSAGKYATGDEVYMADVFLAPQIAVATKRFNIDMSEFPTLRKIYDSCEALPEFQASLPERQPDAPP*
>StGSTZ2
MAGSGEESKKLQLYSYWRSSCAFRVRIALNLKGLDYEYKAVNLLKGEQRDPEYLKLNPLGYVPTLVDGDAVIADSFAILMYLEEKYPQRALLPQDCQ
KRAINYQAANIVAANIQPLQNLAVLKYIHEKVGPNETTPWVQSHITKGFEALEKLLKDYAGKYATGDEVYMADLFLAPQIHAAIKRFEVDMNQFPTL
LRVFEAYQELPAFQDAMPEKQPDATC*
>StDHAR1
MSVCTHLEGSKLFPTFVSFLKSKDSSDGTEQALLDELKALEEHLKAHGPYANGQNVCSVDMSLAPKLYHLEVALGHFKKWSVPESLSHVRNYMKLLF
ERESFQKTKAEEKYVIAGWAPKV*
>StDHAR2
MAVEVCVKAAVGAPDVLGDCPFSQRVLLTLEEKKVTYKKHLINVSDKPKWFLEVNPEGKVPVINFGDKWIPDSDVIVGIIEEKYPNPSLIAPPEFAS
VGSKLFPTFVSFLKSKDSSDGTEQALLDELKALEEHLKAHGPYANGQNVCSVDMSLAPKLYHLEVALGHFKKWSVPESLSHVRNYMKLLFERESFQN
TKAEEKYVIAGWAPKV*
>StDHAR3
MSTVKITPSAASFATSIKHLAGIQLPRLQNTIFTSNSTKFRAPRRAFTVSMAASLDTPLEVCVKQSITTPNKLGDCPFTQRVLLTLEEKHLPYDMKF
VDLSNKPDWFLKISPEGKVPLIKLDEKWVPDSDVITQALEEKFPEPPLTTPPEKASIGSKIFPKFVAFLKSKDPTDGTEQALLDELTAFNDYLKENG
PFINGNEVSAADLSLGPKLYHLEISLGHYKNWSIPDSLSYVKSYMKSIFSRESFINTRALKEDVIEGWRPKVMG*
>StEF1Bg1
MALILHSTNNKNTSKALIAAEYTGVKVDLAKDFQMGVSNKTPEFLEMNPIGKVPVLQTPDGPVFESNAIARYVTKTKPDNPLFGSSLIEYAQIEQWN
DFSATEVDANIARWLYPRLGYGVIPQAEEGAVAALKRALGALNTHLASNTYLVGHCITLADIIMVCNLSIGFRMIMTKSFTKEFPHVERYFWTVVNQ
PNFCKILGEVKQAESIPAVQSKMPAQPEKPKAKEEPKKEVKKEEPSPVEEEVAPKPKAKNPLDLLPPSKMILDDWKRLYSNTKTNFREVAVKGFWDM
YDPEGYSLWFCDYKYNDENTVSFVTLNKVGGFLQRMDLVRKYAFGKMLIVGSEAPFKVQGLWLFRGKEIPKFVMDEVYDMELYEWKEVDINDEAQKE
RVSQMIEDHEPFEGQALLDAKCFK*
>StEF1B g2
MLQILHSTNNNKNASKALIAAEFTGVKVELAKDFQMGVSNNSPEFLKMNPIGKVPVLETPDGPVFESNAIARYVTKLKPNNPLFGSSLIEYAQIEQW
NDFSATEVDANIGRWLYPRLGYRVYIPAAEEAVVAALKRALGALNTHLASNTYLVGHLITLADIIMGCNLSIGFRMIMTKSFTKEFPHVERYFWTVV
NQPNFCKILGEVKQAESIPAVQSKMPAQPEKPKAKEEPKKEVKKEEPSPVEEEVAPKPKAKNPLDLLPPSKMILDDWKRLYSNTKTNFREVAVKGFW
DMYDPEGYSLWFCDYKYNDENTVSFVTLNKVGGFLQRMDLVRKYAFGKMLIVGSEAPFKVQGLWLFRGKEIPKFVMEEVYDMELYEWKEVDINDEAQ
KERVSQMIEDHEPFEGEALLDAKCFK*
>StTCHQD
MQLYHHPFSLNSQKVRLTLEEKGIDYTSHHVNPLTGKNMDAFFFSMNPSAKVPVFQNGSHIIYDTIEIIQYIERIAEKVSSGGNNLNLSSREVIGWM
HKIQEWDAMYFTLFHVPEKYRLCVSKFLRRVIIARMAESPDLASAYHCKLRQAYDTDDKLKNAEVLRRSENHLVRLLDEVELKLGETSYLSGEEFSL
ADVMLIPVLARIELLNLEDEYINSRPNIADYWVLVKQRPSYKKVIGKYFDGWRRRKTLLKTWCFIRVRSMLRKY*
>StMGST1
MAGVEFLPKEYGYVILALAAYCFLNFWMSFQVGKARKQYKVSYPTMYATEAENKNAKSFNCVQRGHQNSLEMMPMFFMLMIVGGIRHPLICASLGAV
YIVSRYFYFTGYSTGDPQNRLTLGKYNFLAIMGLLICAISCGVNFLMS*
>StMGST2
MRRVSRIAALYRAVDGAAAVEVPQHRMSTAAQFSTSSNKSSAKSNWLFNNLLTDLSARTAAHAVAGTMLFSVAATTLTDEVHAKEAVPPELRPKDLV
LYQYEACPFCNKVKAFLDYYDLPYKIIEVNPISKKELKWSDYKKVPVVLVDGEQMVNSSDIIDKLYEKVRSGDSTFDADEESKWRKWVDDHLVHMLS
PNIYRNTSEALESFDYITSHGNFSFTERITAKYAGAAAMYFVSKKLKKKYNITDERASLYEAAETWVDALKGRDFLGGSKPNLADLAVYGVLRPIRY
LKSGRDMVENTRIGDWYSRMESEVGVSARIQA*
>StGHR1
MSATGAFERTASTFRNIVSREPGSVFPVESGRYHLYISYACPWASRCLAYLKIKGLDQAIDFTSVKPIWERTKDSDEHMGWVFASSSTEEAGADLDP
INGAKSIRELYELASTNYSGKYTVPVLWDKKLKTVVNNESAEIIRMFNSEFNDIAENAALDLYPPHLQSQINETNDWIYDGINNGVYRCGFAKMQEP
YDEAVQKVYKALDKCEEILSKQRYICGDQVTEADIRLFVTLIRFDEVYAVHFKCNKKLLREYPNLFNYTKDIFQIPGMSSTVNMEHIKKHYYGSHPG
INPFGIIPQGPNIDYSSPHDREKFSK*
>StGHR2
MYSTHSHISSFISIPFPPSKSKTHKLKYPKILHTKLCSSTPRMSLNQNSNTNLINTITKLLWGPSLPPQLLISTVRSTWSTAWQLMMSQLAPSDPTG
SYTRPTSQFRLYSNPKLKVSPKDLHLYVGLPCPWAHRTLIVRALKGLEDSVPVSIASPGIDGSWEFRFFSDPDKDKLVPSLDKANGCKTLREVYKLR
RGGYSGRSTVPMLWDMEKKEVLCNESYDIIEFFNSGLNEIAGNPELLSPPALKDDIRKWNDIIYPNVNNGVYRCGFAQSQEAYDKAAEGLFRTLEML
EDHLGGSRYLCGDVLTLADVCLFTTLIRFDVVYNVLFKCTKKKLIEFTNLHGYLRDIYQIPKVAETCNMGQIMEGYYKILFPLNPGGINPIMPSGCE
DEVLSKPHNRDSLSLETKVVQHSVS*
